# Central Nervous System Responses of the Oriental migratory, *Locusta migratoria manilensis*, to Fungal Infection

**DOI:** 10.1038/s41598-017-10622-5

**Published:** 2017-09-04

**Authors:** Wei Zhang, Jianhong Chen, Nemat O. Keyhani, Kai Jin, Qinlv Wei, Yuxian Xia

**Affiliations:** 1 Genetic Engineering Research Center, School of Life Sciences, Chongqing University, Chongqing, 400045 PR China; 2 Chongqing Engineering Research Center for Fungal Insecticide, Chongqing, 400045 PR China; 3 Key Laboratory of Gene Function and Regulation Technologies under Chongqing Municipal Education Commission, Chongqing, 400045 PR China; 4 Department of Microbiology and Cell Science, Institute of Food and Agricultural Sciences, University of Florida, Gainesville, FL 32611 USA

## Abstract

Responses of the central nervous system (CNS) to microbial challenge and the interplay between the CNS and the immune system are important for defending against pathogen attack. We have examined the CNS transcriptional response of *Locusta migratoria manilensis* to infection by the locust-specific fungal pathogen, *Metarhizium acridum*. CNS responses were examined during spore attachment, fungal germination and pre-penetration of the cuticle, and cuticle penetration/hemocoel ingress and proliferation. Effects were seen at the earliest time points (4 h post-infection) and the number of differentially expressed genes (DEGs) was highest during late mycosis (72 h post-infection). Significantly affected neurological pathways included genes involved in serotonergic, cholinergic, dopaminergic, GABAergic, and glutamergic synapse responses, as well as pathways responsible for synaptic vesicle cycle, long-term potentiation and depression, and neurotrophin and retrograde endocannabinoid signaling. In addition, a significant number of immune related DEGs were identified. These included components of the Toll, Imd and JAK/STAT pathways, consistent with interactions between the CNS and immune systems. The activation of immune response related CNS genes during early stage infection highlights the rapid detection of microbial pathogens and suggests an important role for the CNS in modulating immunity potentially via initiating behavioral adaptations along with innate immune responses.

## Introduction

The central nervous system (CNS) integrates both external and internal sensory inputs, generating behavioral responses and regulating many physiological processes^1^. Due to its ability to quickly process information, in particular to allow for rapid responses to stress conditions, the CNS and immune systems are recognized as vital for an organism to survive biotic and abiotic stresses^2^. Pathogen infection is one of the most severe biotic stresses affecting the survival of all animals. Pathogen infection usually activates the immune system that acts to kill or inhibit growth of the invading organism, often via recognition of pathogen-associated molecular patterns (PAMPs), while the CNS is suggested to regulate innate immune response via hormonal and neuronal pathways^3^. In mammals, bacterial lipopolysaccharide (LPS) is able to directly activate CNS inflammation by binding to CNS-expressed Toll-like receptors (TLR4) without involvement of peripheral cytokines^4^. Both local acute-phase inflammatory response (redness, pain, heat) and systemic acute-phase response (fever) caused by infection, are triggered by immune mediators and cytokines released by the innate immune system that can be activated by regional neural or systemic neuroendocrine responses^3^. In addition, many immune cells are capable of responding to neural factors, and these cells can contain receptors for neuro-transmitters, peptides and hormones, and as well as their downstream signaling pathways^3^. Insufficient or hyper CNS immune-related responses can lead to uncontrolled infection or inflammation related CNS pathologies, respectively. In humans, the latter can include schizophrenia, epilepsy, autism^5^ and even death, e.g. in addition to immune-related diseases, HIV infection is known to cause a range of different CNS pathologies^6^. Overall, however, little is known concerning global CNS gene-expression responses to a fungal pathogen.

Arthropods are a species-rich order that occupies nearly 67% of the global described fauna and flora, and are important for almost all ecosystem processes^7^. Similar to effects seen in other animals, i.e. mammals, the behavior of many insects can be affected by pathogen attack. Infection of invertebrates by entomopathogenic fungi can lead to host behavioral changes including induced fever^8^, elevation seeking^9^, reduced or improved activity^10, 11^, reduced response to semiochemicals^12^ and changes in reproductive behavior^13^.

As an important agricultural pest in many parts of Asia, the Oriental migratory locust, *Locusta migratoria manilensis* is capable of devastating a wide range of crops, particularly during its gregarious-aggregation phase. Compared to mammals and other arthropods, the CNS of locusts is simpler and relatively large, respectively, and hence easy to dissect. The locust CNS consists of a dorsal collection of ganglia (brain) linked to a ventral nerve cord composed of paired segmental ganglia running through the ventral midline of the thorax and abdomen. Insect pathogenic fungi of the Clavicipitaceae family, e.g. of the *Beauveria* and *Metarhizium* genera have been used in a wide range of insect biological control applications, including towards grasshoppers and locusts^14^. Conidia of these fungi attach to the cuticle where they germinate and penetrate the exoskeleton of the insect via mechanical pressure and the production of a battery of cuticle degrading enzymes, whose products provide nutrients to the growing fungal hyphae^15^. Cuticle penetration leads to ingress into the hemocoel, where the fungi undergo a dimorphic transition, producing freely floating protoplast cells that evade host immune reactions and proliferate on the nutrients available in the hemolymph^16^. Fungal hyphae ultimately work their way out of the insect, sporulating on the cadaver. These complex natural infection and host response system represents an ideal pathogen-host interaction model to examine CNS responses of a host to its specialized fungal pathogen.

RNA-seq is a high-throughput sequencing technology useful for investigating host-pathogen interactions by determining relative expression of genes, particularly during the infection process^17, 18^. The goal of this research was to analyze the transcriptomics response of the locust CNS to *M. acridum* infection. RNA-seq was used to identify relative genes expression differences between infection and control groups pairwise over six representative infection stages. These functional analyses were used to provide global gene expression responses concerning (1) the locust CNS response to fungal infection at representative infection stages, and (2) CNS transcripts implicated in immune regulation.

## Results

### Stages of M. acridum infection of L. migratoria

**Figure 1:**
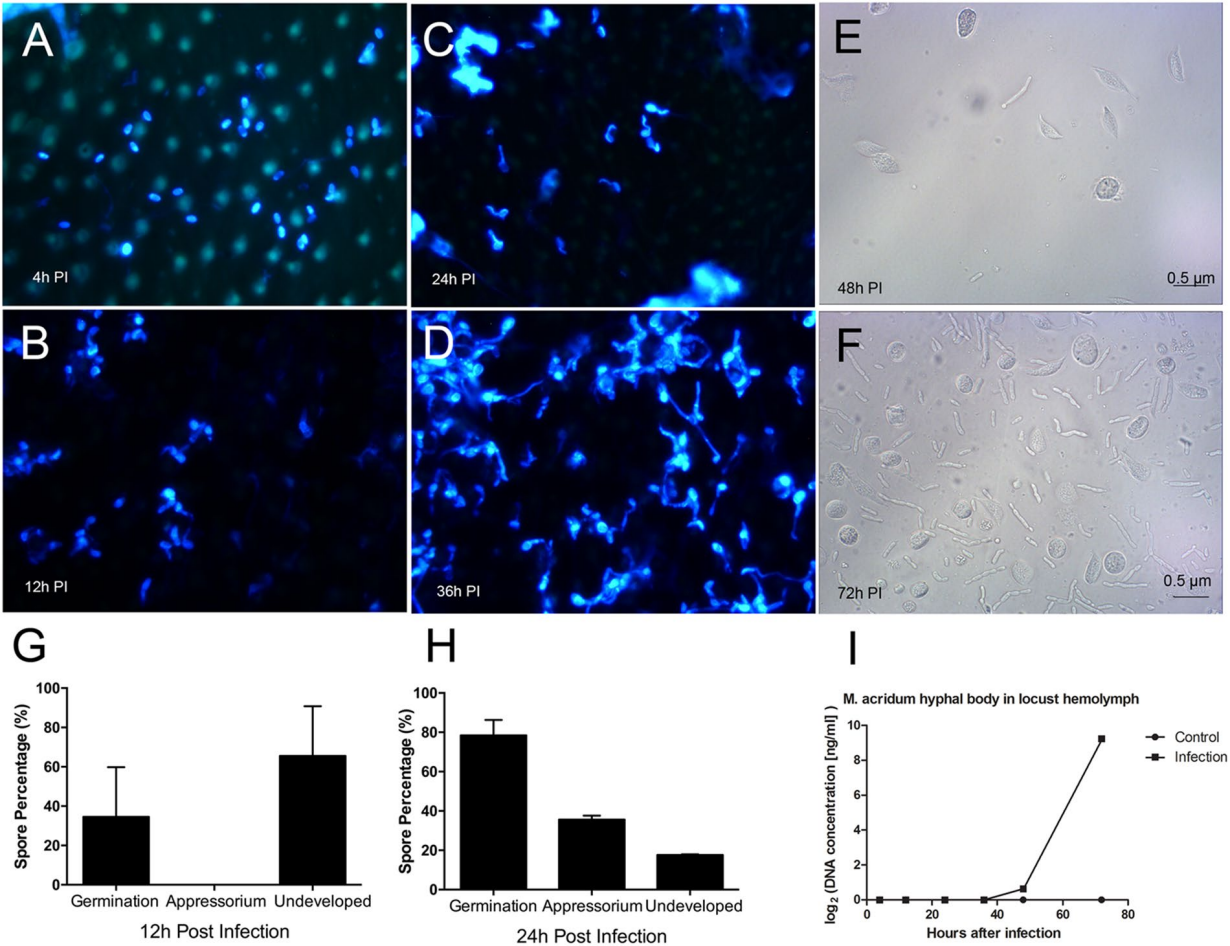
*M. acridum* infection stages identification to locust by natural inoculation. (**A**–**D**) Images of CFW stained *M. acridum* on locust epidermis over the indicated time course. Percentages of germinated spores, appressoria and undeveloped spores 12 hours pi (**G**) and 24 hours pi (**H**). Microscope observation of hyphal bodies on the hemolymph of locust on 48 hours PI (**E**) and 72 hours PI (**F**). DNA concentration of *M. acridum* in locust hemolymph on different infection time points (**I**).

The process by which entomopathogenic fungi infect their insect hosts can be divided into several stages^15, 19^. These include (1) adhesion to the host surface, (2) germination on the surface, (3) cuticle penetration/initial ingress into the hemocoel, (4) mycosis, and (5) outwards growth and sporulation on the cadaver. Samples derived from the final stage were omitted as the insect is essentially dead or near death, the fungus has invaded most tissues, and significant apoptosis and cell death of the host has occurred. For our trancriptomics study, progress through each stage was monitored via microscopic visualization of samples. *M. acridum* was examined on the surface of locust cuticles via staining of fungal membranes using calcofluor white (CFW, Fig. 1A–D). At 4 h post-inoculation (pi), fungal spores could be seen distributed throughout the surface of the insect cuticle, with little to no germination evident (Fig. 1A). Within 12 h pi, ~50% of the conidial spores had germinated, however, no appressoria (swelling at the tips of the germ tubes, were seen (Fig. 1B and G). By 24 hr pi, 80–90% germination rates were seen, with ~40% of the cells showing distinct appressorial formation (Fig. 1C and H), and by 36 h extensive hyphal growth and branching was evident (Fig. 1D; distinct appressoria could no longer be observed). Inspection of the insect hemolymph at 48 h pi indicated the presence of a few *in vivo* fungal hyphal bodies (Fig. 1E), with significant proliferation of these cells seen 72 h pi (Fig. 1F). Using primers designed to the *M. acridum* ITS (Internal Transcribed Spacers) sequence, absolute real-time quantitative PCR of locust hemolymph samples taken over the described time course indicated the absence of any fungal cells in the hemolymph in the early time points (4–36 h), with initial detection of fungal DNA in the hemolymph 48 h pi, followed by rapid amplification within 72 h pi (Fig. 1I). These data corroborated the histological results, allowing for establishing the infection stage parameters for the gene expression studies.

### Sample preparation, gene assembly and annotation

**Figure 2:**
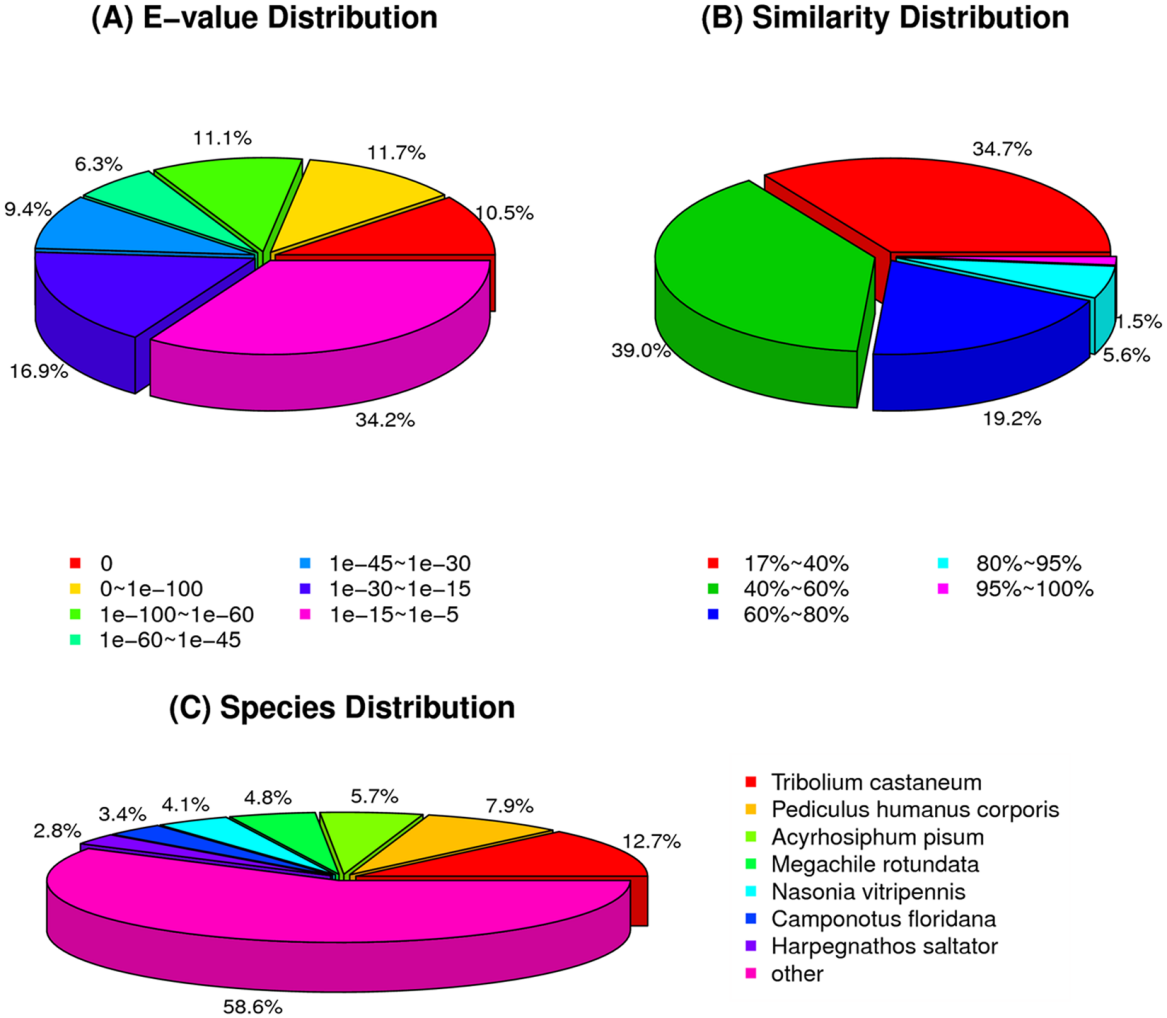
Summary of transcriptome analyses. (**A**) E-value distribution of BLAST hits for each unique sequence. (**B**) Similarity distribution of the top BLAST hits each sequence. (**C**) Species distribution shown as a proportion of the total homologous sequences with an E-value > e^−25^ using the first BLAST search hit for each sequence in the analysis.

A total of twelve RNA samples: six corresponding to time points of *M. acridum* infection of *L. migratoria*; i.e. at 4, 12, 24, 36, 48, and 72 h post-inoculation, and six corresponding to controls mock-treated with buffer alone at the same time points, were prepared, respectively. The CNS tissues, i.e. neuronal ganglia and the ventral cord, of infected and control locusts were dissected, rinsed, and the RNA in these tissues was extracted and purified, and subsequently used to construct the cDNA libraries for sequencing as detailed in the Methods section. Samples were sequenced using the Illumina platform, and a total of 85.37 Gb clean nucleotides were generated from the twelve samples. The Q20 and GC percentages of samples ranged from 97.6–97.9 and 38.0–41.1, respectively (Table S1), indicating a high quality score dataset. In order to increase assembly sequence lengths, 41 locust sequence read archive (SRA) datasets were downloaded from NCBI and combined with 24 SRA datasets (Y. Xia, unpublished results). These 77 SRA datasets were used to provide a query dataset after assembly that included trimming of any adaptor sequences, and removal of ambiguous and/or low quality reads (Q20 < 20). An overview of the sequencing results is given in Table S1. Total nucleotides assembly reached 306.4 G, representing an ~122-fold coverage of the locust genome (~2.5 G). The final assembled sequences were submitted to the NCBI Transcriptome Shotgun Assembly (TSA) Database under the accession #: GETS00000000. A total of 128,998 unigenes were generated with a mean length of 920 bp. A summary of the total number of contigs, initial unigenes, and the final assembled unigene set, as well as mean lengths, consensus sequences, distinct clusters, and singletons is given in Table S2. The assembled unigene set (128,988) was queried against various databases including the Nr (non-redundant protein databases, E-value cut-off = 10^−5^), SwissProt, KEGG (Kyoto Encyclopedia of Genes and Genomes) and COG (Cluster of Orthologous Groups), resulting in 38,567, 28,826, 26,186 and 15,139 unigenes that could be assigned and/or annotated, within each database respectively. The combined set of unigenes that could be annotated and/or assigned was 43,448 (33.7%) with the E-value and similarity distributions, and top hits within the Hexapoda given (Fig. 2).

### Differentially expressed unigenes of the *L. locusta* and their expression tendency to fungal infection

A pairwise analysis of the differential expressed genes (DEGs) during the infection/sampling time course between controls and infected samples was performed. Based on a cut-off of ≥2-fold change in expression, a gradual increase in up-regulated DEGs were seen in the locust CNS after fungal infection from 4–24 h pi, with a noticeable dip at 36 h pi, followed by a sharp increase during 48–72 h pi (Table 1). DEGs showing a ≥2-fold down regulation followed a similar pattern although less although the dip extended to the 48 h pi time point and only sharply increased at 72 h pi. Using a more stringent DEG cut-off level of ≥5-fold, representing the set of highly differentially expressed genes, the numbers of DEGs were reduced but showed a similar time change tendency as those of DEGs with a cut-off level of ≥2-fold (Table 1). The numbers of differentially expressed genes at 12h–24h pi and 72 h pi are much higher than at 4 h pi, 36 h-48 h pi, indicating CNS responses to fungal infection at various infection stages are obvious different. In addition, much more down-regulated genes appeared at all six infection stages than up-regulated genes (Table 1), suggesting majority of the transcription of DEGs were inhibited and it could be a reason that locust is not able to overcome the infection of *M. acridum* ultimately.

**Table 1:** Differentially expressed unigenes on different infection stages.

Time (pi)	4 h	12 h	24 h	36 h	48 h	72 h
≥2-fold up-regulated	165	487	482	212	683	1353
≥2-fold down-regulated	6591	9320	10092	6796	5665	10664
Total	6756	9807	10574	7008	6348	12017
≥5-fold up-regulated	12	30	31	16	88	148
≥5-fold down-regulated	189	396	660	227	146	823
Total	201	426	691	243	234	971

**Figure 3:**
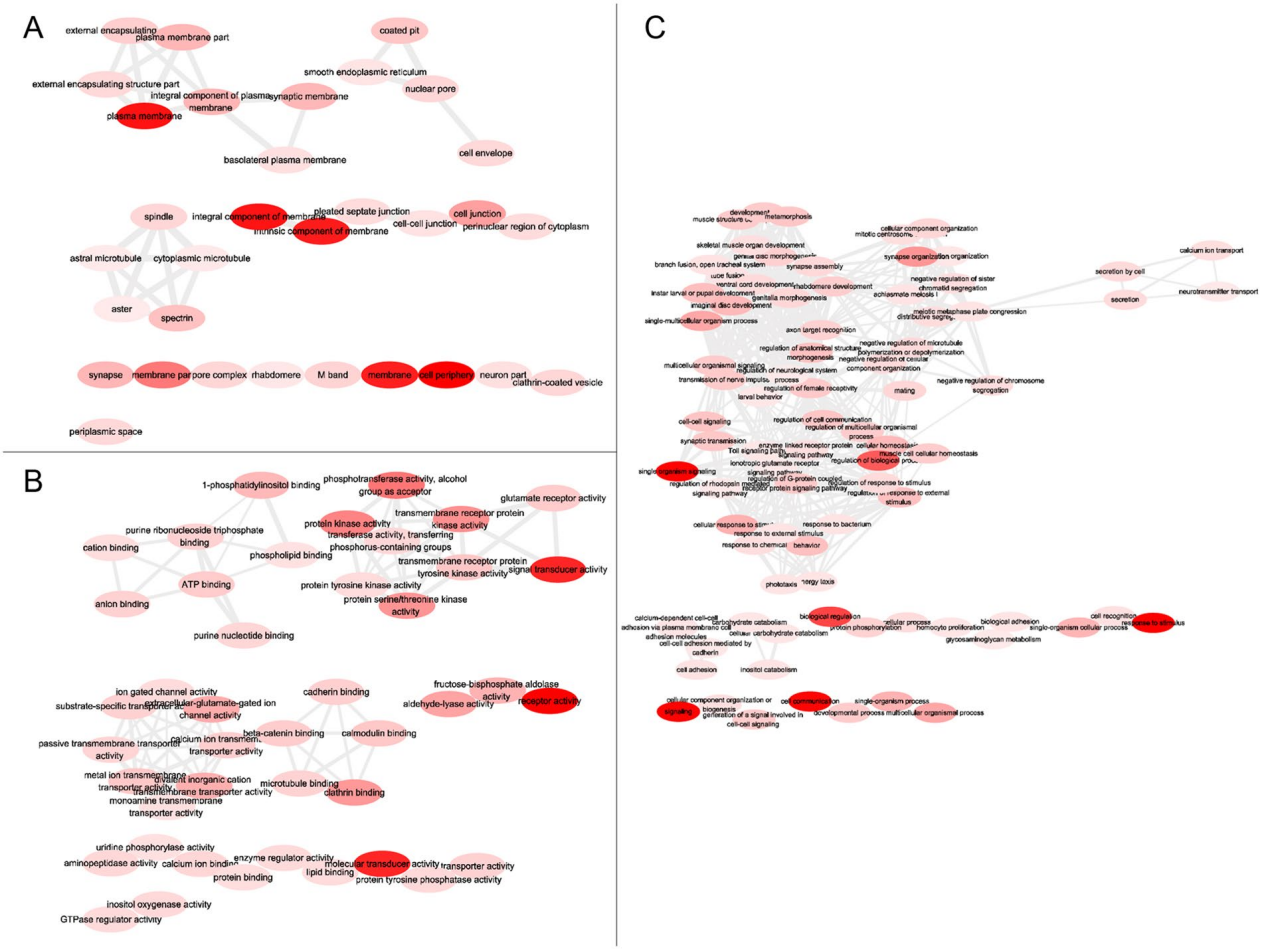
GO term relationships within the unique (Fig. 3A) cell component, (Fig. 3B) molecular function, and (Fig. 3C) biological process terms over time course of fungal infection of the locust. Circle color intensity indicates the confidence of the p-value (lowest is red, highest is light pink). The circle size indicates the frequency of the GO term in the underlying GOA database. Highly similar GO terms are linked by edges in the graph, where the line width reveals the degree of similarity.

Gene Ontology (GO) enrichment analysis, including three functional groups (biological process, cellular components, and molecular functions), was performed according to the hypergeometric test with a p-value < 0.05 (Tables S3–S5). In order to construct treemaps reflecting representative enrichment terms throughout the data time courses and reflect their relationship, a set of interaction GO term maps were produced by combining the enriched GO terms for each time point for each of the three different GO categories, i.e. cell component (Fig. 3A), molecular function (Fig. 3B), and biological process (Fig. 3C) by REVIGO (http://revigo.irb.hr/)^20^. In these figures each circle represents a GO term subcategory and the size of the circle is proportional to the combined number of times the term is represented in the time points. Lines between circles reflect functional connections between subcategories. These data indicate that cell component terms, particularly within plasma membrane, integral component of membrane, intrinsic component of membrane, membrane and cell periphery related sub-categories are universally enriched. Within molecular function, terms corresponding to signal transduction, molecular transduction, and receptor activity universally enriched and in the GO biological process classification, single organism signaling, regulation of biological process, signaling, response to stimulus, and cell communication were the most commonly identified terms. These universal enriched terms could be related to CNS functioning related to a wide range of processes including signal reception and transduction, and neurological regulation of biological process in response to *M. acridum* infection throughout all stages examined.

For each enriched GO category specific enriched terms at each time point were further analyzed online (http://bioinformatics.psb.ugent.be/webtools/Venn/). For cell component category (Table S3), the main specifically enriched terms at 4 h pi are 3 of vesicle and 3 of pore or pit related items. 12 h pi, 4 of cytoskeleton related cell components and 1 of perinuclear region of cytoplasm specifically enriched. Anchoring junction and junction are the only two specifically enriched items at the 24 h pi. No terms of cell component specifically enriched at 36 h pi. Terms of M band and spindle specifically enriched at 48 h pi. 2 of encapsulating structure, 2 of periplasmic space and 2 of membrane related cell components specifically enriched at 72 h pi. These specifically enriched cell components were related to distinct infection structures or processes.

Similarly, the specifically enriched category of molecular function (Table S4) at the 4 h pi included 10 corresponding to ribonucleoside related binding terms and 4 to other binding terms (ATP, anion, protein and 1-phosphatidylinositol). At 12 h pi: 3 terms mapped to calcium channel, 4 to binding (beta-catenin, cadherin, microtubule, cation) and 2 to phosphatase activity related terms. At 24 h pi: no mapped terms identified. At 36 h pi: one terms to ryanodine-sensitive calcium-release channel activity. At 48 h pi: 2 terms mapped to growth factor-activated receptor activity related terms, 1 to aldehyde-lyase activity and 1 to fructose-bisphosphate aldolase activity. At 72 h pi: 10 terms mapped to ligand-gated ion or other channel activity, 3 to substrate-specific or transmembrane transporter activity, and 2 to glutamate receptor activity related terms. These results indicate that transcripts representative of specific molecular functions were enriched in locust CNS at various *M. acridum* infection stages.

For the specifically enriched biological processes (Table S5), at 4 h pi, 12 various biological processes were specifically enriched, including genes involved in pathways impacting signal transmission, neurotransmitter secretion, short-term memory, conditioned taste aversion, and protein kinase cascade. At 12 h pi, as many as 72 terms were specifically enriched, including 8 of cell or other morphogenesis, 6 of cell adhesion, 4 of muscle tissue development, 4 of neuron differentiation, 4 of axon guidance, 3 of ion transport, 3 of microtubule depolymerization, 3 of cellular or anatomical structure homeostasis, 2 of photoreceptor cell development related terms, etc. At 24 h pi, the majority (6 of 7 total) of the specifically enriched terms were related to morphogenesis or fusion of a branching epithelium, branching tracheal or other structures, and to wing disc morphogenesis, suggesting a response of CNS related to epithelium penetration. At 36 h pi, cellular carbohydrate catabolic and inositol metabolic processes represented two specifically enriched terms, which could be related with fungal nutrition deprivation to the host. At 48 h pi, 15 terms related to chromosome organization, metaphase or meiosis and 6 to genitalia morphogenesis were specifically enriched. At 72 h pi, 6 terms related to morphogenesis, 6 to development, 5 to behavior, 4 to cognition, learning or memory, 3 to stimulus response, 3 to signaling transduction, 2 to microtubule organization, were specifically enriched. Of note one term of the Toll signaling pathway was specifically enriched in the 48 h pi time point. In addition, a number of transcripts involved in behavior related biological processes were enriched at several infection stages either specifically or commonly, including courtship behavior at 4 h pi, mating behavior, larval behavior, regulation of behavior and reproductive behavior at 12 h pi and single-organism behavior, mating behavior, adult behavior, multi-organism behavior, reproductive behavior and adult locomotory behavior at 72 h pi. These results suggest specific biological processes are involved in the response of the locust CNS to various stages of the fungal infection process.

### CNS process-related pathways and their expressions during fungal infection

**Figure 4:**
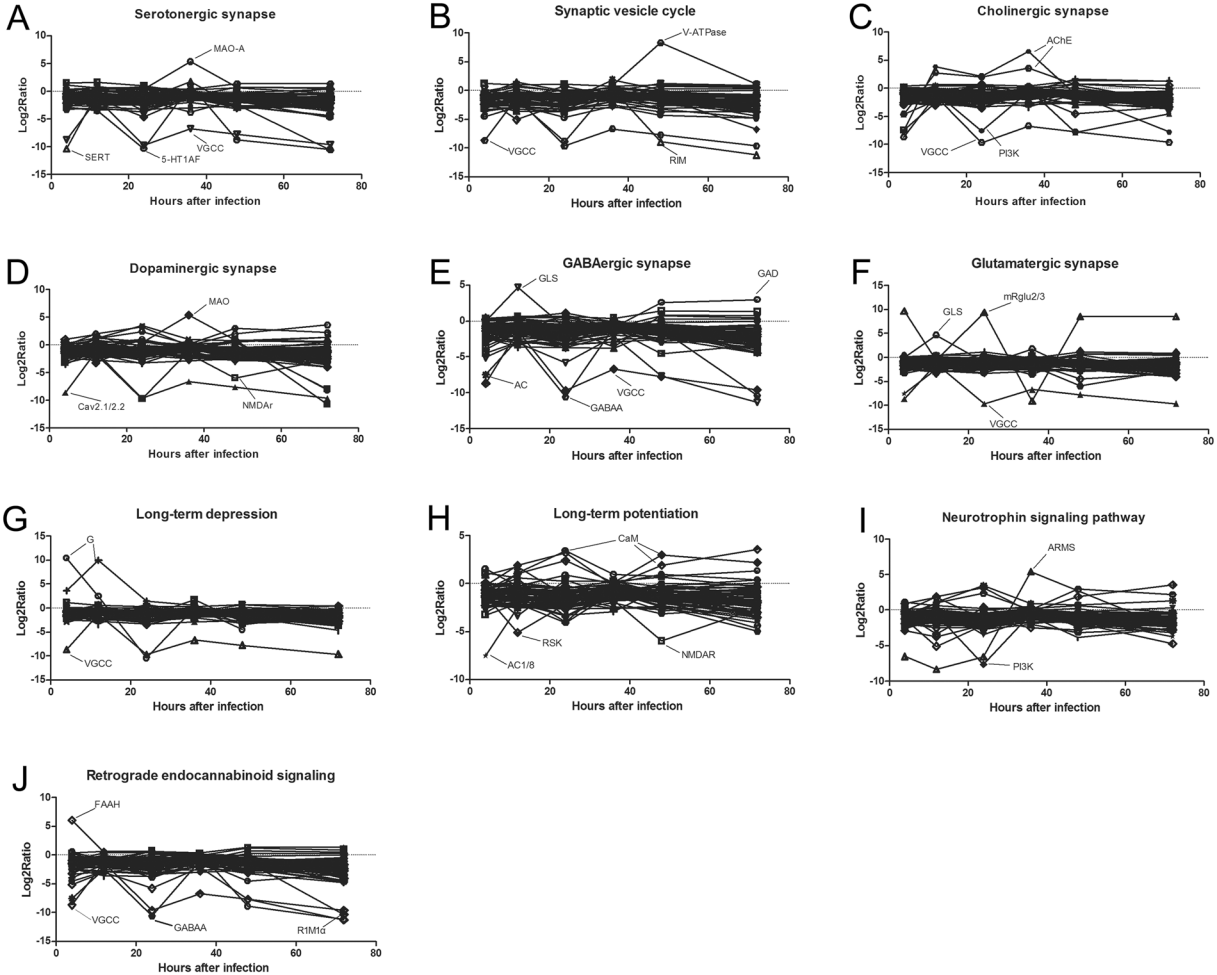
The log2ratios of the locust CNS differentially expressed unigenes as a function of the infection time. DEGs were sub-categorized into those involved in processes related to (**A**) the serotogenic pathway, (**B**) the cholinergic pathway, (**C**), the dopaminergic pathway, (**D**), the gamma-aminobutyric acid (GABA)-ergic pathway, (**E**) the glutamatergic pathway, (**F**) the synaptic vesicle cycle (**G**) long-term depression, (**H**) long term potentiation, (**I**) neurotrophin signaling, and (**J**) retrograde endocannabinoid signaling. Highly (log_2_ratio value > 5) DEGs are labeled. As some DEGs participate in multiple sub-categories as defined above, they are included in each respective process/graph in which they participate.

A set of DEGs (692 total) were further identified in a variety of neuro-related processes in response to fungal infection including neuroreceptor-related serotogenic (59 unigenes/collapsed to 20 unique gene name), synaptic vesicle cycle (58/15), cholinergic (74/18), dopaminergic (88/25), gamma-aminobutyric acid (GABA)-ergic (64/15), and glutamatergic (80/20)- synapse pathways, as well a DEGs involved in the long-term depression (59/15) and potentiation (69/16), neurotrophin signaling (74/33), and retrograde endocannabinoid signaling (67/15). The distribution of these DEGs over the time course of infection is given in Fig. 4. The analysis of CNS related pathways throughout the infection course shows that most of the major CNS pathways are affected. Neurotransmitter pathways including those involved in serotonin, GABA, and dopamine, showed a general down-regulation, although spikes in the expression of specific genes were noted. Exceptions were seen for the acetylcholine pathway in which early and late down-regulation was seen, however, pathway members were generally up-regulated between 12–48 h post-infection and the glutamergic pathway that showed an oscillation over the time course of infection. Genes involved in long-term depression displayed a trend toward early up-regulation followed by down-regulation or return to baseline. Additional mixed responses were seen for genes involved in neurotrophin signaling, retrograde endocannabinoid signaling, long-term potential and synaptic vesicle cycling.

Of the DEGs identified in the serotinergic pathway, 56 unigenes clustered with a log_2_ratio value between −5 to 5 and the absolute value of the remaining 4 exceeded 5 (considered as “highly” DEGs, Fig. 4A). These latter DEGs encoded for monoamine oxidase A (MAO-A), serotonin (SERT), the 5-hydroxytryptamine (serotonin) receptor 1A (5-HT 1AF), and the voltage-dependent calcium channel P/Q type alpha-1A (VGCC). Within synaptic vesicle functioning 59 unigenes showed log_2_ratio values between −5 to 5, with 3 DEGs showing greater differential expression. The latter category included the V-type H+−transporting ATPase subunit a (V-ATPase-a), regulating synaptic membrane exocytosis protein 1 (RIM), and VGCC (Fig. 4B). Of the 74 DEGs involved in the cholinergic pathway identified, two highly DEGs were identified; the PI3K (phosphoinositide-3-kinase, regulatory subunit (PI3K) and VGCC (Fig. 4C). With respect to the dopamine pathway, three highly DEGs were seen including the voltage-dependent calcium channel P/Q type alpha-1A (Cav2.1/2.2), the ionotropic glutamate receptor-N-methyl D-aspartate 2 A (NMDAr), and MAO (Fig. 4D). GABA pathway highly DEGs included; glutaminase (GLS), glutamate decarboxylase (GAD), adenylate cyclase 1 (AC), GABA A receptor, alpha 1 (GABAA), and VGCC (Fig. 4E). Highly DEGs that formed part of the glutamaturgic pathway included metabotropic glutamate receptor 2/3 (mRglu2/3), GLS, and VGCC (Fig. 4F). Highly DEGs involved in long term depression included guanine nucleotide-binding protein subunit alpha-13 and VGCC (Fig. 4G), and highly DEGs that participate in long term potentiation included CaM (calmodulin (CaM), p90 ribosomal S6 kinase (RSK), AC and NMDAR (Fig. 4H). Neurotrophins signaling pathways genes that were highly differentially expressed included ankyrin repeat-rich membrane spanning protein (ARMS) and PI3K (Fig. 4I). Endogenous cannabinoids (endocannabinoids) are synaptic retrograde messengers and although no transcripts were identified as homologous to either of the vertebrate cannabinoid receptors, some other members of this pathway were identified. Within this group of DEGs identified, those that were highly differentially expressed included; FAAH (fatty acid amide hydrolase), regulating synaptic membrane exocytosis protein 1 (R1M1), GABAA, and VGCC (Fig. 4J). The relationship of these highly DEGs between CNS and immunity is further analyzed in the discussion section.

### Quantitative real time-PCR validation of the transcriptomics results

**Figure 5:**
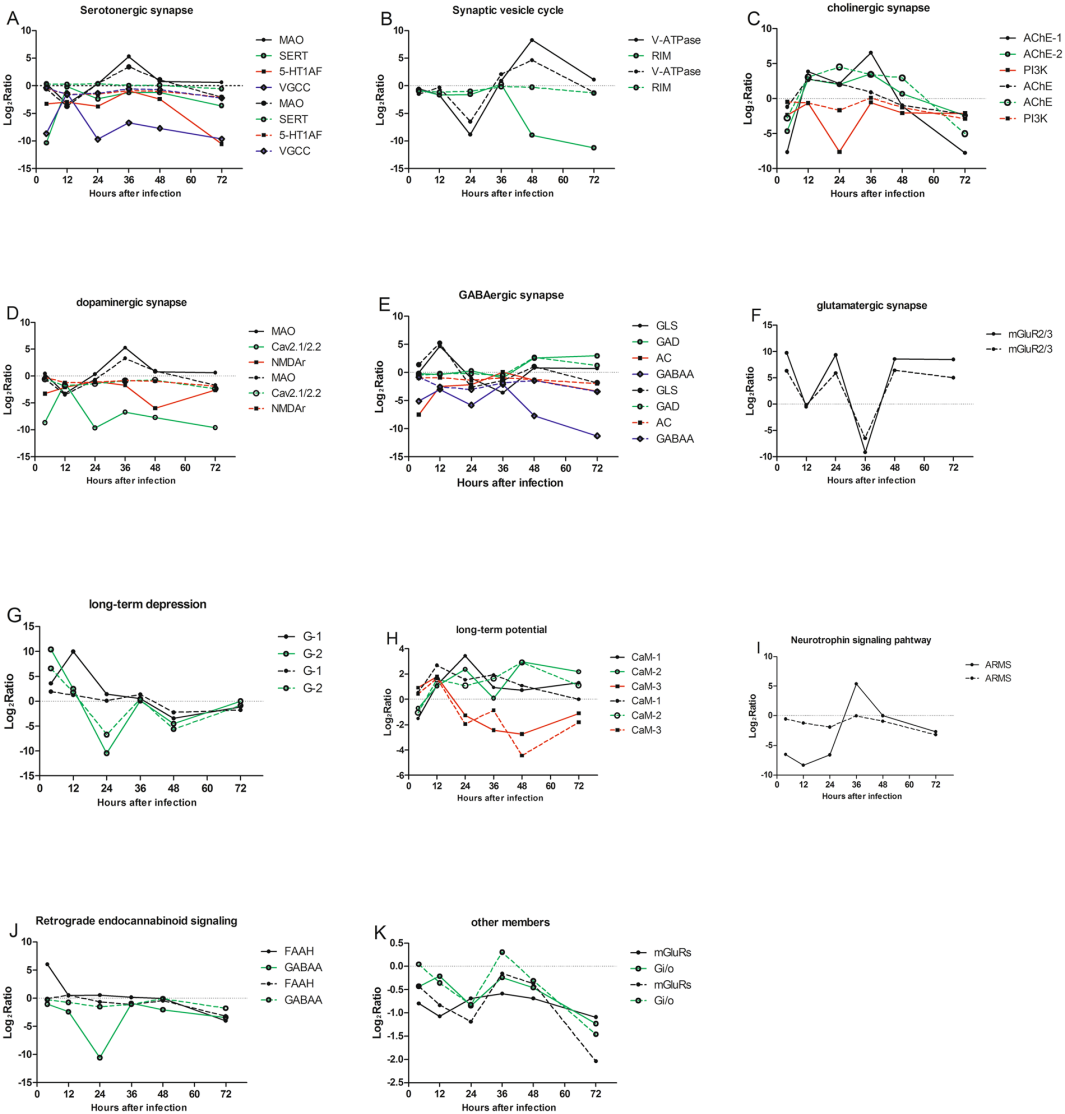
Comparison of quantitative Real Time-PCR and RNA-seq expression of select genes, data derived from the transcriptomic DEGs data (solid lines) and qRT-PCR results (dashed lines).

Majority of those highly DEGs in each CNS pathways and 2 lowly differentially expressed DEGs (mGluRs and Gi/o) as identified in the transcriptomics analyses were chosen for validation by quantitative RT-PCR. qRT-PCR primers were designed to these chosen 27 genes (Supplemental Table S4). These 27 genes were verified over all 6 time-points after *M acridum* infection (Fig. 5A–K). Significant differentially expressed genes were defined as the absolute value of log2ratio is over 1 in RNA-seq results or 0.5 in qRT-PCR results. Of four genes; MAO-A, SERT, 5-HT1AF, and VGCC, examined by qRT-PCR that participate in the serotonergic pathway, SERT is not so agreement with RNA-seq results, with the exception of second and last time points (Fig. 5A). The first and last time point of MAO and VGCC were not consistent with RNA-seq result, respectively (Fig. 5A). FAAH, involved in the retrograde endocannabinoid-signaling pathway, had three time points (4 h, 24 h and 36 h pi) that were not in good agreement with the RNA-seq data (Fig. 5J). The majority of the other genes involved in the CNS pathways examined revealed good agreements between the RNA-seq and qRT-PCR profiles, although in several cases the levels, particularly of down-regulated genes, were not as pronounced in the qRT-PCR as compared to the RNA-seq data. Overall, the qRT-PCR results were 83.33% in agreement with the RNA-seq data.

### Immunity related DEGs expressed on CNS and their change during fungal infection

**Figure 6:**
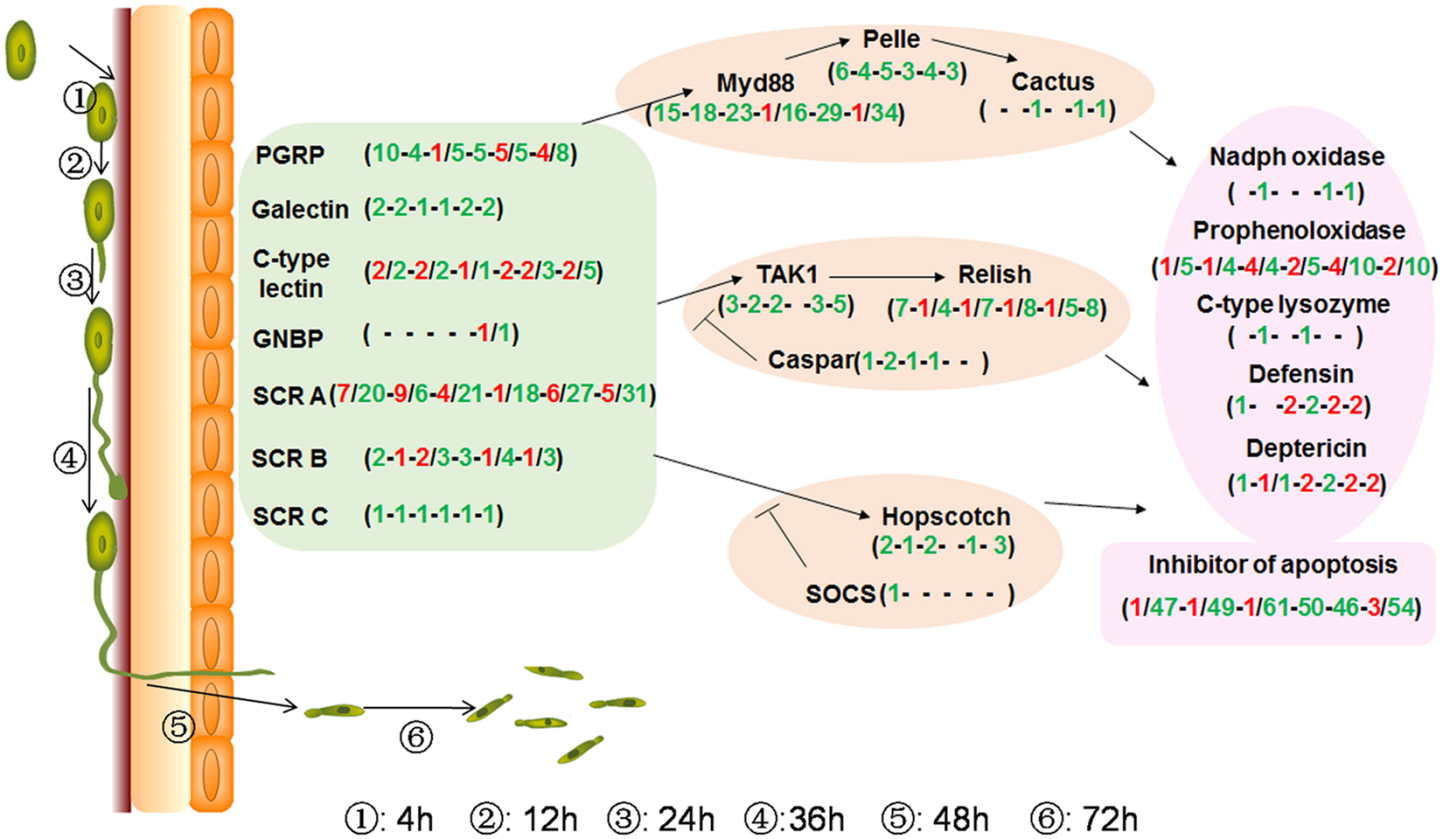
Summary of the immune related DEGs identified in the locust CNS after M. acridum infection. Red and green numbers in the brackets represent the number of significantly up-regulated and downregulated genes, respectively, corresponding to the sub-category. The infection time courses, inculding 4 h, 12 h, 24 h, 36 h, 48 h and 72 h post infection, were separated by short line in each bracket.

The innate immunity pathway can be separated into microbial recognition, signal modulation and effector release stages. A summary of the number of DEGs found in the major innate immune pathways as a function of infection time is given in Fig. 6. Related to recognition, 5 members C-type lectins were significantly differentially expressed at various infection stages, with one unigene gradually increasing its expression from −6 to 4.6. All 3 galectin genes were downregulated after infection, with 1 inhibited from 4–72 h pi. One GNBP (gram-negative binding proteins) was upregulated whereas another was downregulated at 72 h pi. In all, 17 PGRP (peptidoglycan recognition protein) unigenes were differentially expressed in the CNS after *M. acridum* infection, with 2 inhibited from 4–72 h pi and 4 activated at 48–72 h pi. As many as 37 scavenger receptor class a (SCR A) genes were differentially expressed at various infection time courses, with 4 DEGs at 4–12 h pi, 3 DEGs at 4–24 h pi, and another 4 DEGs at 48–72 h pi, all upregulated during all indicated fungal infection time points. The remaining SCR A genes were downregulated throughout the infection time point with the exception of the 12 h pi time point. Both up and down regulated DEGs appeared in scavenger receptor class b (SCR B), but only 6 in total. One scavenger receptor class c was downregulated at all tested infection time points. For the transduction processes, all identified cactus, caspar, hopscotch and socs (suppressor of cytokine signaling), 5 tak1 (Transforming growth factor beta activated kinase-1) and 9 pelle DEGs were either systematically or intermittently downregulated throughout these infection time points. Numerous myd88 DEGs (37 in total) were downregulated throughout the infection stages, with only one DEG at 24 h pi and 72 h pi respectively found to be upregulated. Fifteen relish DEGs were differentially expressed during fungal infection, with 1 relish downregulated at 4 h pi but then highly upregulated 12–48 h pi. For various effector genes, both four DEGs, 2 defensin and 2 diptericin, were up-regulated at 24 h pi, 48–72 h pi respectively. Several prophenoloxidase and 1 c-type lysozyme upregulated intermittently throughout the infection stages. Almost all of the 82 inhibitor of apoptosis DEGs identified were downregulated throughout all of the infection stages, with only 6 different such unigenes upregulated at some infection points.

## Discussion

Despite well-known behavioral effects many fungal insect pathogens can have on their hosts^21^, there are few reports that have examined invertebrate gene expression responses in the CNS particularly within the context of a specialized microbial pathogen that infects via penetration and invasion of the insect cuticle. In the *M. acridum*-locust interaction, the infection time points can be divided into: (a) spore attachment and consolidation (4 h), (b) germination on the insect cuticle surface (12 h), (c) appressorium, cuticle penetration and initial ingress into the hemocoel (24 h/36 h) and (d) mycosis (48 h/72 h).

GO enrichment analysis of specifically terms at various infection stages show different CNS global gene expression responses. The change in gene expression indicate differing CNS response strategies that could be related to changes during *M. acridum* infection that include different fungal structures, e.g. spore, germ tube, appressoria, hyphae, and hyphal bodies, changes in infection location, i.e from cuticle to internal tissues, and altered components released by *M. acridum*, e.g. cuticle degrading enzymes, and secondary metabolites/toxins. During the attachment stage (4 h pi), non-specific hydrophobins and mucilage are produced by *M. acridum* to adhere to and consolidate the interaction with the host^15^. Specifically enriched biological processes of signal transmission, neurotransmitter secretion, short-term memory, conditioned taste aversion, protein kinase cascade, and their related cell components and molecular functions suggests that signal transduction, chemical sensing, memory pathways were apparently activated in the locust CNS during the attachment stage. During fungal germination on the insect cuticle surface (12 h pi), continued adhesion, development of penetration structures, and resistance to cuticle stress, e.g. low humidity, poor availability of nutrients, antimicrobial are needed for successful infection. Conversely, recognition and elimination of the invading pathogen at this stage would represent the most likely strategy for survival of the host. Our GO enrichment analysis shows locust CNS transcripts involved in cell adhesion, cell morphogenesis or tissue development, microtubule depolymerization and cellular or anatomical structure homeostasis related biological processes were specifically enriched at the germination stage, suggesting an inability to strongly recognize the pathogen attack during this stage of the infection. During the fungal aspersorium formation and penetration stage (24 h pi), increased secretion of hydrophilic enzymes by *M. acridum* occurs in order to facilitate penetration and degrade of the host cuticle that includes hydrocarbons, fatty acids, carbohydrates, and tanned protein, also providing nutrient sources for the growing fungus^22^. At this stage, a large number of genes related to morphogenesis, epithelium and tracheal branching, and other structural components were enriched, suggesting potential structural integrity responses were regulated by the CNS to defend against penetration. At 36 h pi, cellular carbohydrate catabolic, inositol metabolic, and related biological processes were enriched, suggesting fungal initial ingress into the hemolymph triggers expression of CNS metabolic related transcripts. In addition, the enrichment of a transcript corresponding to ryanodine-sensitive calcium-release channel was observed potentially due to *M. acridum* toxin release, a phenomenon seen during fungal infection of mammals^23^. After penetration and ingress into the hemocoel (48 h pi), *M. acridum* produces single celled hyphal bodies that rapidly proliferate on the nutrients available in the hemolymph. The DEG locust CNS response at this stage appears to be centered around transcripts involved in chromosome organization, metaphase and meiosis, and genital morphogenesis related genes, raising the possibility of reproductive alterations as a response to infection. During the later stage of mycosis examined (72 h pi), the fungus has fully invaded the hemolymph and has likely begun entry into surrounding tissues in order to work its way out of the organism. During this stage, our analyses revealed a large number of locust CNS transcripts involved in morphogenesis, development, behavior, cognition, learning and memory These responses may trigger near final behavioral attempts at stopping the pathogen, e.g. heat seeking or grooming, or may be due to neurological impairment and/or degradation as a result of the extensive fungal growth.

In terms of global gene expression patterns, our data indicate that during initial hemolymph ingress and subsequent growth (i.e. ~36 to 48 h pi), DEG numbers significantly decrease. This coincides with the fungal “escape” strategy that involves formation of freely floating (in the hemolymph) protoplast-like cells known as *in vivo* hyphal bodies, that lack wall components important for host immune-recognition^24^, As the fungus begins to work its way back out in the later mycosis stage (72 h pi and beyond), the re-emergence of hyphae and other fungal cells in host^25^, might reactivate CNS responses, and our data show an increase in overall DEGs at this stage. Comparative analyses revealed transcripts involved in a number of behavioral related biological processes, including reproductive and adult locomotory behaviors, were significantly enriched at various infection stages, including 4, 12 and 72 h pi. It is known that compounds released by pathogens can affect host CNS^26^ or impair physiological systems, e.g. the respiratory system, altering host behavior^27^. Cuticular compounds including various hydrocarbons and lipids are also known to impact insect behavior, including self/non-self recognition, mate-seeking and reproduction, and task-related behaviors^22^. It is intriguing to speculate that some of the enriched locust CNS transcripts identified, could participate in mediating these altered behaviors.

A previous transcriptomics study of infection of the gregarious locust infected by *M. acridum* revealed differential expression of a number of immune-related genes, including pattern recognition proteins (PRPs, e.g. glucan recognition proteins, GNBPs), serine protease inhibitors (serpins), and anitoxidants (e.g. peroxiredoxin) rather than the production of antimicrobial compounds (e.g. production of antimicrobial peptides, AMPs) as seen during the solitary phases^28^. This transition between between solitary and gregarious phases is known to be at least partially controlled by the neurotransmitter, serotonin^29^ and/or catecholamines^30^. However, an examination of CNS responses has been lacking. Our results suggest important cross-talk between the CNS and immune system. Global gene expression patterns in the CNS responded rapidly to infection and changed as the infection proceeded. Many transcripts involved in signaling and behavior, as well as transcripts (and their protein products) directly involved in immune function and regulation were identified. These include the signal messenger, G (guanine nucleotide binding protein) that can activate members of JAK/STAT (janus kinase/signal transduction and activator of transcription), the latter important regulators of the immune response^31^. Several members of the JAK/STAT pathway were identified in our DEG dataset. Activated metabotropic glutamate receptor 2/3 is known to function in neuroprotection^32^. This gene (Glu2/3) was up-regulated at various time points of the infection suggesting a mechanism for CNS sensing of the infection and induction of neuroprotective pathways. In contrast, over-expression of endogenous FAAH (fatty-acid amide hydrolase) in *Arabidopsis* leads to increased susceptibility to bacterial pathogens^33^, opening the possibility that the fungus attempts to manipulate the host to increase infection. Glutaminase has been implicated as protecting against immunological challenge^34^ and its high expression 12 h post infection suggests that the CNS attempts to enhance immune functioning. Acetylcholine levels contribute to immune activation control^35^ but also responds to stress, with our data suggesting immune activation and stress response appeared 12–36 h after infection. Calmodulin (CaM) participates in inflammation^36^, immunity responses^37^, and promotes immune signaling via control of nitric oxide production^38^. The CaM gene was up-regulated 24–72 h post-infection suggesting active CNS modulation of immunity throughout the infection. A number of potential phagocytic responses, presumably within specialized CNS cells (astrocyte-like) were also noted. Through interaction with interferon-inducible transmembrane (Ifitm) proteins, V-ATPases promote the subcellular localization of clathrin, involved in clathrin-mediated phagocytosis^39^. Potential tissue damage mediated by the expression of ankyrin repeat-rich membrane spanning protein (ARMS), activated in allergic airway challenge^40^, and monoamine oxidases (MAOs) that catalyzes oxidative deamination of monoamine in mitochondrial was seen. In particular increased MAO activity can cause mitochondrial damages and neurodegenerative disturbances^41^. *GABAA* receptors expressed in T cells inhibit T cells response to antigen both *in vitro* and *in vivo* ^42^. The decrease in *CABAA* expression throughout infection may suggest that it might act as an intermediate receptor regulating immunity in both CNS and immune tissues.

A number of transcripts directly implicated in immunity genes were identified as differentially expressed in the locust CNS post *M. acridum* infection, indicating their potential functions in CNS protection. As seen for neurological responses, immune–related genes were affected early during the infection period and remained altered throughout the infection. Critical differentially regulated immune-related genes included those involved in pattern recognition, Toll-like receptors, and transcripts involved in inhibition of apoptosis pathways. Among them, microbial recognition genes, especially C-type lectin and SCR A and PGRP and effectors genes, e.g. prophenoloxidase, defensin and deptericin were up-regulated in the locust CNS after *M. acridum* challenge. SCR A is a multifunctional lipoprotein receptor involved in host defense, atherosclerosis and disorders of the central nervous system that is expressed both in the nervous system and peripheral tissues^43^. SCR B is expressed in microglia, macrophages, microvascular endothelium, cardiac and skeletal muscle, adipocytes, and platelets, and functions in the removal of apoptotic cells, inflammation, and is known to mediate free radical production and tissue injury in cerebral ischemia^44^. In addition, PCRP-LC has been shown to be essential for induction and sustained expression of homeostatic synaptic plasticity and it could be a candidate receptor for regulating trans-synaptic signaling.

The activation of several PGRPs in our data suggested potential cross talk between the CNS and immune system. Several immune effector factors were significantly upregulated in the locust CNS post fungal infection. In addition, genes involved in immune modulation were upregulated in the locust CNS in response to the fungus, these included; Myd88 (death domain containing myeloid differentiation factor 88) that functions in the Toll pathway, and Relish, a member of the Imd pathway. Interestingly, Myd88 has been shown to participate in immune activation after bacterial infection of the CNS in mice^45^. Cactus, another immune component, has also been observed within the CNS of *Drosophila* ^46^ although it’s expression was not affected by bacterial challenge^47^. In mice, TAK1 appears essential in CNS autoimmunity^48^ and over-expression of Relish in glial cells leads to neurodegeneration^49^. The changes in expression of these genes indicate immune-related gene expression in the locust CNS during fungal challenge, however, this response is distinct from that seen in locust immune tissues, e.g. the fat body and hemocytes after infection by the same pathogen^50^. These latter results also revealed that differential strategies are adopted the fat body and hemocytes^50^, thus, although some overlap exists, all three tissues are altered in the expression of different components of the immune response. Our results provide a dataset that defines pathways that can now be further explored with respect to their roles in immunity, pathogen sensing and defense, and the behavioral adaptations and consequences that occurs during infection. The nature of *M. acridum* as a specific pathogen of locusts also highlights its evolutionary adaptations to counter the locust defenses. Increased understanding of the molecular and biochemical interplay between the host and pathogen can be exploited to improve the biocontrol potential of the fungus.

## Methods

### Insects, fungal strains, inoculation protocol and dissection of locust central nervous tissues

*Locusta migratoria manilensis* (Orthoptera: Acrididae), were reared in metal cages at 30 ± 3 °C with 70–75% relative humidity and a photoperiod of 16 h light, 8 h dark, and supplied with fresh wheat shoots and wheat bran. Adult males of the *L. migratoria* were collected 24 hours after the fifth (final) ecdysis. These synchronized adult males were used in all experiments. The locust-specific insect pathogenic fungal strain *M. acridum* CQMa102 (CGMCC No. 0877, China General Microbiological Culture Collection Center) was used in insect inoculations. Briefly, *M. acridum* was cultivated on one-quarter strength Sabouraud dextrose agar (SDA) for 15 days at 28 °C. Spores were collected by flooding plates with distilled deionized H_2_O containing 0.05% Tween 80 and the fungal spore suspension was subsequently filtered through sterile lens paper to remove mycelia and clumps. The concentration of spores in the filtrate was determined by a Neubauer haemocytometer, adjusted to 1 × 10^8^ conidia using ddH_2_O/0.05% Tween 80. Adult healthy males were inoculated at the locust pronotum with 5 µl of the conidial suspension and control insects were treated with the same volume of 0.05% Tween 80. After specific time intervals post-inoculation (4, 12, 24, 36, 48, and 72 h), 30 locusts/time points/infected versus control were placed on ice and dissected in a Petri dish on ice in locust physiological saline solution (LoPS, 150 mM NaCl, 10 mM KCl, 4 mM CaCl_2_, 2 mM MgCl_2_, 4 mM NaHCO_3_, 5 mM 4-(2-hydroxyethyl)−1-piperazine ethanesulphonic acid pH 7.2, 0.1% Ficoll) to remove central nervous tissues extending from the head and including subesophageal ganglia and the vental cord and associated ganglia. Dissected CNS tissues were immediately placed on 1 ml RNA later buffer (Life Sciences, Ambion). Before RNA extraction, tissues were washed once with DEPC (Diethylpyrocarbonate) water and immediately transferred to mortars containing liquid nitrogen for RNA extraction.

### Determine the inoculation stages

The progress of fungal growth on the surface of the locust pronotum was monitored microscopically including via straining of fungal cells using calcofluor white (CFW). Locust dissected pronota were dissected over a time course stained (20–50 μl of 50 μg/ml CFW in 10% KOH solution) on the surface of glass coverslips, and excess solution was absorbed by a clean tissue. Samples were heated at 95 °C for 3 seconds and observed using a fluorescence microscope (Olympus BX-61, excitation filter (BP360–370), dichroic mirror (DM400), and emission filter (BA420–460)). Fungal germination and appressoria formations were monitored microscopically. The fungus was considered germinated when the germ tube was equal or greater than the width of the spore (≥3–5 µm) and appressoria formation was scored a positive when a clear swelling at the tip of a growing germ tube was evident. For each treatment stage at least five different areas of each dissected pronotum and at least five different locust pronota were examined. Each treatment was then repeated at least three times and the average numbers (germination, appressoria formation) were calculated for each treatment. Quantitative real time PCR (qRT-PCR) was employed to identify the concentration of *M. acridum* in the locust hemocoel. For each replicate, hemolymph samples from 20 different locusts for each time pointwere collected by piercing the hindleg and harvested 100 μl of the hemolymph from each locust in ice cold anticoagulant buffer (AC buffer: 98 mM NaOH; 180 mM NaCl; 17 mM EDTA (free acid); 41 mM citric acid; 440–450 mOs/Kg; pH 4.5) and subsequently used to extract genomic DNA. Three biological replicates were performed. *M. acridum* DNA from locust hemolymph was extracted and quantified by absolute quantitative real-time PCR (qRT-PCR) as previously described^51^. Briefly, hemolymph was pretreated with the ratio of 2 µL of proteinase K (20 µg µL) and 2 µL of SDS (10%, w/v) added to 20 µL of haemolymph in a 1.5 mL tube before incubation at 50 °C for 30 min and then centrifuged at 12,000 × *g* for 10 min. The collected pellet was washed once using 100 µL of 0.1 M citrate-phosphate buffer (pH 6.0) and then digested at 34 °C for 2 h with 20 µL of digestion buffer, containing 5 µg/µL of Glusulase and 5 µg/µL of Driselase. After treatment, the genomic DNA of *M. acridum* was extracted using a microspin column and the Fungus Genomic DNA extraction kit (BioFlux, Tokyo, Japan). DNA was eluted from the column in 10 µL of sterile water ultimately. The specific primers P5 (5′-TGGCATCTTCTGAGTGGTG-3′) and P6 (5′-CCCGTTGCGAGTGAGTTA-3′) were designed in terms of the sequences of the internal transcribed spacers (ITS1-5.8 s-ITS2) of the ribosomal RNA gene (rDNA) of *M. acridum*. Standard curves were generated by using *M. acridum* purified genomic DNA.

### RNA extraction and sequencing

For the locust CNS tissues, three biological samples for each time point (infection and control) were prepared. Each biological replicate was designed to consist of three technical replicates (10 locusts/technical replicates/treatment and control). One technical replicates from each biological replicate was pooled into one sample that was used for RNA-seq library construction. The remaining two technical replicates from the biological replicates (6 samples in all) were pooled and used for qRT-PCR experiments. Total RNA was extracted using the Trizol Reagent (Invitrogen) according to the manufacturer’s instructions. Prepared RNA samples were further digested with 10 units of DNase I (Takara, China) for 1 h at 37 °C to remove residual genomic DNA. The quality of the RNA samples was examined via absorbance at 260 nm/280 nm, using a Nanodrop ND-1000 spectrophotometer (LabTech, USA). RNA libraries were constructed and sequenced as previous described^50^. Raw sequencing data were produced using an Illumina HiSeq. 2000 system by a commercial facility (BGI-Shenzhen, Shenzhen, China).

### Data Analysis

Sequences were cleaned by removal of adaptor sequences, empty reads, and low quality reads, and all remaining clean reads were assembled with our previous locust transcriptomics dataset^50^ by Trinity (release 20130225)^52^ with default parameters, except for min_kmer_cov and min_glue set at 3. The raw transcriptome data were submitted to SRA database (NCBI) with accession number: SRX1672393 (4 h control), SRX1672394 (12 h control), SRX1672395 (24 h control), SRX1672396 (36 h control), SRX1672397 (48 h control), SRX1672398 (72 h control), SRX1672400 (4 h infection), SRX1672401 (12 h infection), SRX1672402 (24 h infection), SRX1672403 (36 h infection), SRX1672412 (48 h infection), SRX1672413 (72 h infection). The final unigene sequence dataset was annotated via the BLASTX algorithm (http://www.ncbi.nlm.nih.gov/) against Nr (non-redundant protein databases), SwissProt, KEGG (Kyoto Encyclopedia of Genes and Genomes) and COG (Cluster of Orthologous Groups) with a cut-off E-value of 10^−5^ ^53^. Provisional protein functional information was accessed from comparative annotation to the most similar protein in those databases.

### Differential expressed genes analysis

A table of counts constructed with fragments per kb per million fragments (FPKM) values, which adjusted the number of fragments by the total number of fragments mapped and the length of the genes^54^, was used to identify differential expressed CNS genes between control and infected locusts. The FPKM values were calculated under the standard of false discovery rate (FDR) < 0.001 and an absolute value of the log_2_ratio > 1 was considered as significant differential expression. A more stringent screen was performed to analyze the 5-fold differentially expression genes. The software/website: http://revigo.irb.hr/ was employed to analyze the non-redundant and representative GO terms and reveal their interactions. Putative central nervous system related genes or pathways were identified according to the sub-category of nervous system in organismal systems in the KEGG pathway database. In total, these include 10 different pathways, glutamatergic synapse, GABAergic synapse, cholinergic synapse, dopaminergic synapse, serotonergic synapse, long-term potentiation, long-term depression, retrograde endocannabinoid signaling, synaptic vesicle cycle and neurotrophin signaling pathway. Putative immune related genes were preliminarily identified via screening using the BLASTX search algorithm against immune-related family members downloaded from the orthodb database (http://cegg.unige.ch/orthodb7), which included Insecta, waterflea and tick sequences. Searches were parameterized with a cut-off E-value of <10^–5^. Potential immunity-related genes were further analyzed by comparing their protein domains with the deduced protein domains of different family members. Protein domains were determined using Pfam (http://www.sanger.ac.uk/Software/Pfam/) and SMART (http://smart.embl.de/). Manual screening was further performed to verify all putative immune related genes, which were classified into different immune-gene related families.

### qRT-PCR verify hugely differentially expressed CNS genes

A subset of differentially expressed locust CNS genes was examined by qRT-PCR. Primers to each gene are given in Table S4. For analysis, cDNA was reverse-transcribed using Primescript TM RT reagent kit (TaKaRa, China) from total RNA isolated as described above. qRT-PCR was performed using a CFX96TM Real-Time System (Bio-Rad, Hercules, CA, USA) with SYBR green (TaKaRa, China) with the following cycling parameters: 95 °C for 3 min, and 40 cycles of 95 °C for 5 s, 60 °C for 15 s, followed by melting curve generation from 65 to 95 °C. The *actin* gene was used as an internal reference control, and nuclease-free water was used as a negative control. All protocols for qRT-PCR experiments were performed in agreement with the Minimum Information Required for Publication of Quantitative Real-Time PCR Experiments guidelines^55^. Ct values were calculated from the results of two biological replicates. The relative expression levels of each gene was analyzed by 2^−ΔΔCT^ [ΔΔCt = ΔCt(test) − ΔCt (calibrator)] method^56^.

## Electronic supplementary material

Supplementary dataset
