# Supplementary material for: Central Nervous System Responses of the Oriental migratory, Locusta migratoria manilensis, to Fungal Infection
Source: Sci Rep. 2017 Sep 4;7:10340. doi: 10.1038/s41598-017-10622-5 (PMC5583336; doi:10.1038/s41598-017-10622-5)
Supplement: Supplementary file 1 — Supplementary dataset [file 41598_2017_10622_MOESM1_ESM.doc]

**Central Nervous System Responses of the Oriental migratory, *Locusta migratoria manilensis*, to Fungal Infection**

Wei Zhang^1,2^, Jianhong Chen^1^, [Nemat O. Keyhani](http://microcell.ufl.edu/directory/faculty/nemat-o-keyhani/)^1,2^, Kai Jin^1^, Qinglv Wei^1^, Yuxian

**Table S1. 12 locust CNS sequencing results**

| **Samples** | **Total Raw Reads** | **Total Clean Reads** | **Total Clean Nucleotides (nt)** | **Q20 percentage** | **N percentage** | **GC percentage** |
| --- | --- | --- | --- | --- | --- | --- |
| **IFNV1** | 90,350,088 | 80,317,174 | 7,228,545,660 | 97.89% | 0.01% | 38.29% |
| **IFNV2** | 85,065,802 | 77,048,942 | 6,934,404,780 | 97.78% | 0.01% | 40.42% |
| **IFNV3** | 85,615,072 | 76,718,826 | 6,904,694,340 | 97.84% | 0.01% | 38.64% |
| **IFNV4** | 91,096,978 | 81,786,938 | 7,360,824,420 | 97.80% | 0.01% | 38.54% |
| **IFNV5** | 87,531,524 | 77,506,852 | 6,975,616,680 | 97.90% | 0.01% | 38.00% |
| **IFNV6** | 88,327,008 | 78,173,606 | 7,035,624,540 | 97.78% | 0.01% | 39.35% |
| **CKNV1** | 89,505,732 | 82,446,052 | 7,420,144,680 | 97.80% | 0.00% | 38.95% |
| **CKNV2** | 82,880,606 | 76,921,232 | 6,922,910,880 | 97.76% | 0.00% | 40.20% |
| **CKNV3** | 87,274,390 | 80,467,656 | 7,242,089,040 | 97.70% | 0.00% | 39.69% |
| **CKNV4** | 88,009,054 | 80,998,526 | 7,289,867,340 | 97.77% | 0.00% | 39.79% |
| **CKNV5** | 86,069,040 | 78,858,304 | 7,097,247,360 | 97.78% | 0.00% | 39.27% |
| **CKNV6** | 84,028,252 | 77,291,556 | 6,956,240,040 | 97.63% | 0.00% | 41.10% |

Table S2. Combined assembly results

|  | **Total Number** | **Total Length(nt)** | **Mean Length(nt)** | **N50** | **Total Consensus Sequences** | **Distinct Clusters** | **Distinct Singletons** |
| --- | --- | --- | --- | --- | --- | --- | --- |
| **Contig** | 323,680 | 108,877,856 | 336 | 680 | - | - | - |
| **Unigene** | 171,566 | 129,987,660 | 758 | 1791 | 171,566 | 46,397 | 125,169 |
| **All** | 128,988 | 118,641,789 | 920 | 1798 | 128,988 | 38,189 | 90,799 |

**Table S3. Significantly enriched cell component for different pi time points**

Bold terms represent specifically significantly enriched components in corresponding infection stages.

| **pi Time** | **Gene Ontology term** | **Cluster frequency** | **Genome frequency of use** | **Corrected P-value** |
| --- | --- | --- | --- | --- |
| **4h** | cell periphery | 189/831, 0.23 | 1210/8084, 0.15 | 6.71E-08 |
|  | intrinsic to membrane | 204/831, 0.25 | 1362/8084, 0.17 | 4.65E-07 |
|  | integral to membrane | 202/831, 0.24 | 1348/8084, 0.17 | 5.56E-07 |
|  | membrane | 390/831, 0.47 | 3063/8084, 0.38 | 4.17E-06 |
|  | plasma membrane | 166/831, 0.20 | 1101/8084, 0.14 | 1.66E-05 |
|  | cell junction | 67/831, 0.08 | 346/8084, 0.04 | 5.49E-05 |
|  | membrane part | 253/831, 0.03 | 1870/8084, 0.23 | 5.91E-05 |
|  | synapse | 71/831, 0.09 | 376/8084, 0.05 | 6.48E-05 |
|  | **coated pit** | 11/831, 0.01 | 19/8084, 0.00 | 0.00014 |
|  | synapse part | 53/831, 0.06 | 269/8084, 0.03 | 0.00065 |
|  | **clathrin-coated vesicle** | 27/831, 0.03 | 105/8084, 0.01 | 0.00161 |
|  | **nuclear pore** | 12/831, 0.01 | 27/8084, 0.00 | 0.00175 |
|  | **pore complex** | 12/831, 0.01 | 27/8084, 0.00 | 0.00175 |
|  | **pleated septate junction** | 5/831, 0.01 | 5/8084, 0.00 | 0.00384 |
|  | **synaptic vesicle** | 21/831, 0.03 | 81/8084, 0.01 | 0.01592 |
|  | **coated vesicle** | 27/831, 0.03 | 120/8084, 0.01 | 0.02171 |
|  | synaptic membrane | 27/831, 0.03 | 120/8084, 0.01 | 0.02171 |
|  | astral microtubule | 8/831, 0.01 | 16/8084, 0.00 | 0.02477 |
|  | aster | 8/831, 0.01 | 16/8084, 0.00 | 0.02477 |
|  | **presynaptic membrane** | 12/831, 0.01 | 34/8084, 0.00 | 0.02805 |
|  | cell-cell junction | 39/831, 0.05 | 204/8084, 0.03 | 0.02982 |
|  | plasma membrane part | 90/831, 0.11 | 605/8084, 0.07 | 0.04909 |
| **12h** | cell periphery | 177/758, 0.23 | 1210/8084, 0.15 | 2.52E-08 |
|  | plasma membrane | 156/758, 0.21 | 1101/8084, 0.14 | 4.87E-06 |
|  | integral to plasma membrane | 63/758, 0.08 | 332/8084, 0.04 | 8.05E-06 |
|  | integral to membrane | 180/758, 0.24 | 1348/8084, 0.17 | 2.65E-05 |
|  | intrinsic to plasma membrane | 63/758, 0.08 | 343/8084, 0.04 | 2.85E-05 |
|  | plasma membrane part | 95/758, 0.13 | 605/8084, 0.07 | 4.92E-05 |
|  | intrinsic to membrane | 180/758, 0.24 | 1362/8084, 0.17 | 5.66E-05 |
|  | **spectrin** | 8/758, 0.01 | 10/8084, 0.00 | 6.32E-05 |
|  | astral microtubule | 10/758, 0.01 | 16/8084, 0.00 | 6.80E-05 |
|  | aster | 10/758, 0.01 | 16/8084, 0.00 | 6.80E-05 |
|  | rhabdomere | 14/758, | 33/8084, 0.00 | 0.00015 |
|  | **cortical actin cytoskeleton** | 8/758, 0.01 | 12/8084, 0.00 | 0.00058 |
|  | **cortical cytoskeleton** | 10/758, 0.01 | 20/8084, 0.00 | 0.0011 |
|  | **perinuclear region of cytoplasm** | 27/758, 0.04 | 120/8084, 0.01 | 0.00358 |
|  | cell junction | 57/758, 0.08 | 346/8084, 0.04 | 0.00409 |
|  | membrane part | 222/758, 0.29 | 1870/8084, 0.23 | 0.00646 |
|  | synapse | 59/758, 0.08 | 376/8084, 0.05 | 0.01277 |
|  | neuron part | 56/758, 0.07 | 359/8084, 0.04 | 0.02361 |
|  | **cytoplasmic microtubule** | 11/758, 0.01 | 32/8084, 0.00 | 0.02703 |
| **24h** | cell periphery | 247/1059, 0.23 | 1210/8084, 0.15 | 2.22E-12 |
|  | plasma membrane | 228/1059, 0.22 | 1101/8084, 0.14 | 6.82E-12 |
|  | integral to membrane | 258/1059, 0.24 | 1348/8084, 0.17 | 1.32E-09 |
|  | intrinsic to membrane | 260/1059, 0.25 | 1362/8084, 0.17 | 1.43E-09 |
|  | cell junction | 88/1059, 0.08 | 346/8084, 0.04 | 7.16E-08 |
|  | membrane | 490/1059, 0.46 | 3063/8084, 0.38 | 5.35E-07 |
|  | membrane part | 323/1059, 0.31 | 1870/8084, 0.23 | 6.70E-07 |
|  | plasma membrane part | 126/1059, 0.12 | 605/8084, 0.07 | 9.27E-06 |
|  | synapse | 85/1059, 0.08 | 376/8084, 0.05 | 5.62E-05 |
|  | synapse part | 64/1059, 0.06 | 269/8084, 0.03 | 0.00031 |
|  | integral to plasma membrane | 74/1059, 0.07 | 332/8084, 0.04 | 0.00065 |
|  | intrinsic to plasma membrane | 75/1059, 0.07 | 343/8084, 0.04 | 0.00119 |
|  | synaptic membrane | 33/1059, 0.03 | 120/8084, 0.01 | 0.00688 |
|  | cell-cell junction | 48/1059, 0.05 | 204/8084, 0.03 | 0.01027 |
|  | **anchoring junction** | 29/1059, 0.03 | 102/8084, 0.01 | 0.01111 |
|  | postsynaptic membrane | 27/1059, 0.03 | 94/8084, 0.01 | 0.01702 |
|  | astral microtubule | 9/1059, 0.01 | 16/8084, 0.00 | 0.01992 |
|  | aster | 9/1059, 0.01 | 16/8084, 0.00 | 0.01992 |
|  | basolateral plasma membrane | 23/1059, 0.02 | 75/8084, 0.01 | 0.0201 |
|  | neuron part | 73/1059, 0.07 | 359/8084, 0.04 | 0.02221 |
|  | **adherens junction** | 28/1059, 0.03 | 101/8084, 0.01 | 0.02491 |
| **36h** |  |  |  |  |
| **48h** | membrane | 370/796, 0.46 | 3063/8084, 0.38 | 3.54E-05 |
|  | intrinsic to membrane | 187/796, 0.23 | 1362/8084, 0.17 | 7.59E-05 |
|  | integral to membrane | 185/796, 0.23 | 1348/8084, 0.17 | 9.32E-05 |
|  | cell periphery | 165/796, 0.21 | 1210/8084, 0.15 | 0.00076 |
|  | **M band** | 10/796, 0.01 | 19/8084, 0.00 | 0.001 |
|  | plasma membrane | 152/796, 0.19 | 1101/8084, 0.14 | 0.00103 |
|  | **spindle** | 26/796, 0.03 | 104/8084, 0.01 | 0.00174 |
| **72h** | cell periphery | 338/1456, 0.23 | 1210/8084, 0.15 | 3.99E-18 |
|  | plasma membrane | 310/1456, 0.21 | 1101/8084, 0.14 | 7.64E-17 |
|  | integral to membrane | 363/1456, 0.25 | 1348/8084, 0.17 | 8.96E-17 |
|  | intrinsic to membrane | 364/1456, 0.25 | 1362/8084, 0.17 | 3.17E-16 |
|  | membrane | 700/1456, 0.48 | 3063/8084, 0.38 | 4.76E-16 |
|  | membrane part | 441/1456, 0.30 | 1870/8084, 0.23 | 6.83E-10 |
|  | cell junction | 108/1456, 0.07 | 346/8084, 0.04 | 3.00E-07 |
|  | integral to plasma membrane | 100/1456, 0.07 | 332/8084, 0.04 | 1.03E-05 |
|  | plasma membrane part | 161/1456, 0.11 | 605/8084, 0.07 | 1.15E-05 |
|  | synapse | 110/1456, 0.08 | 376/8084, 0.05 | 1.17E-05 |
|  | synaptic membrane | 47/1456, 0.03 | 120/8084, 0.01 | 1.28E-05 |
|  | intrinsic to plasma membrane | 100/1456, 0.07 | 343/8084, 0.04 | 6.16E-05 |
|  | postsynaptic membrane | 38/1456, 0.03 | 94/8084, 0.01 | 0.0001 |
|  | synapse part | 80/1456, 0.05 | 269/8084, 0.03 | 0.00046 |
|  | **external encapsulating structure** | 19/1456, 0.01 | 35/8084, 0.00 | 0.00051 |
|  | **outer membrane-****bounded periplasmic space** | 15/1456, 0.01 | 26/8084, 0.00 | 0.00251 |
|  | **cell envelope** | 15/1456, 0.01 | 26/8084, 0.00 | 0.00251 |
|  | **periplasmic space** | 15/1456, 0.01 | 26/8084, 0.00 | 0.00251 |
|  | **external encapsulating structure part** | 15/1456, 0.01 | 26/8084, 0.00 | 0.00251 |
|  | neuron part | 96/1456, 0.07 | 359/8084, 0.04 | 0.00669 |
|  | basolateral plasma membrane | 29/1456, 0.02 | 75/8084, 0.01 | 0.00719 |
|  | **smooth endoplasmic reticulum** | 10/1456, 0.01 | 15/8084, 0.00 | 0.01635 |
|  | rhabdomere | 16/1456, 0.01 | 33/8084, 0.00 | 0.02252 |
|  | astral microtubule | 10/1456, 0.01 | 16/8084, 0.00 | 0.03656 |
|  | aster | 10/1456, 0.01 | 16/8084, 0.00 | 0.03656 |

**Table S4. Significantly enriched Molecular function for different pi time points**

Bold terms represent specifically significantly enriched components in corresponding infection stages.

| **pi Time** | **Gene Ontology term** | **Cluster frequency** | **Genome frequency of use** | **Corrected P-value** |
| --- | --- | --- | --- | --- |
| **4h** | lipid binding | 53/1189, 0.04 | 240/11915, 0.02 | 1.00E-05 |
|  | phospholipid binding | 43/1189, 0.04 | 176/11915, 0.01 | 1.02E-05 |
|  | **clathrin binding** | 9/1189, 0.01 | 11/11915, 0.00 | 2.37E-05 |
|  | protein serine/threonine kinase activity | 73/1189, 0.06 | 388/11915, 0.03 | 3.17E-05 |
|  | protein kinase activity | 105/1189, 0.09 | 645/11915, 0.05 | 0.00011 |
|  | phosphotransferase activity, alcohol group as acceptor | 120/1189, 0.10 | 767/11915, 0.06 | 0.00013 |
|  | **1-phosphatidylinositol binding** | 8/1189, 0.01 | 11/11915, 0.00 | 0.00065 |
|  | kinase activity | 134/1189, 0.11 | 916/11915, 0.08 | 0.00121 |
|  | calmodulin binding | 18/1189, 0.02 | 55/11915, 0.00 | 0.00182 |
|  | **ATP binding** | 129/1189, 0.11 | 897/11915, 0.08 | 0.00459 |
|  | **ribonucleoside binding** | 160/1189, 0.13 | 1167/11915, 0.10 | 0.0058 |
|  | **adenyl ribonucleotide binding** | 129/1189, 0.11 | 906/11915, 0.08 | 0.00755 |
|  | **adenyl nucleotide binding** | 129/1189, 0.11 | 907/11915, 0.08 | 0.00797 |
|  | **nucleoside binding** | 160/1189, 0.13 | 1175/11915, 0.10 | 0.00853 |
|  | **anion binding** | 183/1189, 0.15 | 1378/11915, 0.12 | 0.00902 |
|  | **purine ribonucleoside triphosphate binding** | 158/1189, 0.13 | 1159/11915, 0.10 | 0.00913 |
|  | **purine ribonucleoside binding** | 158/1189, 0.13 | 1164/11915, 0.10 | 0.01157 |
|  | **purine ribonucleotide binding** | 158/1189, 0.13 | 1167/11915, 0.10 | 0.01332 |
|  | **ribonucleotide binding** | 159/1189, 0.13 | 1176/11915, 0.10 | 0.01348 |
|  | **purine nucleotide binding** | 158/1189, 0.13 | 1170/11915, 0.10 | 0.01531 |
|  | **transferase activity, transferring phosphorus-containing groups** | 159/1189, 0.13 | 1179/11915, 0.10 | 0.01549 |
|  | **purine nucleoside binding** | 158/1189, 0.13 | 1172/11915, 0.10 | 0.01678 |
|  | **aminopeptidase activity** | 10/1189, 0.01 | 23/11915, 0.00 | 0.01738 |
|  | **protein binding** | 291/1189, 0.24 | 2395/11915, 0.20 | 0.03226 |
| **12h** | calcium ion transmembrane transporter activity | 26/1139, 0.02 | 81/11915, 0.01 | 7.73E-06 |
|  | calcium ion binding | 60/1139, 0.05 | 322/11915, 0.03 | 0.00014 |
|  | **divalent inorganic cation transmembrane transporter activity** | 26/1139, 0.02 | 95/11915, 0.01 | 0.00027 |
|  | lipid binding | 46/1139, 0.04 | 240/11915, 0.02 | 0.00164 |
|  | phospholipid binding | 36/1139, 0.03 | 176/11915, 0.01 | 0.00426 |
|  | **beta-catenin binding** | 13/1139, 0.01 | 35/11915, 0.00 | 0.00527 |
|  | **cadherin binding** | 15/1139, 0.01 | 46/11915, 0.00 | 0.00701 |
|  | receptor activity | 79/1139, 0.07 | 517/11915, 0.04 | 0.00798 |
|  | metal ion transmembrane transporter activity | 51/1139, 0.04 | 295/11915, 0.02 | 0.01035 |
|  | **microtubule binding** | 19/1139, 0.02 | 70/11915, 0.01 | 0.01037 |
|  | **calcium channel activity** | 16/1139, 0.01 | 54/11915, 0.00 | 0.01418 |
|  | **protein tyrosine phosphatase activity** | 17/1139, 0.01 | 61/11915, 0.01 | 0.01935 |
|  | **calcium-transporting ATPase activity** | 9/1139, 0.01 | 20/11915, 0.00 | 0.02091 |
|  | **cation binding** | 183/1139, 0.16 | 1471/11915, 0.12 | 0.03117 |
|  | **uridine phosphorylase activity** | 4/1139, 0.00 | 4/11915, 0.00 | 0.04246 |
|  | **intracellular ligand-gated calcium channel activity** | 4/1139, 0.00 | 4/11915, 0.00 | 0.04246 |
| **24h** | lipid binding | 65/1572, 0.04 | 240/11915, 0.02 | 3.47E-06 |
|  | receptor activity | 112/1572, 0.07 | 517/11915, 0.04 | 2.32E-05 |
|  | phospholipid binding | 48/1572, 0.03 | 176/11915, 0.01 | 0.00027 |
|  | phosphotransferase activity, alcohol group as acceptor | 147/1572, 0.09 | 767/11915, 0.06 | 0.00059 |
|  | signal transducer activity | 116/1572, 0.07 | 575/11915, 0.05 | 0.00068 |
|  | molecular transducer activity | 116/1572, 0.07 | 575/11915, 0.05 | 0.00068 |
|  | protein kinase activity | 123/1572, 0.08 | 645/11915, 0.05 | 0.00611 |
|  | signaling receptor activity | 89/1572, 0.06 | 436/11915, 0.04 | 0.00768 |
|  | kinase activity | 163/1572, 0.10 | 916/11915, 0.08 | 0.01398 |
|  | calcium ion binding | 69/1572, 0.04 | 322/11915, 0.03 | 0.01444 |
|  | calcium ion transmembrane transporter activity | 25/1572, 0.02 | 81/11915, 0.01 | 0.01632 |
|  | transmembrane signaling receptor activity | 77/1572, 0.05 | 373/11915, 0.03 | 0.01966 |
|  | GTPase regulator activity | 46/1572, 0.03 | 197/11915, 0.02 | 0.03866 |
| **36h** | inositol oxygenase activity | 7/591, 0.01 | 14/11915, 0.00 | 0.0006 |
|  | **ryanodine-sensitive calcium-release channel activity** | 3/591, 0.01 | 3/11915, 0.00 | 0.04104 |
| **48h** | **aldehyde-lyase activity** | 10/1285, 0.01 | 14/11915, 0.00 | 7.80E-05 |
|  | phosphotransferase activity, alcohol group as acceptor | 127/1285, 0.10 | 767/11915, 0.06 | 0.00019 |
|  | **fructose-bisphosphate aldolase activity** | 8/1285, 0.01 | 10/11915, 0.00 | 0.00037 |
|  | protein kinase activity | 109/1285, 0.08 | 645/11915, 0.05 | 0.0005 |
|  | inositol oxygenase activity | 9/1285, 0.01 | 14/11915, 0.00 | 0.00132 |
|  | kinase activity | 141/1285, 0.11 | 916/11915, 0.08 | 0.00289 |
|  | **platelet-derived growth factor-activated receptor activity** | 5/1285, 0.00 | 5/11915, 0.00 | 0.00824 |
|  | **vascular endothelial growth factor-activated receptor activity** | 5/1285, 0.00 | 5/11915, 0.00 | 0.00824 |
|  | enzyme regulator activity | 65/1285, 0.05 | 362/11915, 0.03 | 0.0128 |
|  | transmembrane receptor protein kinase activity | 14/1285, 0.01 | 41/11915, 0.00 | 0.0324 |
| **72h** | receptor activity | 168/2203, 0.08 | 517/11915, 0.04 | 3.21E-12 |
|  | signal transducer activity | 177/2203, 0.08 | 575/11915, 0.05 | 1.54E-10 |
|  | molecular transducer activity | 177/2203, 0.08 | 575/11915, 0.05 | 1.54E-10 |
|  | signaling receptor activity | 142/2203, 0.06 | 436/11915, 0.04 | 3.95E-10 |
|  | transmembrane signaling receptor activity | 125/2203, 0.06 | 373/11915, 0.03 | 1.13E-09 |
|  | phosphotransferase activity, alcohol group as acceptor | 208/2203, 0.09 | 767/11915, 0.06 | 6.26E-07 |
|  | protein kinase activity | 178/2203, 0.08 | 645/11915, 0.05 | 2.79E-06 |
|  | transmembrane receptor protein kinase activity | 24/2203, 0.01 | 41/11915, 0.00 | 1.03E-05 |
|  | protein serine/threonine kinase activity | 116/2203, 0.05 | 388/11915, 0.03 | 1.64E-05 |
|  | kinase activity | 231/2203, 0.10 | 916/11915, 0.08 | 6.86E-05 |
|  | **extracellular-glutamate-gated ion channel activity** | 18/2203, 0.01 | 29/11915, 0.00 | 0.0002 |
|  | **excitatory extracellular ligand-gated ion channel activity** | 31/2203, 0.01 | 71/11915, 0.01 | 0.00067 |
|  | **transmembrane receptor protein tyrosine kinase activity** | 19/2203, 0.01 | 36/11915, 0.00 | 0.00293 |
|  | calmodulin binding | 25/2203, 0.01 | 55/11915, 0.00 | 0.00315 |
|  | metal ion transmembrane transporter activity | 86/2203, 0.04 | 295/11915, 0.02 | 0.00341 |
|  | **glutamate receptor activity** | 20/2203, 0.01 | 40/11915, 0.00 | 0.00483 |
|  | **ligand-gated ion channel activity** | 45/2203, 0.02 | 129/11915, 0.01 | 0.00526 |
|  | **ligand-gated channel activity** | 45/2203, 0.02 | 129/11915, 0.01 | 0.00526 |
|  | calcium ion transmembrane transporter activity | 32/2203, 0.01 | 81/11915, 0.01 | 0.00611 |
|  | **alpha-amino-3-hydroxy-5-methyl-4-isoxazole propionate selective glutamate receptor activity** | 8/2203, 0.00 | 9/11915, 0.00 | 0.00803 |
|  | **ion channel activity** | 87/2203, 0.04 | 306/11915, 0.03 | 0.00888 |
|  | **transporter activity** | 245/2203, 0.11 | 1041/11915, 0.09 | 0.00909 |
|  | **substrate-specific transporter activity** | 197/2203, 0.09 | 811/11915, 0.07 | 0.00929 |
|  | **substrate-specific channel activity** | 87/2203, 0.04 | 307/11915, 0.03 | 0.01019 |
|  | **extracellular ligand-gated ion channel activity** | 35/2203, 0.02 | 94/11915, 0.01 | 0.01097 |
|  | **channel activity** | 88/2203, 0.04 | 313/11915, 0.03 | 0.01271 |
|  | **passive transmembrane transporter activity** | 88/2203, 0.04 | 313/11915, 0.03 | 0.01271 |
|  | inositol oxygenase activity | 10/2203, 0.00 | 14/11915, 0.00 | 0.01747 |
|  | GTPase regulator activity | 60/2203, 0.03 | 197/11915, 0.02 | 0.02291 |
|  | enzyme regulator activity | 98/2203, 0.04 | 362/11915, 0.03 | 0.02327 |
|  | **protein tyrosine kinase activity** | 35/2203, 0.02 | 97/11915, 0.01 | 0.02395 |
|  | lipid binding | 70/2203, 0.03 | 240/11915, 0.02 | 0.02548 |
|  | **gated channel activity** | 73/2203, 0.03 | 253/11915, 0.02 | 0.0259 |
|  | **ion gated channel activity** | 73/2203, 0.03 | 253/11915, 0.02 | 0.0259 |
|  | **substrate-specific transmembrane transporter activity** | 181/2203, 0.08 | 748/11915, 0.06 | 0.02699 |
|  | **monoamine transmembrane transporter activity** | 7/2203, 0.00 | 8/11915, 0.00 | 0.03884 |
|  | **ionotropic glutamate receptor activity** | 16/2203, 0.01 | 32/11915, 0.00 | 0.04131 |
|  | **nucleoside-triphosphatase regulator activity** | 60/2203, 0.03 | 201/11915, 0.02 | 0.04395 |
|  | phospholipid binding | /2203, 0.02 | 176/11915, 0.01 | 0.04517 |

**Table S5. Significantly enriched Biological process for different pi time points**

Bold terms represent specifically significantly enriched components in corresponding infection stages.

| **pi Time** | **Gene Ontology term** | **Cluster frequency** | **Genome frequency of use** | **Corrected P-value** |
| --- | --- | --- | --- | --- |
| **4h** | cell communication | 309/1157, 0.27 | 1894/11234, 0.17 | 5.69E-16 |
|  | signaling | 295/1157, 0.25 | 1813/11234, 0.16 | 8.03E-15 |
|  | single organism signaling | 295/1157, 0.25 | 1813/11234, 0.16 | 8.03E-15 |
|  | transmission of nerve impulse | 99/1157, 0.09 | 454/11234, 0.04 | 3.31E-10 |
|  | multicellular organismal signaling | 99/1157, 0.09 | 455/11234, 0.04 | 3.82E-10 |
|  | system process | 162/1157, 0.14 | 903/11234, 0.08 | 4.78E-10 |
|  | biological regulation | 531/1157, 0.46 | 4060/11234, 0.36 | 8.35E-10 |
|  | synaptic transmission | 93/1157, 0.08 | 426/11234, 0.04 | 1.76E-09 |
|  | regulation of cellular process | 462/1157, 0.40 | 3480/11234, 0.31 | 1.19E-08 |
|  | regulation of biological process | 494/1157, 0.43 | 3779/11234, 0.31 | 1.62E-08 |
|  | cell-cell signaling | 123/1157, 0.11 | 653/11234, 0.06 | 1.92E-08 |
|  | response to stimulus | 407/1157, 0.35 | 3019/11234, 0.27 | 6.81E-08 |
|  | neurological system process | 145/1157, 0.13 | 835/11234, 0.07 | 1.28E-07 |
|  | signal transduction | 194/1157, 0.17 | 1258/11234, 0.11 | 2.46E-06 |
|  | regulation of neurotransmitter levels | 36/1157, 0.03 | 124/11234, 0.01 | 9.89E-06 |
|  | neurotransmitter transport | 39/1157, 0.03 | 141/11234, 0.01 | 1.04E-05 |
|  | synapse organization | 39/1157, 0.03 | 141/11234, 0.01 | 1.04E-05 |
|  | cell development | 213/1157, 0.18 | 1438/11234, 0.13 | 1.14E-05 |
|  | **neurotransmitter secretion** | 34/1157, 0.03 | 114/11234, 0.01 | 1.23E-05 |
|  | energy taxis | 14/1157, 0.01 | 24/11234, 0.00 | 2.15E-05 |
|  | phototaxis | 14/1157, 0.01 | 24/11234, 0.00 | 2.15E-05 |
|  | **generation of a signal involved in cell-cell signaling** | 34/1157, 0.03 | 119/11234, 0.01 | 4.12E-05 |
|  | **signal release** | 34/1157, 0.03 | 119/11234, 0.01 | 4.12E-05 |
|  | single-multicellular organism process | 436/1157, 0.38 | 3438/11234, 0.31 | 7.16E-05 |
|  | single-organism process | 762/1157, 0.66 | 6571/11234, 0.58 | 7.54E-05 |
|  | single-organism cellular process | 652/1157, 0.56 | 5513/11234, 0.49 | 0.0002 |
|  | regulation of neuron differentiation | 37/1157, 0.03 | 145/11234, 0.01 | 0.00025 |
|  | multicellular organismal process | 449/1157, 0.39 | 3596/11234, 0.32 | 0.0003 |
|  | single-organism developmental process | 348/1157, 0.30 | 2676/11234, 0.24 | 0.00031 |
|  | skeletal muscle organ development | 43/1157, 0.04 | 186/11234, 0.02 | 0.00052 |
|  | regulation of anatomical structure morphogenesis | 64/1157, 0.06 | 327/11234, 0.03 | 0.00056 |
|  | synaptic vesicle transport | 31/1157, 0.03 | 114/11234, 0.01 | 0.00059 |
|  | regulation of axon extension | 20/1157, 0.02 | 56/11234, 0.00 | 0.00067 |
|  | regulation of extent of cell growth | 20/1157, 0.02 | 56/11234, 0.00 | 0.00067 |
|  | regulation of cell communication | 121/1157, 0.10 | 756/11234, 0.07 | 0.00069 |
|  | regulation of signaling | 121/1157, 0.10 | 756/11234, 0.07 | 0.00069 |
|  | intracellular signal transduction | 81/1157, 0.07 | 451/11234, 0.04 | 0.00069 |
|  | **secretion by cell** | 44/1157, 0.04 | 195/11234, 0.02 | 0.00079 |
|  | secretion | 45/1157, 0.04 | 202/11234, 0.02 | 0.00086 |
|  | regulation of neurogenesis | 42/1157, 0.04 | 184/11234, 0.02 | 0.00104 |
|  | regulation of cell morphogenesis | 53/1157, 0.05 | 257/11234, 0.02 | 0.00113 |
|  | regulation of synaptic transmission | 27/1157, 0.02 | 95/11234, 0.01 | 0.00132 |
|  | anatomical structure morphogenesis | 256/1157, 0.22 | 1910/11234, 0.17 | 0.00255 |
|  | regulation of transmission of nerve impulse | 27/1157, 0.02 | 98/11234, 0.01 | 0.00259 |
|  | cellular developmental process | 278/1157, 0.24 | 2107/11234, 0.19 | 0.00299 |
|  | learning | 25/1157, 0.02 | 88/11234, 0.01 | 0.00345 |
|  | regulation of neurological system process | 27/1157, 0.02 | 100/11234, 0.01 | 0.00399 |
|  | regulation of neuron projection development | 30/1157, 0.03 | 118/11234, 0.01 | 0.00431 |
|  | regulation of response to stimulus | 132/1157, 0.11 | 872/11234, 0.08 | 0.00457 |
|  | cellular component maintenance | 16/1157, 0.01 | 43/11234, 0.00 | 0.00547 |
|  | regulation of axonogenesis | 25/1157, 0.02 | 91/11234, 0.01 | 0.00676 |
|  | anatomical structure development | 374/1157, 0.32 | 2994/11234, 0.27 | 0.00729 |
|  | cell differentiation | 270/1157, 0.23 | 2064/11234, 0.18 | 0.00927 |
|  | **short-term memory** | 10/1157, 0.01 | 19/11234, 0.00 | 0.01055 |
|  | system development | 321/1157, 0.28 | 2526/11234, 0.22 | 0.01174 |
|  | **conditioned taste aversion** | 6/1157, 0.01 | 7/11234, 0.00 | 0.01592 |
|  | **clathrin coat assembly** | 6/1157, 0.01 | 7/11234, 0.00 | 0.01592 |
|  | regulation of secretion | 20/1157, 0.02 | 67/11234, 0.01 | 0.01647 |
|  | muscle organ development | 52/1157, 0.04 | 273/11234, 0.02 | 0.01694 |
|  | protein phosphorylation | 73/1157, 0.06 | 427/11234, 0.04 | 0.0171 |
|  | **regulation of tube architecture, open tracheal system** | 26/1157, 0.02 | 102/11234, 0.01 | 0.01941 |
|  | organ development | 226/1157, 0.20 | 1696/11234, 0.15 | 0.02002 |
|  | **nerve maturation** | 5/1157, 0.00 | 5/11234, 0.00 | 0.0243 |
|  | regulation of nervous system development | 54/1157, 0.05 | 291/11234, 0.03 | 0.02509 |
|  | regulation of cell morphogenesis involved in differentiation | 30/1157, 0.03 | 128/11234, 0.01 | 0.0253 |
|  | developmental process | 402/1157, 0.35 | 3290/11234, 0.29 | 0.02625 |
|  | generation of neurons | 156/1157, 0.13 | 1103/11234, 0.10 | 0.0284 |
|  | **courtship behavior** | 22/1157, 0.02 | 81/11234, 0.01 | 0.03174 |
|  | cellular component organization | 312/1157, 0.27 | 2475/11234, 0.22 | 0.03445 |
|  | regulation of biological quality | 154/1157, 0.13 | 1091/11234, 0.10 | 0.0362 |
|  | regulation of cell development | 61/1157, 0.05 | 346/11234, 0.03 | 0.03659 |
|  | **intracellular protein kinase cascade** | 19/1157, 0.02 | 65/11234, 0.01 | 0.03862 |
|  | neuron development | 130/1157, 0.11 | 893/11234, 0.08 | 0.04064 |
|  | cellular response to stimulus | 225/1157, 0.19 | 1709/11234, 0.15 | 0.04881 |
| **12h** | cell communication | 283/1119, 0.25 | 1894/11234, 0.17 | 5.46E-11 |
|  | regulation of cellular process | 452/1119, 0.40 | 3480/11234, 0.31 | 2.63E-09 |
|  | signaling | 264/1119, 0.24 | 1813/11234, 0.16 | 1.21E-08 |
|  | single organism signaling | 264/1119, 0.24 | 1813/11234, 0.16 | 1.21E-08 |
|  | neuron development | 152/1119, 0.14 | 893/11234, 0.08 | 1.43E-08 |
|  | biological regulation | 509/1119, 0.45 | 4060/11234, 0.36 | 1.67E-08 |
|  | single-organism cellular process | 656/1119, 0.59 | 5513/11234, 0.49 | 1.97E-08 |
|  | muscle organ development | 65/1119, 0.06 | 273/11234, 0.02 | 2.67E-08 |
|  | cell development | 216/1119, 0.19 | 1438/11234, 0.13 | 1.04E-07 |
|  | **neuron differentiation** | 163/1119, 0.15 | 1005/11234, 0.09 | 1.21E-07 |
|  | regulation of biological process | 475/1119, 0.42 | 3779/11234, 0.34 | 1.25E-07 |
|  | regulation of neuron differentiation | 42/1119, 0.04 | 145/11234, 0.01 | 1.92E-07 |
|  | regulation of neuron projection development | 37/1119, 0.03 | 118/11234, 0.01 | 2.05E-07 |
|  | generation of neurons | 174/1119, 0.16 | 1103/11234, 0.10 | 2.49E-07 |
|  | regulation of axon extension | 24/1119, 0.02 | 56/11234, 0.00 | 2.84E-07 |
|  | regulation of extent of cell growth | 24/1119, 0.02 | 56/11234, 0.00 | 2.84E-07 |
|  | regulation of axonogenesis | 31/1119, 0.03 | 91/11234, 0.01 | 6.68E-07 |
|  | **cellular homeostasis** | 56/1119, 0.05 | 238/11234, 0.02 | 1.16E-06 |
|  | single-organism process | 748/1119, 0.67 | 6571/11234, 0.58 | 1.94E-06 |
|  | regulation of cell morphogenesis involved in differentiation | 37/1119, 0.03 | 128/11234, 0.01 | 2.69E-06 |
|  | regulation of cell projection organization | 45/1119, 0.04 | 175/11234, 0.02 | 2.99E-06 |
|  | synapse organization | 39/1119, 0.03 | 141/11234, 0.01 | 3.96E-06 |
|  | **axonogenesis** | 98/1119, 0.09 | 541/11234, 0.05 | 4.18E-06 |
|  | **regulation of response to external stimulus** | 30/1119, 0.03 | 92/11234, 0.01 | 4.27E-06 |
|  | response to stimulus | 385/1119, 0.34 | 3019/11234, 0.27 | 5.97E-06 |
|  | system process | 144/1119, 0.13 | 903/11234, 0.08 | 6.22E-06 |
|  | **homeostatic process** | 72/1119, 0.06 | 357/11234, 0.03 | 6.58E-06 |
|  | nervous system development | 233/1119, 0.21 | 1658/11234, 0.15 | 1.00E-05 |
|  | regulation of biological quality | 166/1119, 0.15 | 1091/11234, 0.10 | 1.14E-05 |
|  | regulation of cell communication | 124/1119, 0.11 | 756/11234, 0.07 | 1.65E-05 |
|  | regulation of signaling | 124/1119, 0.11 | 756/11234, 0.07 | 1.65E-05 |
|  | skeletal muscle organ development | 45/1119, 0.04 | 186/11234, 0.02 | 2.34E-05 |
|  | **regulation of G-protein coupled receptor protein signaling pathway** | 17/1119, 0.02 | 36/11234, 0.00 | 2.37E-05 |
|  | **regulation of female receptivity** | 14/1119, 0.01 | 25/11234, 0.00 | 2.83E-05 |
|  | neurological system process | 133/1119, 0.12 | 835/11234, 0.07 | 2.92E-05 |
|  | single-organism developmental process | 344/1119, 0.31 | 2676/11234, 0.24 | 2.97E-05 |
|  | **muscle structure development** | 75/1119, 0.07 | 392/11234, 0.03 | 3.39E-05 |
|  | regulation of synaptic transmission | 29/1119, 0.03 | 95/11234, 0.01 | 4.28E-05 |
|  | mating | 34/1119, 0.03 | 123/11234, 0.01 | 4.38E-05 |
|  | response to external stimulus | 115/1119, 0.10 | 698/11234, 0.06 | 4.72E-05 |
|  | **muscle cell homeostasis** | 21/1119, 0.02 | 55/11234, 0.00 | 4.88E-05 |
|  | regulation of neurogenesis | 44/1119, 0.04 | 184/11234, 0.02 | 4.96E-05 |
|  | single-multicellular organism process | 424/1119, 0.38 | 3438/11234, 0.31 | 5.25E-05 |
|  | **rhabdomere development** | 22/1119, 0.02 | 60/11234, 0.01 | 5.41E-05 |
|  | calcium ion transmembrane transport | 18/1119, 0.02 | 42/11234, 0.00 | 5.86E-05 |
|  | neuromuscular junction development | 27/1119, 0.02 | 87/11234, 0.01 | 8.91E-05 |
|  | regulation of transmission of nerve impulse | 29/1119, 0.03 | 98/11234, 0.01 | 9.26E-05 |
|  | taxis | 90/1119, 0.08 | 515/11234, 0.05 | 0.00011 |
|  | multicellular organismal process | 438/1119, 0.39 | 3596/11234, 0.32 | 0.00013 |
|  | cell differentiation | 273/1119, 0.24 | 2064/11234, 0.18 | 0.00013 |
|  | **cell morphogenesis involved in neuron differentiation** | 106/1119, 0.09 | 641/11234, 0.06 | 0.00014 |
|  | **axon target recognition** | 21/1119, 0.02 | 58/11234, 0.01 | 0.00014 |
|  | regulation of neurological system process | 29/1119, 0.03 | 100/11234, 0.01 | 0.00015 |
|  | regulation of cell morphogenesis | 54/1119, 0.05 | 257/11234, 0.02 | 0.00015 |
|  | system development | 323/1119, 0.29 | 2526/11234, 0.22 | 0.00019 |
|  | skeletal muscle fiber development | 27/1119, 0.02 | 90/11234, 0.01 | 0.00019 |
|  | transmission of nerve impulse | 81/1119, 0.07 | 454/11234, 0.04 | 0.00022 |
|  | multicellular organismal signaling | 81/1119, 0.07 | 455/11234, 0.04 | 0.00024 |
|  | synaptic transmission | 77/1119, 0.07 | 426/11234, 0.04 | 0.00026 |
|  | **axon guidance** | 79/1119, 0.07 | 441/11234, 0.04 | 0.00027 |
|  | **chemotaxis** | 81/1119, 0.07 | 457/11234, 0.04 | 0.00029 |
|  | **cell morphogenesis involved in differentiation** | 110/1119, 0.10 | 683/11234, 0.06 | 0.00033 |
|  | cellular component organization | 316/1119, 0.28 | 2475/11234, 0.22 | 0.00033 |
|  | mating behavior | 31/1119, 0.03 | 115/11234, 0.01 | 0.00034 |
|  | **neuron projection morphogenesis** | 117/1119, 0.10 | 740/11234, 0.07 | 0.00035 |
|  | cell-cell signaling | 106/1119, 0.09 | 653/11234, 0.06 | 0.00037 |
|  | cellular developmental process | 275/1119, 0.25 | 2107/11234, 0.19 | 0.00043 |
|  | **compound eye photoreceptor development** | 43/1119, 0.04 | 191/11234, 0.02 | 0.00044 |
|  | **neurogenesis** | 199/1119, 0.18 | 1433/11234, 0.13 | 0.00045 |
|  | **negative regulation of microtubule polymerization or depolymerization** | 12/1119, 0.01 | 22/11234, 0.00 | 0.00047 |
|  | **neuron projection development** | 117/1119, 0.10 | 744/11234, 0.07 | 0.00047 |
|  | **negative regulation of cellular component organization** | 39/1119, 0.03 | 167/11234, 0.01 | 0.0006 |
|  | **cell part morphogenesis** | 122/1119, 0.11 | 789/11234, 0.07 | 0.00065 |
|  | **cell projection morphogenesis** | 122/1119, 0.11 | 789/11234, 0.07 | 0.00065 |
|  | **cellular process** | 931/1119, 0.83 | 8705/11234, 0.77 | 0.00089 |
|  | **female mating behavior** | 14/1119, 0.01 | 31/11234, 0.00 | 0.00094 |
|  | **photoreceptor cell development** | 44/1119, 0.04 | 204/11234, 0.02 | 0.00116 |
|  | muscle fiber development | 30/1119, 0.03 | 115/11234, 0.01 | 0.00116 |
|  | **eye photoreceptor cell development** | 43/1119, 0.04 | 198/11234, 0.02 | 0.00128 |
|  | **regulation of dendrite morphogenesis** | 21/1119, 0.02 | 65/11234, 0.01 | 0.00133 |
|  | regulation of cell development | 64/1119, 0.06 | 346/11234, 0.03 | 0.00137 |
|  | **cellular component organization or biogenesis** | 321/1119, 0.29 | 2559/11234, 0.23 | 0.00152 |
|  | **neuron recognition** | 55/1119, 0.05 | 283/11234, 0.03 | 0.00171 |
|  | **calcium ion transport** | 21/1119, 0.02 | 66/11234, 0.01 | 0.00177 |
|  | **regulation of axon extension involved in axon guidance** | 17/1119, 0.02 | 46/11234, 0.00 | 0.00183 |
|  | anatomical structure development | 367/1119, 0.33 | 2994/11234, 0.27 | 0.00186 |
|  | regulation of anatomical structure morphogenesis | 61/1119, 0.05 | 327/11234, 0.03 | 0.00187 |
|  | regulation of nervous system development | 56/1119, 0.05 | 291/11234, 0.03 | 0.00189 |
|  | **regulation of dendrite development** | 21/1119, 0.02 | 67/11234, 0.01 | 0.00233 |
|  | **cell recognition** | 55/1119, 0.05 | 286/11234, 0.03 | 0.00242 |
|  | developmental growth | 46/1119, 0.04 | 223/11234, 0.02 | 0.00246 |
|  | **negative regulation of microtubule depolymerization** | 11/1119, 0.01 | 21/11234, 0.00 | 0.00261 |
|  | **cell morphogenesis** | 133/1119, 0.12 | 902/11234, 0.08 | 0.00281 |
|  | anatomical structure morphogenesis | 248/1119, 0.22 | 1910/11234, 0.17 | 0.00331 |
|  | **regulation of chemotaxis** | 18/1119, 0.02 | 53/11234, 0.00 | 0.00369 |
|  | **cell adhesion** | 58/1119, 0.05 | 312/11234, 0.03 | 0.0039 |
|  | developmental process | 396/1119, 0.35 | 3290/11234, 0.29 | 0.00396 |
|  | **calcium-dependent cell-cell adhesion** | 19/1119, 0.02 | 59/11234, 0.01 | 0.00478 |
|  | organ development | 223/1119, 0.20 | 1696/11234, 0.15 | 0.00528 |
|  | **biological adhesion** | 58/1119, 0.05 | 315/11234, 0.03 | 0.00535 |
|  | signal transduction | 173/1119, 0.15 | 1258/11234, 0.11 | 0.00624 |
|  | **determination of muscle attachment site** | 8/1119, 0.01 | 12/11234, 0.00 | 0.00676 |
|  | **R8 cell development** | 16/1119, 0.01 | 45/11234, 0.00 | 0.00696 |
|  | **cell projection organization** | 131/1119, 0.12 | 902/11234, 0.08 | 0.00767 |
|  | **deactivation of rhodopsin mediated signaling** | 10/1119, 0.01 | 19/11234, 0.00 | 0.00767 |
|  | **regulation of rhodopsin mediated signaling pathway** | 10/1119, 0.01 | 19/11234, 0.00 | 0.00767 |
|  | **regulation of system process** | 34/1119, 0.03 | 151/11234, 0.01 | 0.00821 |
|  | **regulation of cell growth** | 24/1119, 0.02 | 89/11234, 0.01 | 0.00832 |
|  | **larval behavior** | 14/1119, 0.01 | 36/11234, 0.00 | 0.00842 |
|  | **cell-cell adhesion mediated by integrin** | 15/1119, 0.01 | 41/11234, 0.00 | 0.00935 |
|  | **cell-cell adhesion mediated by cadherin** | 15/1119, 0.01 | 41/11234, 0.00 | 0.00935 |
|  | neuromuscular synaptic transmission | 16/1119, 0.01 | 46/11234, 0.00 | 0.00969 |
|  | **anatomical structure homeostasis** | 31/1119, 0.03 | 134/11234, 0.01 | 0.01204 |
|  | **negative regulation of cytoskeleton organization** | 14/1119, 0.01 | 37/11234, 0.00 | 0.01231 |
|  | **negative regulation of protein complex disassembly** | 12/1119, 0.01 | 28/11234, 0.00 | 0.01252 |
|  | **negative regulation of protein depolymerization** | 12/1119, 0.01 | 28/11234, 0.00 | 0.01252 |
|  | **ommatidial rotation** | 20/1119, 0.02 | 68/11234, 0.01 | 0.01256 |
|  | **retinal ganglion cell axon guidance** | 17/1119, 0.02 | 52/11234, 0.00 | 0.01289 |
|  | **regulation of microtubule depolymerization** | 11/1119, 0.01 | 24/11234, 0.00 | 0.01395 |
|  | **negative regulation of organelle organization** | 19/1119, 0.02 | 63/11234, 0.01 | 0.01424 |
|  | regulation of multicellular organismal process | 117/1119, 0.10 | 798/11234, 0.07 | 0.01699 |
|  | **cell adhesion mediated by integrin** | 15/1119, 0.01 | 43/11234, 0.00 | 0.01844 |
|  | cellular component maintenance | 15/1119, 0.01 | 43/11234, 0.00 | 0.01844 |
|  | **compound eye morphogenesis** | 67/1119, 0.06 | 397/11234, 0.04 | 0.02053 |
|  | **regulation of developmental growth** | 40/1119, 0.04 | 198/11234, 0.02 | 0.02058 |
|  | multicellular organismal development | 356/1119, 0.32 | 2960/11234, 0.26 | 0.02072 |
|  | **multi-organism reproductive process** | 35/1119, 0.03 | 164/11234, 0.01 | 0.02113 |
|  | **divalent metal ion transport** | 21/1119, 0.02 | 76/11234, 0.01 | 0.02186 |
|  | **divalent inorganic cation transport** | 21/1119, 0.02 | 76/11234, 0.01 | 0.02186 |
|  | **striated muscle tissue development** | 33/1119, 0.03 | 151/11234, 0.01 | 0.02225 |
|  | **regulation of behavior** | 25/1119, 0.02 | 100/11234, 0.01 | 0.02281 |
|  | **regulation of cell size** | 27/1119, 0.02 | 114/11234, 0.01 | 0.03013 |
|  | **compound eye development** | 73/1119, 0.07 | 451/11234, 0.04 | 0.03691 |
|  | regulation of secretion | 19/1119, 0.02 | 67/11234, 0.01 | 0.03812 |
|  | regulation of cell differentiation | 73/1119, 0.07 | 452/11234, 0.04 | 0.03988 |
|  | **skeletal muscle tissue development** | 31/1119, 0.03 | 142/11234, 0.01 | 0.04175 |
|  | **regulation of protein depolymerization** | 12/1119, 0.01 | 31/11234, 0.00 | 0.04377 |
|  | **lumen formation, open** **tracheal system** | 7/1119, 0.01 | 11/11234, 0.00 | 0.04581 |
|  | reproductive behavior | 35/1119, 0.03 | 170/11234, 0.02 | 0.04782 |
|  | **negative regulation of neurogenesis** | 19/1119, 0.02 | 68/11234, 0.01 | 0.04803 |
| **24h** | cell communication | 383/1501, 0.26 | 1894/11234, 0.17 | 6.83E-17 |
|  | signaling | 365/1501, 0.24 | 1813/11234, 0.16 | 1.72E-15 |
|  | single organism signaling | 365/1501, 0.24 | 1813/11234, 0.16 | 1.72E-15 |
|  | biological regulation | 688/1501, 0.46 | 4060/11234, 0.36 | 1.65E-13 |
|  | response to stimulus | 529/1501, 0.35 | 3019/11234, 0.27 | 2.43E-11 |
|  | regulation of biological process | 634/1501, 0.42 | 3779/11234, 0.34 | 1.21E-10 |
|  | signal transduction | 257/1501, 0.17 | 1258/11234, 0.11 | 1.96E-10 |
|  | regulation of cellular process | 588/1501, 0.39 | 3480/11234, 0.31 | 6.01E-10 |
|  | single-organism process | 1002/1501, 0.67 | 6571/11234, 0.58 | 2.56E-09 |
|  | single-organism cellular process | 861/1501, 0.57 | 5513/11234, 0.49 | 7.10E-09 |
|  | synapse organization | 48/1501, 0.03 | 141/11234, 0.01 | 5.63E-07 |
|  | synaptic transmission | 102/1501, 0.07 | 426/11234, 0.04 | 2.81E-06 |
|  | transmission of nerve impulse | 106/1501, 0.07 | 454/11234, 0.04 | 5.92E-06 |
|  | multicellular organismal signaling | 106/1501, 0.07 | 455/11234, 0.04 | 6.73E-06 |
|  | cell-cell signaling | 138/1501, 0.09 | 653/11234, 0.06 | 2.50E-05 |
|  | cell surface receptor signaling pathway | 129/1501, 0.09 | 605/11234, 0.05 | 4.48E-05 |
|  | cellular response to stimulus | 298/1501, 0.20 | 1709/11234, 0.15 | 0.00026 |
|  | regulation of neuron differentiation | 43/1501, 0.03 | 145/11234, 0.01 | 0.00046 |
|  | multicellular organismal process | 567/1501, 0.38 | 3596/11234, 0.32 | 0.0005 |
|  | system process | 173/1501, 0.12 | 903/11234, 0.08 | 0.00052 |
|  | regulation of axonogenesis | 31/1501, 0.02 | 91/11234, 0.01 | 0.00083 |
|  | single-multicellular organism process | 543/1501, 0.36 | 3438/11234, 0.31 | 0.00091 |
|  | cell development | 254/1501, 0.17 | 1438/11234, 0.13 | 0.00097 |
|  | single-organism developmental process | 435/1501, 0.29 | 2676/11234, 0.24 | 0.00102 |
|  | regulation of cell communication | 148/1501, 0.10 | 756/11234, 0.07 | 0.00114 |
|  | regulation of signaling | 148/1501, 0.10 | 756/11234, 0.07 | 0.00114 |
|  | regulation of anatomical structure morphogenesis | 76/1501, 0.05 | 327/11234, 0.03 | 0.00136 |
|  | neurological system process | 160/1501, 0.11 | 835/11234, 0.07 | 0.00154 |
|  | regulation of biological quality | 200/1501, 0.13 | 1091/11234, 0.10 | 0.00154 |
|  | regulation of multicellular organismal process | 154/1501, 0.10 | 798/11234, 0.07 | 0.00164 |
|  | regulation of cell morphogenesis | 63/1501, 0.04 | 257/11234, 0.02 | 0.00185 |
|  | regulation of neurogenesis | 49/1501, 0.04 | 184/11234, 0.02 | 0.00248 |
|  | **tube fusion** | 17/1501, 0.01 | 37/11234, 0.00 | 0.00337 |
|  | **branch fusion, open tracheal system** | 17/1501, 0.01 | 37/11234, 0.00 | 0.00337 |
|  | regulation of transmission of nerve impulse | 31/1501, 0.02 | 98/11234, 0.01 | 0.00516 |
|  | calcium ion transmembrane transport | 18/1501, 0.01 | 42/11234, 0.00 | 0.00578 |
|  | energy taxis | 13/1501, 0.01 | 24/11234, 0.00 | 0.0058 |
|  | phototaxis | 13/1501, 0.01 | 24/11234, 0.00 | 0.0058 |
|  | regulation of neuron projection development | 35/1501, 0.02 | 118/11234, 0.01 | 0.00624 |
|  | regulation of cell differentiation | 95/1501, 0.06 | 452/11234, 0.04 | 0.00696 |
|  | synapse assembly | 23/1501, 0.02 | 63/11234, 0.01 | 0.00697 |
|  | synaptic vesicle transport | 34/1501, 0.02 | 114/11234, 0.01 | 0.00754 |
|  | regulation of synaptic transmission | 30/1501, 0.02 | 95/11234, 0.01 | 0.0078 |
|  | regulation of neurological system process | 31/1501, 0.02 | 100/11234, 0.01 | 0.00832 |
|  | skeletal muscle organ development | 48/1501, 0.03 | 186/11234, 0.02 | 0.00835 |
|  | regulation of response to stimulus | 162/1501, 0.11 | 872/11234, 0.08 | 0.00913 |
|  | **branching involved in open tracheal system development** | 23/1501, 0.02 | 64/11234, 0.01 | 0.00951 |
|  | system development | 406/1501, 0.27 | 2526/11234, 0.22 | 0.01077 |
|  | regulation of axon extension | 21/1501, 0.01 | 56/11234, 0.00 | 0.01141 |
|  | regulation of extent of cell growth | 21/1501, 0.01 | 56/11234, 0.00 | 0.01141 |
|  | **morphogenesis of a branching structure** | 24/1501, 0.02 | 69/11234, 0.01 | 0.01142 |
|  | **branching morphogenesis of an epithelial tube** | 24/1501, 0.02 | 69/11234, 0.01 | 0.01142 |
|  | **morphogenesis of a branching** **epithelium** | 24/1501, 0.02 | 69/11234, 0.01 | 0.01142 |
|  | neurotransmitter transport | 39/1501, 0.03 | 141/11234, 0.01 | 0.01146 |
|  | anatomical structure development | 472/1501, 0.31 | 2994/11234, 0.27 | 0.01169 |
|  | regulation of cell development | 76/1501, 0.05 | 346/11234, 0.03 | 0.01321 |
|  | organ development | 285/1501, 0.19 | 1696/11234, 0.15 | 0.01464 |
|  | anatomical structure morphogenesis | 316/1501, 0.21 | 1910/11234, 0.17 | 0.01609 |
|  | imaginal disc development | 127/1501, 0.08 | 658/11234, 0.06 | 0.01647 |
|  | instar larval or pupal development | 125/1501, 0.08 | 648/11234, 0.06 | 0.02008 |
|  | intracellular signal transduction | 93/1501, 0.06 | 451/11234, 0.04 | 0.0202 |
|  | regulation of neurotransmitter levels | 35/1501, 0.02 | 124/11234, 0.01 | 0.02132 |
|  | neuromuscular synaptic transmission | 18/1501, 0.01 | 46/11234, 0.00 | 0.02713 |
|  | **wing disc morphogenesis** | 90/1501, 0.06 | 437/11234, 0.04 | 0.02973 |
|  | imaginal disc morphogenesis | 108/1501, 0.07 | 548/11234, 0.05 | 0.03226 |
|  | post-embryonic organ morphogenesis | 108/1501, 0.07 | 549/11234, 0.05 | 0.03505 |
|  | regulation of nervous system development | 65/1501, 0.04 | 291/11234, 0.03 | 0.03557 |
|  | developmental process | 509/1501, 0.34 | 3290/11234, 0.29 | 0.03899 |
|  | regulation of cell projection organization | 44/1501, 0.03 | 175/11234, 0.02 | 0.04275 |
|  | regulation of cell morphogenesis involved in differentiation | 35/1501, 0.02 | 128/11234, 0.01 | 0.0454 |
|  | imaginal disc-derived appendage development | /1501, 0.07 | 497/11234, 0.04 | 0.04721 |
| **36h** | regulation of axon extension | 14/538, 0.03 | 56/11234, 0.00 | 0.00034 |
|  | regulation of extent of cell growth | 14/538, 0.03 | 56/11234, 0.00 | 0.00034 |
|  | inositol catabolic process | 7/538, 0.01 | 14/11234, 0.00 | 0.00194 |
|  | **cellular carbohydrate** **catabolic process** | 8/538, 0.01 | 22/11234, 0.00 | 0.00634 |
|  | regulation of axonogenesis | 16/538, 0.03 | 91/11234, 0.01 | 0.00776 |
|  | polyol catabolic process | 7/538, 0.01 | 17/11234, 0.00 | 0.00973 |
|  | **inositol metabolic process** | 7/538, 0.01 | 18/11234, 0.00 | 0.01527 |
|  | negative regulation of peptidoglycan recognition protein signaling pathway | 4/538, 0.01 | 5/11234, 0.00 | 0.03431 |
| **48h** | signaling | 271/1186, 0.23 | 1813/11234, 0.16 | 2.58E-07 |
|  | single organism signaling | 271/1186, 0.23 | 1813/11234, 0.16 | 2.58E-07 |
|  | cell communication | 279/1186, 0.24 | 1894/11234, 0.17 | 6.30E-07 |
|  | response to stimulus | 409/1186, 0.34 | 3019/11234, 0.27 | 1.30E-06 |
|  | post-embryonic genitalia morphogenesis | 13/1186, 0.01 | 18/11234, 0.00 | 2.15E-06 |
|  | imaginal disc-derived male genitalia morphogenesis | 13/1186, 0.01 | 18/11234, 0.00 | 2.15E-06 |
|  | imaginal disc-derived genitalia morphogenesis | 13/1186, 0.01 | 18/11234, 0.00 | 2.15E-06 |
|  | signal transduction | 194/1186, 0.16 | 1258/11234, | 1.99E-05 |
|  | **genital disc morphogenesis** | 13/1186, 0.01 | 22/11234, 0.00 | 8.32E-05 |
|  | **imaginal disc-derived genitalia development** | 13/1186, 0.01 | 23/11234, 0.00 | 0.00017 |
|  | **imaginal disc-derived male genitalia development** | 13/1186, 0.01 | 23/11234, 0.00 | 0.00017 |
|  | **genitalia morphogenesis** | 13/1186, 0.01 | 24/11234, 0.00 | 0.00034 |
|  | **male genitalia morphogenesis** | 13/1186, 0.01 | 24/11234, 0.00 | 0.00034 |
|  | **male genitalia development** | 13/1186, 0.01 | 25/11234, 0.00 | 0.00064 |
|  | hemocyte migration | 13/1186, 0.01 | 25/11234, 0.00 | 0.00064 |
|  | **distributive segregation** | 8/1186, 0.01 | 10/11234, 0.00 | 0.00122 |
|  | **negative regulation of sister chromatid segregation** | 8/1186, 0.01 | 10/11234, 0.00 | 0.00122 |
|  | hemocyte proliferation | 8/1186, 0.01 | 10/11234, 0.00 | 0.00122 |
|  | **negative regulation of chromosome segregation** | 8/1186, 0.01 | 10/11234, 0.00 | 0.00122 |
|  | **negative regulation of chromosome organization** | 11/1186, 0.01 | 19/11234, 0.00 | 0.00129 |
|  | **mitotic centrosome separation** | 9/1186, 0.01 | 13/11234, 0.00 | 0.00167 |
|  | biological regulation | 505/1186, 0.43 | 4060/11234, 0.36 | 0.0017 |
|  | regulation of biological process | 473/1186, 0.40 | 3779/11234, 0.34 | 0.00251 |
|  | regulation of cell shape | 33/1186, 0.03 | 130/11234, 0.01 | 0.00254 |
|  | **negative regulation of response to biotic stimulus** | 6/1186, 0.01 | 6/11234, 0.00 | 0.003 |
|  | **achiasmate meiosis I** | 8/1186, 0.01 | 11/11234, 0.00 | 0.00407 |
|  | **regulation of sister chromatid segregation** | 8/1186, 0.01 | 11/11234, 0.00 | 0.00407 |
|  | **meiotic metaphase I plate** **congression** | 8/1186, 0.01 | 11/11234, 0.00 | 0.00407 |
|  | **meiotic metaphase plate congression** | 8/1186, 0.01 | 11/11234, 0.00 | 0.00407 |
|  | inositol catabolic process | 9/1186, 0.01 | 14/11234, 0.00 | 0.00424 |
|  | regulation of cellular process | 438/1186, 0.37 | 3480/11234, 0.31 | 0.00475 |
|  | **regulation of chromosome organization** | 13/1186, 0.01 | 29/11234, 0.00 | 0.00558 |
|  | **carbohydrate catabolic process** | 24/1186, 0.02 | 83/11234, 0.01 | 0.00632 |
|  | **Toll signaling pathway** | 14/1186, 0.01 | 34/11234, 0.00 | 0.00789 |
|  | **homologous chromosome segregation** | 8/1186, 0.01 | 12/11234, 0.00 | 0.01109 |
|  | **metaphase plate congression** | 8/1186, 0.01 | 12/11234, 0.00 | 0.01109 |
|  | cellular response to stimulus | 234/1186, 0.20 | 1709/11234, 0.15 | 0.01171 |
|  | ventral cord development | 13/1186, 0.01 | 31/11234, 0.00 | 0.01385 |
|  | **development of primary male sexual characteristics** | 13/1186, 0.01 | 31/11234, 0.00 | 0.01385 |
|  | **single-organism carbohydrate catabolic process** | 23/1186, 0.02 | 81/11234, 0.01 | 0.01421 |
|  | protein phosphorylation | 74/1186, 0.06 | 427/11234, 0.04 | 0.02257 |
|  | **embryonic hemopoiesis** | 14/1186, 0.01 | 37/11234, 0.00 | 0.02555 |
|  | **chromosome localization** | 8/1186, 0.01 | 13/11234, 0.00 | 0.02617 |
|  | **establishment of chromosome localization** | 8/1186, 0.01 | 13/11234, 0.00 | 0.02617 |
|  | **regulation of chromosome segregation** | 8/1186, 0.01 | 13/11234, 0.00 | 0.02617 |
|  | **vascular endothelial growth factor receptor signaling pathway** | 5/1186, 0.00 | 5/11234, 0.00 | 0.02855 |
|  | negative regulation of peptidoglycan recognition protein signaling pathway | 5/1186, 0.00 | 5/11234, 0.00 | 0.02855 |
|  | polyol catabolic process | 9/1186, 0.01 | 17/11234, 0.00 | 0.03837 |
|  | **anaphase** | 9/1186, 0.01 | 17/11234, 0.00 | 0.03837 |
| **72h** | response to stimulus | 765/2112, 0.36 | 3019/11234, 0.27 | 1.48E-22 |
|  | signaling | 507/2112, 0.24 | 1813/11234, 0.16 | 2.36E-22 |
|  | single organism signaling | 507/2112, 0.24 | 1813/11234, 0.16 | 2.36E-22 |
|  | cell communication | 524/2112, 0.25 | 1894/11234, 0.17 | 4.17E-22 |
|  | biological regulation | 947/2112, 0.45 | 4060/11234, 0.36 | 1.15E-16 |
|  | signal transduction | 361/2112, 0.17 | 1258/11234, 0.11 | 1.94E-16 |
|  | regulation of biological process | 882/2112, 0.42 | 3779/11234, 0.34 | 8.51E-15 |
|  | regulation of cellular process | 814/2112, 0.39 | 3480/11234, 0.31 | 3.50E-13 |
|  | synapse organization | 64/2112, 0.03 | 141/11234, 0.01 | 1.02E-09 |
|  | cell surface receptor signaling pathway | 183/2112, 0.09 | 605/11234, 0.05 | 5.41E-09 |
|  | system process | 252/2112, 0.12 | 903/11234, 0.08 | 5.87E-09 |
|  | multicellular organismal process | 808/2112, 0.38 | 3596/11234, 0.32 | 2.46E-08 |
|  | single-multicellular organism process | 776/2112, 0.37 | 3438/11234, 0.31 | 3.30E-08 |
|  | cellular response to stimulus | 420/2112, 0.20 | 1709/11234, 0.15 | 1.90E-07 |
|  | single-organism process | 1365/2112, 0.65 | 6571/11234, 0.58 | 2.68E-07 |
|  | single-organism cellular process | 1167/2112, 0.55 | 5513/11234, 0.49 | 4.68E-07 |
|  | neurological system process | 228/2112, 0.11 | 835/11234, 0.07 | 7.30E-07 |
|  | imaginal disc development | 187/2112, 0.09 | 658/11234, 0.06 | 1.12E-06 |
|  | instar larval or pupal development | 183/2112, 0.09 | 648/11234, 0.06 | 3.04E-06 |
|  | regulation of anatomical structure morphogenesis | 106/2112, 0.05 | 327/11234, 0.03 | 4.82E-06 |
|  | **behavior** | 176/2112, 0.08 | 621/11234, 0.06 | 4.84E-06 |
|  | cell-cell signaling | 182/2112, 0.09 | 653/11234, 0.06 | 1.03E-05 |
|  | regulation of cell communication | 205/2112, 0.10 | 756/11234, 0.07 | 1.20E-05 |
|  | regulation of signaling | 205/2112, 0.10 | 756/11234, 0.07 | 1.20E-05 |
|  | **metamorphosis** | 170/2112, 0.08 | 603/11234, 0.05 | 1.42E-05 |
|  | **instar larval or pupal morphogenesis** | 167/2112, 0.08 | 591/11234, 0.05 | 1.64E-05 |
|  | synaptic transmission | 128/2112, 0.06 | 426/11234, 0.04 | 2.14E-05 |
|  | organ development | 405/2112, 0.19 | 1696/11234, 0.15 | 2.46E-05 |
|  | imaginal disc morphogenesis | 156/2112, 0.07 | 548/11234, 0.05 | 3.10E-05 |
|  | regulation of cell morphogenesis | 86/2112, 0.04 | 257/11234, 0.02 | 3.21E-05 |
|  | transmission of nerve impulse | 134/2112, 0.06 | 454/11234, 0.04 | 3.24E-05 |
|  | post-embryonic organ morphogenesis | 156/2112, 0.07 | 549/11234, 0.05 | 3.55E-05 |
|  | multicellular organismal signaling | 134/2112, 0.06 | 455/11234, 0.04 | 3.76E-05 |
|  | developmental process | 725/2112, 0.34 | 3290/11234, 0.29 | 3.83E-05 |
|  | regulation of multicellular organismal process | 212/2112, 0.10 | 798/11234, 0.07 | 4.04E-05 |
|  | developmental growth | 77/2112, 0.04 | 223/11234, 0.02 | 4.07E-05 |
|  | single-organism developmental process | 603/2112, 0.29 | 2676/11234, 0.24 | 4.07E-05 |
|  | **post-embryonic morphogenesis** | 167/2112, 0.08 | 598/11234, 0.05 | 4.09E-05 |
|  | **post-embryonic organ development** | 163/2112, 0.08 | 587/11234, 0.05 | 9.41E-05 |
|  | **single-organism behavior** | 151/2112, 0.07 | 535/11234, 0.05 | 9.96E-05 |
|  | **organ morphogenesis** | 249/2112, 0.12 | 976/11234, 0.09 | 0.0001 |
|  | anatomical structure development | 663/2112, 0.31 | 2994/11234, 0.27 | 0.00011 |
|  | anatomical structure morphogenesis | 444/2112, 0.21 | 1910/11234, 0.17 | 0.00015 |
|  | protein phosphorylation | 125/2112, 0.06 | 427/11234, 0.04 | 0.00017 |
|  | nervous system development | 391/2112, 0.19 | 1658/11234, 0.15 | 0.00022 |
|  | cell development | 345/2112, 0.16 | 1438/11234, 0.13 | 0.00025 |
|  | system development | 567/2112, 0.27 | 2526/11234, 0.22 | 0.00025 |
|  | mating | 48/2112, 0.02 | 123/11234, 0.01 | 0.00034 |
|  | synapse assembly | 30/2112, 0.01 | 63/11234, 0.01 | 0.00051 |
|  | ventral cord development | 19/2112, 0.01 | 31/11234, 0.00 | 0.00059 |
|  | **central nervous system development** | 117/2112, 0.06 | 403/11234, 0.04 | 0.00074 |
|  | regulation of response to stimulus | 222/2112, 0.11 | 872/11234, 0.08 | 0.00074 |
|  | learning | 37/2112, 0.02 | 88/11234, 0.01 | 0.00107 |
|  | **enzyme linked receptor protein signaling pathway** | 54/2112, 0.03 | 150/11234, 0.01 | 0.00128 |
|  | **response to chemical stimulus** | 262/2112, 0.12 | 1065/11234, 0.09 | 0.00134 |
|  | intracellular signal transduction | 127/2112, 0.06 | 451/11234, 0.04 | 0.00144 |
|  | regulation of cell differentiation | 127/2112, 0.06 | 452/11234, 0.04 | 0.00164 |
|  | skeletal muscle organ development | 63/2112, 0.03 | 186/11234, 0.02 | 0.00179 |
|  | mating behavior | 44/2112, 0.02 | 115/11234, 0.01 | 0.00217 |
|  | **adult behavior** | 55/2112, 0.03 | 156/11234, 0.01 | 0.00218 |
|  | multicellular organismal development | 645/2112, 0.31 | 2960/11234, 0.26 | 0.00263 |
|  | **post-embryonic development** | 194/2112, 0.09 | 761/11234, 0.07 | 0.0041 |
|  | response to external stimulus | 180/2112, 0.09 | 698/11234, 0.06 | 0.0046 |
|  | **response to bacterium** | 34/2112, 0.02 | 82/11234, 0.01 | 0.00464 |
|  | muscle organ development | 83/2112, 0.04 | 273/11234, 0.02 | 0.00568 |
|  | **learning or memory** | 61/2112, 0.03 | 184/11234, 0.02 | 0.00607 |
|  | regulation of cell shape | 47/2112, 0.03 | 130/11234, 0.01 | 0.00613 |
|  | hemocyte proliferation | 9/2112, 0.00 | 10/11234, 0.00 | 0.00687 |
|  | **regulation of microtubule polymerization or depolymerization** | 18/2112, 0.01 | 32/11234, 0.00 | 0.00724 |
|  | **cognition** | 61/2112, 0.03 | 185/11234, 0.02 | 0.00742 |
|  | neuromuscular junction development | 35/2112, 0.02 | 87/11234, 0.01 | 0.00758 |
|  | regulation of transmission of nerve impulse | 38/2112, 0.02 | 98/11234, 0.01 | 0.00833 |
|  | taxis | 138/2112, 0.07 | 515/11234, 0.05 | 0.00946 |
|  | **developmental growth involved in morphogenesis** | 52/2112, 0.02 | 151/11234, 0.01 | 0.00967 |
|  | **ionotropic glutamate receptor signaling pathway** | 16/2112, 0.01 | 27/11234, 0.00 | 0.0104 |
|  | **multi-organism behavior** | 66/2112, 0.03 | 207/11234, 0.02 | 0.01083 |
|  | **glycosaminoglycan metabolic process** | 17/2112, 0.01 | 30/11234, 0.00 | 0.01204 |
|  | generation of neurons | 264/2112, 0.13 | 1103/11234, 0.10 | 0.01301 |
|  | **regulation of developmental process** | 182/2112, 0.09 | 719/11234, 0.06 | 0.01423 |
|  | regulation of neurological system process | 38/2112, 0.02 | 100/11234, 0.01 | 0.01469 |
|  | imaginal disc-derived appendage development | 133/2112, 0.06 | 497/11234, 0.04 | 0.01537 |
|  | hemocyte migration | 15/2112, 0.01 | 25/11234, 0.00 | 0.01704 |
|  | skeletal muscle fiber development | 35/2112, 0.02 | 90/11234, 0.01 | 0.0188 |
|  | reproductive behavior | 56/2112, 0.03 | 170/11234, 0.02 | 0.01948 |
|  | **genital disc development** | 22/2112, 0.01 | 46/11234, 0.00 | 0.02035 |
|  | cellular component maintenance | 21/2112, 0.01 | 43/11234, 0.00 | 0.02168 |
|  | regulation of biological quality | 260/2112, 0.12 | 1091/11234, 0.10 | 0.02202 |
|  | regulation of synaptic transmission | 36/2112, 0.02 | 95/11234, 0.01 | 0.02778 |
|  | **regulation of signal transduction** | 165/2112, 0.08 | 648/11234, 0.06 | 0.02805 |
|  | cell differentiation | 458/2112, 0.22 | 2064/11234, 0.18 | 0.02854 |
|  | **mushroom body development** | 46/2112, 0.02 | 133/11234, 0.01 | 0.03063 |
|  | **imaginal disc-derived appendage morphogenesis** | 130/2112, 0.06 | 490/11234, 0.04 | 0.0312 |
|  | **anesthesia-resistant memory** | 9/2112, 0.00 | 11/11234, 0.00 | 0.03142 |
|  | post-embryonic genitalia morphogenesis | 12/2112, 0.01 | 18/11234, 0.00 | 0.03241 |
|  | imaginal disc-derived male genitalia morphogenesis | 12/2112, 0.01 | 18/11234, 0.00 | 0.03241 |
|  | imaginal disc-derived genitalia morphogenesis | 12/2112, 0.01 | 18/11234, 0.00 | 0.03241 |
|  | neuron development | 217/2112, 0.10 | 893/11234, 0.08 | 0.03523 |
|  | **regulation of microtubule cytoskeleton organization** | 20/2112, 0.01 | 41/11234, 0.00 | 0.0365 |
|  | **associative learning** | 31/2112, 0.01 | 78/11234, 0.01 | 0.03732 |
|  | regulation of cell development | 97/2112, 0.05 | 346/11234, 0.03 | 0.0394 |
|  | muscle fiber development | 41/2112, 0.02 | 115/11234, 0.01 | 0.0397 |
|  | **multi-organism process** | 125/2112, 0.06 | 470/11234, 0.04 | 0.04062 |
|  | **adult locomotory behavior** | 32/2112, 0.02 | 82/11234, 0.01 | 0.04246 |
|  | cellular developmental process | 465/2112, 0.22 | 2107/11234, 0.19 | 0.04388 |
|  | **response to abiotic stimulus** | 105/2112, 0.05 | 382/11234, 0.03 | 0.04468 |

**Table S6. CNS process-related DEGs (log_2_ratio)**

| **Pathway/**  **member** | **Unigene** | **4 h pi** | **12 h pi** | | **24 h pi** | **36 h pi** | **48 h pi** | **72 h pi** |
| --- | --- | --- | --- | --- | --- | --- | --- | --- |
| **Serotonergic synapse pathway** | |  |  |  | |  |  |  |
| PLC | CL2281.Contig1_All | -1.14 | -0.82 | -1.53 | | -0.49 | -1.13 | -1.74 |
| VGCC | CL2283.Contig1_All | -2.48 | -2.77 | -3.26 | | -2.93 | -2.57 | -2.51 |
| Raf | CL3009.Contig1_All | -1.06 | -0.45 | -0.79 | | -1.14 | -1.27 | -1.68 |
| PKA | CL32.Contig8_All | -1.16 | -0.65 | -1.41 | | -0.29 | -1.31 | -2.30 |
| DDC | CL3484.Contig2_All | -1.11 | 0.39 | -0.61 | | 0.10 | -1.06 | 0.47 |
| Raf | CL6905.Contig2_All | -1.87 | -2.52 | -2.53 | | -1.64 | -1.71 | -2.73 |
| Gi/o | CL7798.Contig2_All | -1.09 | -1.63 | -0.62 | | -1.26 | -0.92 | -0.96 |
| DCC | CL8446.Contig3_All | -2.37 | -1.73 | -1.68 | | 0.02 | -2.64 | -2.64 |
| Gi/o | Unigene1049_All | -1.10 | -0.39 | -0.98 | | -0.48 | -1.39 | -1.56 |
| Gs | Unigene10560_All | -1.54 | -2.27 | -2.10 | | -0.44 | -0.82 | -1.16 |
| ERK | Unigene12163_All | -1.48 | -0.86 | -1.60 | | -0.47 | -1.43 | -2.05 |
| PLA2 | Unigene12178_All | -1.07 | 0.11 | -0.08 | | -0.32 | -0.89 | -0.21 |
| Gi/o | Unigene13908_All | -2.07 | -1.47 | -2.32 | | -0.54 | -2.02 | -2.61 |
| PKA | Unigene14542_All | -1.47 | -0.76 | -1.76 | | -0.13 | -1.40 | -2.30 |
| PKC | Unigene40306_All | -2.98 | -1.19 | -2.48 | | -0.60 | -1.64 | -2.37 |
| VMAT | Unigene66086_All | -1.73 | -0.80 | -1.46 | | -0.43 | -1.50 | -3.17 |
| VMAT | Unigene72668_All | -1.52 | -0.84 | -1.55 | | -0.53 | -2.00 | -2.62 |
| 5-HT1AF | Unigene76812_All | -3.27 | -2.94 | -3.74 | | -0.86 | -2.38 | -10.53 |
| Raf | Unigene78202_All | 1.09 | 0.60 | 0.27 | | 0.26 | 0.67 | -0.36 |
| Raf | Unigene7991_All | -1.29 | -1.44 | -1.39 | | -1.31 | -0.92 | -0.55 |
| Raf | CL2444.Contig1_All | -0.32 | -1.23 | -0.97 | | -0.66 | -0.23 | -0.47 |
| 5HT4,6,7  5-HT1AF | CL2914.Contig2_All | -1.57 | -2.16 | -2.56 | | -0.80 | -2.64 | -4.39 |
| Gi/o | CL7798.Contig1_All | -0.73 | -1.73 | -1.04 | | -0.82 | -1.10 | -1.07 |
| 5-HT1AF | CL8776.Contig1_All | -2.85 | -3.54 | -10.31 | | 1.10 | -8.77 | -10.48 |
| MAO-A | Unigene11212_All | 0.44 | -3.27 | 0.37 | | 5.31 | 0.80 | 0.63 |
| Raf | Unigene11367_All | -0.56 | -3.38 | 0.39 | | -1.35 | -1.75 | -4.69 |
| VGCC | Unigene12299_All | -0.87 | -1.54 | 0.79 | | -0.91 | 0.72 | 0.50 |
| Raf | Unigene29393_All | 0.08 | -2.12 | -0.34 | | -0.69 | -0.62 | -0.32 |
| 5-HT1AF | Unigene31347_All | -2.85 | -3.40 | -3.04 | | -1.29 | -2.64 | -4.43 |
| 5-HT3 | Unigene6061_All | 1.49 | 1.58 | 0.99 | | -0.26 | -0.27 | -0.50 |
| PKC | Unigene72446_All | -0.97 | -1.17 | -1.91 | | -0.39 | -1.44 | -1.91 |
| PLA2 | Unigene80923_All | -2.21 | -2.14 | -1.56 | | 1.76 | -2.86 | -2.49 |
| PLC | CL2281.Contig2_All | -0.92 | -1.00 | -1.49 | | -0.40 | -1.28 | -2.48 |
| Raf | CL4034.Contig4_All | -1.27 | -0.42 | -2.71 | | -1.98 | -3.14 | -2.59 |
| VGCC | CL4555.Contig2_All | -8.68 | -1.34 | -9.66 | | -6.70 | -7.73 | -9.61 |
| Raf | CL6948.Contig1_All | -2.18 | -0.21 | -2.36 | | -1.31 | -1.86 | -0.88 |
| Raf | CL8054.Contig1_All | -0.53 | 0.51 | -3.44 | | -1.12 | -0.27 | 0.35 |
| Ras | Unigene11311_All | -0.86 | -0.46 | -1.08 | | -0.54 | -0.88 | -0.98 |
| PKC | Unigene11828_All | -0.94 | -0.91 | -1.36 | | -0.75 | -1.71 | -1.64 |
| SERT | Unigene63179_All | -0.83 | -0.55 | -2.37 | | -1.09 | -2.86 | -2.18 |
| 5-HT4,6,7 | Unigene65131_All | -1.42 | -1.34 | -4.63 | | -0.94 | -0.86 | -1.11 |
| DDC | CL2735.Contig4_All | -0.89 | -0.51 | -0.88 | | -1.03 | -0.43 | -0.56 |
| 5-HT4,6,7 | Unigene65132_All | -1.85 | -1.36 | -1.63 | | -3.76 | -1.47 | -1.22 |
| PKC | CL11461.Contig1_All | -0.70 | -1.98 | -1.94 | | -1.20 | -2.86 | -2.01 |
| Raf | CL4304.Contig1_All | -0.21 | 0.07 | -0.67 | | -1.12 | -1.25 | -0.40 |
| Gi/o | CL5187.Contig2_All | -0.88 | 0.42 | 0.41 | | -0.14 | -1.00 | -0.51 |
| VGCC | Unigene12233_All | 0.36 | 0.63 | 0.62 | | 0.39 | 1.39 | 1.33 |
| MAO-A | Unigene7582_All | 0.26 | 0.26 | 0.91 | | -0.68 | -1.00 | -0.24 |
| VMAT | Unigene8858_All | -0.10 | 0.88 | -0.16 | | -0.48 | -2.04 | -1.25 |
| MAO-A | Unigene9218_All | -0.98 | -0.16 | 0.53 | | -0.79 | -1.62 | -0.78 |
| PKC | CL11461.Contig3_All | -2.27 | -2.17 | -2.46 | | -0.12 | -1.86 | -3.43 |
| PLC | CL11624.Contig1_All | -0.61 | -0.92 | -0.27 | | -0.82 | -0.53 | -1.99 |
| 5-HT1AF | CL5867.Contig3_All | -1.27 | -1.74 | -1.89 | | -1.12 | -1.37 | -2.98 |
| 5-HT1AF | Unigene1894_All | -0.40 | -0.43 | -0.70 | | -0.40 | -0.55 | -1.21 |
| 5-HT2A 5HT2 | Unigene39879_All | -0.39 | 0.04 | -0.33 | | -0.19 | -1.00 | -1.30 |
| SERT | Unigene63180_All | -1.87 | -0.78 | -1.00 | | -0.98 | -2.01 | -2.82 |
| SERT | Unigene63181_All | -10.34 | -0.18 | -2.37 | | -1.20 | -1.22 | -3.61 |
| 5-HT4,6,7 | Unigene81448_All | -0.70 | -0.71 | -1.58 | | -0.82 | -2.75 | -2.69 |
| Gi/o | Unigene99_All | -0.43 | -0.21 | -0.84 | | -0.24 | -0.46 | -1.23 |
| **Synaptic vesicle cycle pathway** | |  |  |  | |  |  |  |
| Complexin | CL1279.Contig2_All | -1.74 | -1.06 | -1.79 | | -0.71 | -1.13 | -2.48 |
| SYT | CL2229.Contig2_All | -1.14 | -0.75 | -1.59 | | -0.68 | -1.38 | -2.19 |
| VGCC | CL2283.Contig1_All | -2.48 | -2.77 | -3.26 | | -2.93 | -2.57 | -2.51 |
| Munc18 | CL5027.Contig2_All | -1.06 | -0.89 | -1.25 | | -0.65 | -0.89 | -1.78 |
| Dynamin | CL5954.Contig1_All | -1.37 | -1.32 | -1.61 | | -0.62 | -1.45 | -2.22 |
| SYT | CL9819.Contig1_All | -2.20 | -0.68 | -1.58 | | -0.43 | -1.41 | -1.69 |
| NSF | Unigene10660_All | -1.61 | -0.91 | -1.54 | | -0.06 | -1.85 | -2.62 |
| SYT | Unigene11228_All | -1.52 | -0.98 | -1.82 | | -0.75 | -1.56 | -2.43 |
| AP2 | Unigene15223_All | -1.16 | -0.70 | -1.11 | | -0.45 | -0.96 | -1.33 |
| Munc18 | Unigene2617_All | -2.34 | -1.73 | -2.79 | | -0.54 | -1.92 | -3.19 |
| VAMP | Unigene31635_All | -2.20 | -1.51 | -2.77 | | -1.40 | -1.77 | -3.72 |
| Munc13 | Unigene3340_All | -1.10 | -0.59 | -1.38 | | -0.45 | -1.52 | -1.47 |
| Munc13 | Unigene40306_All | -2.98 | -1.19 | -2.48 | | -0.60 | -1.64 | -2.37 |
| SYT | Unigene40782_All | -2.13 | -1.97 | -2.81 | | 0.35 | -1.64 | -2.69 |
| Transpoter | Unigene4955_All | -2.36 | -3.65 | -3.73 | | -1.94 | -2.31 | -2.69 |
| NSF | Unigene5790_All | -1.30 | -0.87 | -1.28 | | -0.29 | -1.26 | -1.73 |
| RIM | Unigene58140_All | -3.37 | -2.84 | -3.34 | | -1.88 | -3.22 | -4.75 |
| Clathrin | Unigene5885_All | -1.68 | -1.13 | -1.71 | | -0.31 | -1.53 | -2.30 |
| Munc13 | Unigene65543_All | -2.76 | -2.51 | -2.77 | | -1.19 | -2.42 | -3.84 |
| Transpoter | Unigene66086_All | -1.73 | -0.80 | -1.46 | | -0.43 | -1.50 | -3.17 |
| Munc13 | Unigene66181_All | -2.73 | -3.18 | -3.61 | | -0.97 | -1.95 | -3.34 |
| Transpoter | Unigene72668_All | -1.52 | -0.84 | -1.55 | | -0.53 | -2.00 | -2.62 |
| SYT | Unigene74950_All | -2.15 | -1.91 | -2.36 | | -0.62 | -1.57 | -3.47 |
| Transpoter | Unigene76468_All | -4.50 | -2.90 | -3.39 | | -0.02 | -1.54 | -4.43 |
| Munc13 | Unigene77842_All | -2.28 | -2.44 | -4.72 | | -2.70 | -4.30 | -4.73 |
| VAMP | Unigene80748_All | 1.25 | 0.99 | 1.14 | | 0.76 | 0.98 | 0.79 |
| αSNAP | Unigene9248_All | -2.36 | -1.61 | -2.67 | | -0.31 | -2.08 | -3.06 |
| Syntaxin | CL11.Contig3_All | -1.04 | -1.83 | -1.31 | | -0.21 | -1.81 | -2.40 |
| Syntaxin | CL11.Contig4_All | -1.12 | -2.47 | -1.70 | | -0.26 | -1.73 | -2.74 |
| SYT | CL1177.Contig2_All | -0.67 | 1.48 | -0.04 | | 0.38 | -0.29 | -1.11 |
| Dynamin | CL5954.Contig2_All | -0.93 | -1.75 | -1.31 | | -1.88 | -0.93 | -2.49 |
| Transpoter | CL9844.Contig2_All | 0.21 | -1.19 | 0.10 | | -0.08 | 1.21 | 0.92 |
| VGCC | Unigene12299_All | -0.87 | -1.54 | 0.79 | | -0.91 | 0.72 | 0.50 |
| SYT | Unigene13018_All | -0.83 | -1.14 | -0.69 | | -0.55 | -0.39 | -0.70 |
| SYT | Unigene14606_All | -0.78 | -1.37 | -0.78 | | -0.96 | -0.50 | -0.72 |
| Munc13 | Unigene14685_All | -1.27 | -5.08 | -2.13 | | -1.81 | -1.64 | -1.78 |
| Transpoter | Unigene5066_All | -0.74 | -1.37 | -0.90 | | -1.06 | -0.66 | -0.86 |
| Munc13 | Unigene65544_All | -2.44 | -2.18 | -2.95 | | -2.44 | -3.75 | -3.73 |
| SYT | Unigene75977_All | -0.88 | -1.36 | -0.85 | | -0.88 | -0.71 | -0.98 |
| NSF | Unigene8621_All | -0.99 | -1.05 | -1.75 | | 0.19 | -1.36 | -3.28 |
| V-ATPase | CL13055.Contig1_All | -0.80 | -0.82 | -1.08 | | -0.84 | -1.22 | -1.00 |
| V-ATPase | CL3817.Contig4_All | -0.53 | -1.66 | -8.83 | | 0.88 | 8.31 | 1.11 |
| VGCC | CL4555.Contig2_All | -8.68 | -1.34 | -9.66 | | -6.70 | -7.73 | -9.61 |
| Transpoter | CL6277.Contig1_All | -3.21 | -0.69 | -2.69 | | -1.23 | -3.05 | -2.77 |
| Transpoter | Unigene27251_All | -0.19 | -0.41 | -0.92 | | -1.63 | -0.19 | 0.01 |
| Transpoter | Unigene3045_All | -0.80 | -0.18 | -0.62 | | -1.19 | 0.26 | 0.25 |
| Transpoter | Unigene26255_All | -1.53 | -0.99 | -1.40 | | -0.97 | -2.49 | -1.72 |
| Transpoter | Unigene8858_All | -0.10 | 0.88 | -0.16 | | -0.48 | -2.04 | -1.25 |
| V-ATPase | CL2182.Contig2_All | -1.30 | -0.90 | -1.99 | | -1.12 | -1.64 | -2.69 |
| Munc13 | CL4990.Contig1_All | -0.90 | -0.85 | -0.97 | | -0.76 | -0.81 | -1.08 |
| SYT | CL6003.Contig1_All | -1.04 | 0.27 | -1.27 | | -1.12 | -1.22 | -6.76 |
| V-ATPase | CL9005.Contig2_All | -0.99 | -0.37 | -0.96 | | 0.02 | -1.05 | -1.22 |
| Transpoter | CL9007.Contig1_All | -0.92 | -0.15 | -0.99 | | -0.10 | -0.54 | -1.06 |
| RIM | Unigene62797_All | -0.70 | -1.66 | -1.55 | | 0.30 | -8.92 | -11.22 |
| SYT | Unigene64056_All | -0.64 | -0.64 | -0.51 | | -0.49 | -0.53 | -1.07 |
| SYT | Unigene68869_All | -0.89 | -0.30 | -0.60 | | 0.62 | -0.05 | -1.09 |
| Munc13 | Unigene82728_All | -3.63 | -2.88 | -2.04 | | 1.88 | 0.00 | -3.28 |
| SYT | Unigene9573_All | -0.40 | 0.61 | -0.13 | | -0.45 | -0.85 | -1.08 |
| **Cholinergic synapse pathway** | |  |  |  | |  |  |  |
| AChE | CL11727.Contig2_All | -4.69 | -2.14 | -3.37 | | -0.12 | -7.91 | -4.57 |
| AC | CL12536.Contig1_All | -1.52 | -1.25 | -2.49 | | -1.78 | -1.27 | -1.11 |
| AC | CL12536.Contig2_All | -1.58 | -1.58 | -1.48 | | -1.54 | -1.12 | -1.05 |
| CaMK | CL12768.Contig1_All | -1.40 | -1.27 | -0.77 | | -0.24 | -0.74 | -1.11 |
| CaMK | CL12768.Contig2_All | -1.73 | -1.47 | -1.69 | | -0.18 | -1.89 | -2.90 |
| CaMK | CL12768.Contig3_All | -2.01 | -2.16 | -2.20 | | -0.40 | -1.39 | -2.38 |
| CHT | CL135.Contig3_All | -1.14 | -1.70 | -2.03 | | -1.72 | -1.18 | -1.45 |
| PLC | CL2281.Contig1_All | -1.14 | -0.82 | -1.53 | | -0.49 | -1.13 | -1.74 |
| VGCC | CL2283.Contig1_All | -2.48 | -2.77 | -3.26 | | -2.93 | -2.57 | -2.51 |
| PKA | CL32.Contig8_All | -1.16 | -0.65 | -1.41 | | -0.29 | -1.31 | -2.30 |
| Kvτlkir2 | CL3275.Contig5_All | -1.25 | -1.28 | -1.15 | | -1.39 | -0.88 | -1.53 |
| CaMK | CL5029.Contig1_All | -1.33 | -1.30 | -1.70 | | 0.11 | -1.30 | -1.78 |
| AC | CL5341.Contig1_All | -1.38 | -1.33 | -2.23 | | -1.28 | -1.54 | -2.65 |
| AC | CL6305.Contig1_All | -7.51 | -2.51 | -2.18 | | 0.05 | -1.37 | -3.35 |
| AC | CL6305.Contig2_All | -2.49 | -1.87 | -3.31 | | -2.93 | -0.40 | -3.66 |
| Giα | CL7798.Contig2_All | -1.09 | -1.63 | -0.62 | | -1.26 | -0.92 | -0.96 |
| Giα | Unigene1049_All | -1.10 | -0.39 | -0.98 | | -0.48 | -1.39 | -1.56 |
| Erk | Unigene12163_All | -1.48 | -0.86 | -1.60 | | -0.47 | -1.43 | -2.05 |
| Giα | Unigene13908_All | -2.07 | -1.47 | -2.32 | | -0.54 | -2.02 | -2.61 |
| PKA | Unigene14542_All | -1.47 | -0.76 | -1.76 | | -0.13 | -1.40 | -2.30 |
| nAChR | Unigene25202_All | -1.84 | -2.75 | -1.70 | | -3.35 | -1.13 | -1.36 |
| PKB/Akt | Unigene26509_All | -1.41 | -1.20 | -1.80 | | -0.48 | -1.09 | -1.34 |
| PKC | Unigene40306_All | -2.98 | -1.19 | -2.48 | | -0.60 | -1.64 | -2.37 |
| PKB/Akt | Unigene4933_All | -2.41 | -1.58 | -2.02 | | -0.53 | -1.42 | -2.94 |
| CaMK | Unigene5996_All | -2.11 | -1.44 | -2.25 | | -0.34 | -2.08 | -3.13 |
| PI3K | Unigene6308_All | -1.38 | -0.75 | -0.96 | | 0.01 | -0.56 | -0.83 |
| AC | Unigene64589_All | -2.59 | -1.03 | -2.09 | | -0.41 | -1.41 | -2.73 |
| AC | Unigene65078_All | -1.19 | -1.70 | -2.06 | | -1.05 | -1.05 | -3.16 |
| AC | Unigene67597_All | -1.81 | -1.27 | -1.53 | | -1.08 | -1.74 | -2.18 |
| JAK2 | Unigene6769_All | -1.13 | -1.00 | -1.14 | | -0.80 | -0.96 | -1.19 |
| PKB/Akt | Unigene69837_All | -2.54 | -1.45 | -2.80 | | -0.97 | -1.44 | -1.61 |
| AChE | Unigene71043_All | -2.50 | -2.23 | -2.97 | | -1.37 | -2.93 | -3.14 |
| ChAT | Unigene72957_All | -1.21 | -1.02 | -1.86 | | -0.45 | -1.83 | -2.74 |
| PI3K | Unigene9455_All | -1.78 | -1.67 | -2.48 | | -0.57 | -1.60 | -2.04 |
| AChE | CL11727.Contig1_All | -2.95 | -2.31 | -3.50 | | -0.44 | -1.37 | -2.03 |
| Kvτlkir2 | CL3275.Contig1_All | -0.97 | -1.64 | -0.67 | | -0.89 | -1.28 | -2.39 |
| AC | CL6134.Contig2_All | -2.50 | -3.02 | -2.16 | | -1.12 | -4.54 | -3.28 |
| Giα | CL7798.Contig1_All | -0.73 | -1.73 | -1.04 | | -0.82 | -1.10 | -1.07 |
| VGCC | Unigene12299_All | -0.87 | -1.54 | 0.79 | | -0.91 | 0.72 | 0.50 |
| AChE | Unigene14335_All | -0.36 | -1.12 | 0.27 | | 0.05 | 0.78 | -0.13 |
| AChE | Unigene2440_All | -7.66 | 3.86 | 2.13 | | 6.57 | -1.05 | -7.78 |
| nAChR | Unigene25204_All | -0.91 | -1.52 | -1.83 | | -1.37 | -0.64 | -1.07 |
| vACht | Unigene5066_All | -0.74 | -1.37 | -0.90 | | -1.06 | -0.66 | -0.86 |
| PKC | Unigene72446_All | -0.97 | -1.17 | -1.91 | | -0.39 | -1.44 | -1.91 |
| AChE | Unigene9281_All | -4.64 | 2.78 | 2.03 | | 3.58 | 0.68 | -2.43 |
| PLC | CL2281.Contig2_All | -0.92 | -1.00 | -1.49 | | -0.40 | -1.28 | -2.48 |
| VGCC | CL4555.Contig2_All | -8.68 | -1.34 | -9.66 | | -6.70 | -7.73 | -9.61 |
| PI3K | CL4556.Contig2_All | -0.99 | -0.70 | -1.73 | | -0.19 | -1.20 | -2.30 |
| PI3K | Unigene11052_All | -2.37 | -0.66 | -7.60 | | -0.57 | -2.05 | -2.11 |
| Ras | Unigene11311_All | -0.86 | -0.46 | -1.08 | | -0.54 | -0.88 | -0.98 |
| PKC | Unigene11828_All | -0.94 | -0.91 | -1.36 | | -0.75 | -1.71 | -1.64 |
| nAChR | Unigene25205_All | -1.44 | -1.15 | -1.92 | | -1.84 | -0.99 | -1.92 |
| nAChR | Unigene25206_All | -0.61 | -0.16 | -1.16 | | -0.35 | -0.46 | -1.29 |
| AChE | Unigene4842_All | -1.77 | -1.23 | -2.19 | | -0.65 | -1.42 | -2.11 |
| PKB/Akt | Unigene68579_All | -1.14 | -0.66 | -1.74 | | -0.77 | -1.51 | -2.43 |
| AC | Unigene9645_All | -0.51 | -0.79 | -0.81 | | -1.07 | -0.96 | -0.73 |
| PKC | CL11461.Contig1_All | -0.70 | -1.98 | -1.94 | | -1.20 | -2.86 | -2.01 |
| PI3K | CL4556.Contig1_All | -0.69 | -0.41 | -0.83 | | -0.58 | -1.35 | -1.98 |
| Giα | CL5187.Contig2_All | -0.88 | 0.42 | 0.41 | | -0.14 | -1.00 | -0.51 |
| VGCC | Unigene12233_All | 0.36 | 0.63 | 0.62 | | 0.39 | 1.39 | 1.33 |
| AChE | Unigene12727_All | -0.81 | -0.40 | -0.17 | | -0.53 | 1.68 | 1.32 |
| PKC | CL11461.Contig3_All | -2.27 | -2.17 | -2.46 | | -0.12 | -1.86 | -3.43 |
| PLC | CL11624.Contig1_All | -0.61 | -0.92 | -0.27 | | -0.82 | -0.53 | -1.99 |
| Kvτlkir2 | CL3275.Contig10_All | 0.17 | -0.11 | -0.63 | | -0.78 | -0.31 | -1.63 |
| Kvτlkir2 | CL3275.Contig11_All | 0.12 | 0.00 | -0.61 | | -0.96 | -0.33 | -1.67 |
| Kvτlkir2 | CL3275.Contig9_All | 0.04 | -0.22 | -0.60 | | -0.75 | -0.32 | -1.51 |
| AChE | CL8106.Contig2_All | -0.97 | -0.82 | -0.25 | | 0.33 | 0.07 | -1.77 |
| nAChR | CL928.Contig3_All | -1.50 | -1.08 | -1.24 | | -2.03 | -0.79 | -3.70 |
| Kvτlkir2 | Unigene10996_All | -0.21 | 0.02 | -0.60 | | -0.91 | -0.58 | -1.37 |
| nAChR | Unigene25200_All | -2.55 | -1.15 | -1.37 | | -1.70 | -1.24 | -3.43 |
| AC | Unigene32128_All | -1.25 | -1.20 | -0.83 | | -1.31 | -1.64 | -2.38 |
| AChE | Unigene5551_All | -0.70 | -0.37 | 0.36 | | -0.08 | -0.31 | -1.45 |
| AC | Unigene58654_All | -2.46 | -1.33 | -2.63 | | -2.58 | -2.38 | -3.69 |
| Giα | Unigene99_All | -0.43 | -0.21 | -0.84 | | -0.24 | -0.46 | -1.23 |
| **Dopaminergic synapse pathway** | |  |  |  | |  |  |  |
| CaMKII | CL12768.Contig1_All | -1.40 | -1.27 | -0.77 | | -0.24 | -0.74 | -1.11 |
| CaMKII | CL12768.Contig2_All | -1.73 | -1.47 | -1.69 | | -0.18 | -1.89 | -2.90 |
| CaMKII | CL12768.Contig3_All | -2.01 | -2.16 | -2.20 | | -0.40 | -1.39 | -2.38 |
| PLC | CL2281.Contig1_All | -1.14 | -0.82 | -1.53 | | -0.49 | -1.13 | -1.74 |
| Cav2.1/2.2 | CL2283.Contig1_All | -2.48 | -2.77 | -3.26 | | -2.93 | -2.57 | -2.51 |
| Clock | CL2554.Contig1_All | -2.57 | -1.45 | -2.01 | | -0.83 | -1.64 | -4.05 |
| PKA | CL32.Contig8_All | -1.16 | -0.65 | -1.41 | | -0.29 | -1.31 | -2.30 |
| MAPK | CL3431.Contig1_All | -1.25 | -0.77 | -1.17 | | -0.61 | -0.80 | -1.71 |
| MAPK | CL3431.Contig3_All | -1.53 | -0.92 | -1.37 | | -0.17 | -1.39 | -2.11 |
| DDC | CL3484.Contig2_All | -1.11 | 0.39 | -0.61 | | 0.10 | -1.06 | 0.47 |
| CaMKII | CL5029.Contig1_All | -1.33 | -1.30 | -1.70 | | 0.11 | -1.30 | -1.78 |
| AMPAr | CL66.Contig4_All | -2.48 | -0.28 | -1.27 | | -0.48 | -0.64 | -2.43 |
| AMPAr | CL66.Contig6_All | -1.59 | -0.59 | -1.78 | | -0.37 | -1.86 | -3.00 |
| CaM | CL7465.Contig1_All | -1.51 | 1.13 | 3.44 | | 0.94 | 0.73 | 1.32 |
| Gi/o | CL7798.Contig2_All | -1.09 | -1.63 | -0.62 | | -1.26 | -0.92 | -0.96 |
| NMDAr | CL8380.Contig1_All | -3.27 | -2.08 | -0.95 | | -1.70 | -5.99 | -2.61 |
| DDC | CL8446.Contig3_All | -2.37 | -1.73 | -1.68 | | 0.02 | -2.64 | -2.64 |
| CREB | CL8614.Contig1_All | -1.24 | -0.64 | -1.43 | | -0.63 | -1.53 | -1.64 |
| CREB | CL8614.Contig2_All | -1.14 | -1.48 | -0.75 | | -0.99 | -0.76 | -0.58 |
| Gi/o | Unigene1049_All | -1.10 | -0.39 | -0.98 | | -0.48 | -1.39 | -1.56 |
| Gs/olf | Unigene10560_All | -1.54 | -2.27 | -2.10 | | -0.44 | -0.82 | -1.16 |
| PP2A | Unigene1239_All | -1.69 | -0.81 | -1.74 | | -0.48 | -1.22 | -2.09 |
| Clock | Unigene1266_All | -2.01 | -1.67 | -3.44 | | -0.72 | -2.54 | -2.76 |
| Kinesin | Unigene13614_All | -2.17 | -1.43 | -2.09 | | -0.35 | -1.90 | -2.72 |
| Gi/o | Unigene13908_All | -2.07 | -1.47 | -2.32 | | -0.54 | -2.02 | -2.61 |
| PKA | Unigene14542_All | -1.47 | -0.76 | -1.76 | | -0.13 | -1.40 | -2.30 |
| Akt | Unigene26509_All | -1.41 | -1.20 | -1.80 | | -0.48 | -1.09 | -1.34 |
| PP2A | Unigene3105_All | -1.67 | -1.14 | -2.05 | | -0.32 | -1.57 | -2.05 |
| PKC | Unigene40306_All | -2.98 | -1.19 | -2.48 | | -0.60 | -1.64 | -2.37 |
| Akt | Unigene4933_All | -2.41 | -1.58 | -2.02 | | -0.53 | -1.42 | -2.94 |
| CREB | Unigene6308_All | -1.38 | -0.75 | -0.96 | | 0.01 | -0.56 | -0.83 |
| VMAT | Unigene66086_All | -1.73 | -0.80 | -1.46 | | -0.43 | -1.50 | -3.17 |
| Akt | Unigene69837_All | -2.54 | -1.45 | -2.80 | | -0.97 | -1.44 | -1.61 |
| VMAT | Unigene72668_All | -1.52 | -0.84 | -1.55 | | -0.53 | -2.00 | -2.62 |
| Clock | Unigene7682_All | -1.13 | -0.72 | -1.30 | | -0.81 | -1.53 | -1.76 |
| PP2A | CL2228.Contig3_All | -0.63 | -1.22 | -1.03 | | -0.33 | -0.49 | -1.42 |
| CaM | CL7465.Contig2_All | 0.92 | 1.79 | -1.26 | | -2.44 | -2.74 | -1.11 |
| Gi/o | CL7798.Contig1_All | -0.73 | -1.73 | -1.04 | | -0.82 | -1.10 | -1.07 |
| NMDAr | CL8380.Contig2_All | -1.59 | -2.45 | -1.95 | | -2.58 | -2.51 | -4.06 |
| CREB | CL8614.Contig3_All | -1.22 | -1.77 | -0.85 | | -1.03 | -0.99 | -2.17 |
| MAO | Unigene11212_All | 0.44 | -3.27 | 0.37 | | 5.31 | 0.80 | 0.63 |
| VGGC | Unigene12299_All | -0.87 | -1.54 | 0.79 | | -0.91 | 0.72 | 0.50 |
| CaM | Unigene1278_All | -0.17 | 1.90 | 3.22 | | -0.22 | 1.91 | 3.55 |
| PP2A | Unigene13942_All | 0.17 | 1.26 | 0.60 | | -0.06 | -0.81 | -0.36 |
| PKC | Unigene72446_All | -0.97 | -1.17 | -1.91 | | -0.39 | -1.44 | -1.91 |
| CREB | Unigene9387_All | -0.85 | -1.35 | -1.37 | | -0.77 | -0.59 | -1.08 |
| CaM | Unigene9663_All | -0.70 | 1.03 | 2.38 | | 0.09 | 2.96 | 2.18 |
| CaM | Unigene96_All | -0.55 | -1.11 | -0.63 | | -0.50 | -0.42 | -0.89 |
| PLC | CL2281.Contig2_All | -0.92 | -1.00 | -1.49 | | -0.40 | -1.28 | -2.48 |
| MAPK | CL3431.Contig2_All | -0.94 | -0.77 | -1.06 | | -0.27 | -1.10 | -1.62 |
| GSK-3 | CL4139.Contig2_All | -0.56 | -0.93 | -1.10 | | -0.95 | -0.51 | -0.96 |
| VGGC | CL4555.Contig2_All | -8.68 | -1.34 | -9.66 | | -6.70 | -7.73 | -9.61 |
| AMPAr | CL66.Contig5_All | -1.47 | -0.70 | -2.09 | | -0.18 | -0.96 | -2.43 |
| PKC | Unigene11828_All | -0.94 | -0.91 | -1.36 | | -0.75 | -1.71 | -1.64 |
| AMPAr | Unigene24013_All | -0.89 | -0.35 | -1.01 | | -0.30 | -1.12 | -1.67 |
| Akt | Unigene68579_All | -1.14 | -0.66 | -1.74 | | -0.77 | -1.51 | -2.43 |
| AMPAr | Unigene71686_All | -0.72 | -0.63 | -1.06 | | 0.11 | -0.79 | -2.71 |
| AMPAr | Unigene72065_All | -0.36 | -0.67 | -1.50 | | -0.16 | -1.11 | -3.63 |
| DDC | CL2735.Contig4_All | -0.89 | -0.51 | -0.88 | | -1.03 | -0.43 | -0.56 |
| PP-1 | CL6805.Contig3_All | -0.94 | -0.84 | -0.69 | | -1.51 | -0.60 | 0.11 |
| PKC | CL11461.Contig1_All | -0.70 | -1.98 | -1.94 | | -1.20 | -2.86 | -2.01 |
| Gi/o | CL5187.Contig2_All | -0.88 | 0.42 | 0.41 | | -0.14 | -1.00 | -0.51 |
| CaM | CL6746.Contig1_All | -0.47 | 0.09 | -0.51 | | -0.02 | -1.70 | -0.76 |
| MAO | Unigene7582_All | 0.26 | 0.26 | 0.91 | | -0.68 | -1.00 | -0.24 |
| VMAT | Unigene8858_All | -0.10 | 0.88 | -0.16 | | -0.48 | -2.04 | -1.25 |
| MAO | Unigene9218_All | -0.98 | -0.16 | 0.53 | | -0.79 | -1.62 | -0.78 |
| PKC | CL11461.Contig3_All | -2.27 | -2.17 | -2.46 | | -0.12 | -1.86 | -3.43 |
| PLC | CL11624.Contig1_All | -0.61 | -0.92 | -0.27 | | -0.82 | -0.53 | -1.99 |
| TH | CL11994.Contig1_All | -0.19 | -0.43 | -0.36 | | -0.93 | 0.48 | 1.30 |
| PP2A | CL1395.Contig1_All | -0.62 | -0.09 | -0.67 | | 0.11 | -0.84 | -1.04 |
| BMALL | CL187.Contig1_All | -3.50 | -1.16 | -1.72 | | -1.44 | -2.97 | -2.63 |
| BMALL | CL187.Contig2_All | -1.27 | -1.21 | -1.12 | | -2.29 | -2.12 | -3.43 |
| BMALL | CL187.Contig3_All | -1.67 | -0.87 | -1.20 | | -0.79 | -1.91 | -1.88 |
| PP2A | CL2228.Contig1_All | -1.78 | -0.66 | -1.20 | | 0.62 | -1.55 | -2.81 |
| AMPAr | CL66.Contig2_All | -0.39 | -0.30 | -0.98 | | -0.32 | -0.73 | -1.48 |
| AMPAr | CL66.Contig3_All | -0.33 | -0.48 | -0.62 | | -0.26 | -0.63 | -1.12 |
| PP2A | CL7051.Contig1_All | -1.16 | -1.46 | -1.04 | | -0.53 | 0.68 | -8.21 |
| PP2A | CL7051.Contig2_All | -1.04 | -1.08 | -1.63 | | 0.15 | 0.43 | -8.04 |
| CaM | CL7675.Contig2_All | 0.09 | -0.40 | -0.39 | | -0.18 | -1.25 | -2.32 |
| GSK-3 | Unigene14019_All | -1.11 | -0.46 | -0.71 | | -0.99 | -1.14 | -1.53 |
| BMALL | Unigene31613_All | 0.00 | -1.66 | -9.75 | | -1.12 | 0.95 | -10.80 |
| AMPAr | Unigene31894_All | -1.78 | -0.42 | -1.72 | | -1.88 | -1.75 | -2.69 |
| PP-1 | Unigene4739_All | -0.60 | -0.71 | -0.71 | | -0.31 | -0.74 | -1.25 |
| PP2A | Unigene68161_All | -0.83 | -0.85 | -1.19 | | -1.06 | -0.99 | -2.47 |
| AMPAr | Unigene71685_All | -0.80 | -0.08 | -1.10 | | 0.24 | -1.22 | -2.69 |
| DAT | Unigene725_All | -0.41 | -0.56 | -0.37 | | -0.29 | -0.65 | -1.11 |
| DAT | Unigene80245_All | -1.34 | -0.65 | -1.31 | | -0.60 | -1.78 | -2.49 |
| Gi/o | Unigene99_All | -0.43 | -0.21 | -0.84 | | -0.24 | -0.46 | -1.23 |
| **GABAergic synapse pathway** | |  |  |  | |  |  |  |
| AC | CL12536.Contig1_All | -1.52 | -1.25 | -2.49 | | -1.78 | -1.27 | -1.11 |
| AC | CL12536.Contig2_All | -1.58 | -1.58 | -1.48 | | -1.54 | -1.12 | -1.05 |
| GABAA | CL1881.Contig2_All | -1.02 | -0.22 | -0.44 | | -0.86 | -0.91 | -1.13 |
| VGCC | CL2283.Contig1_All | -2.48 | -2.77 | -3.26 | | -2.93 | -2.57 | -2.51 |
| PKA | CL32.Contig8_All | -1.16 | -0.65 | -1.41 | | -0.29 | -1.31 | -2.30 |
| GABAB | CL4168.Contig1_All | -3.50 | -1.70 | -1.85 | | 0.05 | -1.32 | -1.11 |
| AC | CL5341.Contig1_All | -1.38 | -1.33 | -2.23 | | -1.28 | -1.54 | -2.65 |
| GABAA | CL548.Contig1_All | -5.09 | -3.00 | -5.81 | | -2.12 | -7.69 | -11.29 |
| PRIP | CL5891.Contig1_All | -1.34 | -1.32 | -1.94 | | -0.78 | -1.14 | -1.48 |
| AC | CL6305.Contig1_All | -7.51 | -2.51 | -2.18 | | 0.05 | -1.37 | -3.35 |
| AC | CL6305.Contig2_All | -2.49 | -1.87 | -3.31 | | -2.93 | -0.40 | -3.66 |
| GLS | CL656.Contig2_All | -1.12 | -0.30 | -1.21 | | 0.09 | -1.53 | -2.20 |
| GABAA | CL6923.Contig2_All | -1.66 | -1.74 | -1.57 | | -0.25 | -0.52 | -1.36 |
| Gi/o | CL7798.Contig2_All | -1.09 | -1.63 | -0.62 | | -1.26 | -0.92 | -0.96 |
| Gi/o | Unigene1049_All | -1.10 | -0.39 | -0.98 | | -0.48 | -1.39 | -1.56 |
| NSF | Unigene10660_All | -1.61 | -0.91 | -1.54 | | -0.06 | -1.85 | -2.62 |
| Gi/o | Unigene13908_All | -2.07 | -1.47 | -2.32 | | -0.54 | -2.02 | -2.61 |
| PKA | Unigene14542_All | -1.47 | -0.76 | -1.76 | | -0.13 | -1.40 | -2.30 |
| GAT | Unigene2477_All | -1.75 | -1.58 | -1.87 | | -0.45 | -1.40 | -2.43 |
| Gephyrin | Unigene306_All | -1.01 | -0.51 | -1.28 | | -0.19 | -1.50 | -2.01 |
| PKC | Unigene40306_All | -2.98 | -1.19 | -2.48 | | -0.60 | -1.64 | -2.37 |
| VGAT | Unigene4955_All | -2.36 | -3.65 | -3.73 | | -1.94 | -2.31 | -2.69 |
| GABAB | Unigene56070_All | -3.00 | -1.52 | -2.85 | | -1.44 | -1.32 | -4.08 |
| NSF | Unigene5790_All | -1.30 | -0.87 | -1.28 | | -0.29 | -1.26 | -1.73 |
| AC | Unigene64589_All | -2.59 | -1.03 | -2.09 | | -0.41 | -1.41 | -2.73 |
| AC | Unigene65078_All | -1.19 | -1.70 | -2.06 | | -1.05 | -1.05 | -3.16 |
| AC | Unigene67597_All | -1.81 | -1.27 | -1.53 | | -1.08 | -1.74 | -2.18 |
| VGAT | Unigene76468_All | -4.50 | -2.90 | -3.39 | | -0.02 | -1.54 | -4.43 |
| GAT | Unigene80246_All | -2.21 | -0.68 | -1.60 | | -0.04 | -1.31 | -3.04 |
| AC | CL6134.Contig2_All | -2.50 | -3.02 | -2.16 | | -1.12 | -4.54 | -3.28 |
| Gi/o | CL7798.Contig1_All | -0.73 | -1.73 | -1.04 | | -0.82 | -1.10 | -1.07 |
| GABAB | CL9773.Contig1_All | -1.92 | -2.70 | -3.67 | | -3.82 | -1.05 | -4.32 |
| VGCC | Unigene12299_All | -0.87 | -1.54 | 0.79 | | -0.91 | 0.72 | 0.50 |
| GLS | Unigene5837_All | -0.05 | 4.68 | -1.04 | | -3.57 | 0.80 | 0.74 |
| PKC | Unigene72446_All | -0.97 | -1.17 | -1.91 | | -0.39 | -1.44 | -1.91 |
| GABAB | Unigene78823_All | -0.71 | -1.08 | -0.85 | | -0.78 | -0.50 | -1.11 |
| GAT | Unigene80247_All | -1.04 | -1.14 | -2.74 | | -0.32 | -0.79 | -3.14 |
| NSF | Unigene8621_All | -0.99 | -1.05 | -1.75 | | 0.19 | -1.36 | -3.28 |
| GLS | CL11512.Contig4_All | 0.18 | 0.46 | 1.11 | | -0.18 | -0.89 | -0.98 |
| VGCC | CL4555.Contig2_All | -8.68 | -1.34 | -9.66 | | -6.70 | -7.73 | -9.61 |
| VGAT | CL6277.Contig1_All | -3.21 | -0.69 | -2.69 | | -1.23 | -3.05 | -2.77 |
| GLS | CL656.Contig3_All | -0.39 | 0.39 | -1.54 | | -0.54 | -2.07 | -3.84 |
| PKC | Unigene11828_All | -0.94 | -0.91 | -1.36 | | -0.75 | -1.71 | -1.64 |
| GABAA | Unigene57709_All | -1.55 | -1.71 | -2.90 | | -1.38 | -1.17 | -10.37 |
| GABAA | Unigene83870_All | -1.04 | -2.40 | -10.59 | | -0.93 | -2.05 | -3.43 |
| VGAT | Unigene27251_All | -0.19 | -0.41 | -0.92 | | -1.63 | -0.19 | 0.01 |
| VGAT | Unigene3045_All | -0.80 | -0.18 | -0.62 | | -1.19 | 0.26 | 0.25 |
| GABAA | Unigene3712_All | 0.53 | -0.78 | 0.63 | | -1.27 | -2.07 | -2.01 |
| AC | Unigene9645_All | -0.51 | -0.79 | -0.81 | | -1.07 | -0.96 | -0.73 |
| GS | CL10431.Contig2_All | -0.42 | -0.26 | -0.42 | | -0.15 | -1.01 | -1.01 |
| PKC | CL11461.Contig1_All | -0.70 | -1.98 | -1.94 | | -1.20 | -2.86 | -2.01 |
| Gi/o | CL5187.Contig2_All | -0.88 | 0.42 | 0.41 | | -0.14 | -1.00 | -0.51 |
| GAD | CL5463.Contig1_All | -0.01 | 0.32 | -0.23 | | -0.83 | -1.50 | -1.56 |
| GAD | Unigene1151_All | -0.21 | -0.36 | 0.28 | | -0.91 | 2.58 | 2.97 |
| VGCC | Unigene12233_All | 0.36 | 0.63 | 0.62 | | 0.39 | 1.39 | 1.33 |
| VGAT | Unigene26255_All | -1.53 | -0.99 | -1.40 | | -0.97 | -2.49 | -1.72 |
| PKC | CL11461.Contig3_All | -2.27 | -2.17 | -2.46 | | -0.12 | -1.86 | -3.43 |
| GLS | CL656.Contig1_All | 0.22 | 0.04 | -0.18 | | -0.49 | -0.67 | -1.17 |
| VGAT | CL9007.Contig1_All | -0.92 | -0.15 | -0.99 | | -0.10 | -0.54 | -1.06 |
| AC | Unigene32128_All | -1.25 | -1.20 | -0.83 | | -1.31 | -1.64 | -2.38 |
| GABAA | Unigene40418_All | -3.95 | -1.61 | -3.21 | | -1.93 | -2.37 | -4.43 |
| AC | Unigene58654_All | -2.46 | -1.33 | -2.63 | | -2.58 | -2.38 | -3.69 |
| GAT | Unigene7275_All | -0.45 | -0.41 | -0.47 | | -0.18 | -0.62 | -1.02 |
| Gi/o | Unigene99_All | -0.43 | -0.21 | -0.84 | | -0.24 | -0.46 | -1.23 |
| **Glutamatergic synapse pathway** | |  |  |  | |  |  |  |
| mGluR2/3 | CL11587.Contig1_All | -1.58 | -2.02 | -1.82 | | -2.36 | -1.10 | -0.44 |
| AC | CL12536.Contig1_All | -1.52 | -1.25 | -2.49 | | -1.78 | -1.27 | -1.11 |
| AC | CL12536.Contig2_All | -1.58 | -1.58 | -1.48 | | -1.54 | -1.12 | -1.05 |
| SHANK | CL13034.Contig1_All | -1.28 | -0.89 | -1.81 | | -0.29 | -1.39 | -2.02 |
| PLC | CL2281.Contig1_All | -1.14 | -0.82 | -1.53 | | -0.49 | -1.13 | -1.74 |
| VGCC | CL2283.Contig1_All | -2.48 | -2.77 | -3.26 | | -2.93 | -2.57 | -2.51 |
| PKA | CL32.Contig8_All | -1.16 | -0.65 | -1.41 | | -0.29 | -1.31 | -2.30 |
| nGluR2/3 | CL4447.Contig2_All | -1.61 | -1.96 | -2.30 | | -2.59 | -1.28 | -1.32 |
| AC | CL5341.Contig1_All | -1.38 | -1.33 | -2.23 | | -1.28 | -1.54 | -2.65 |
| KA | CL5546.Contig3_All | -1.10 | -1.58 | -2.67 | | -1.27 | -2.11 | -2.60 |
| KA | CL5546.Contig4_All | -2.08 | -1.11 | -2.12 | | -0.12 | -1.75 | -2.76 |
| AC | CL6305.Contig1_All | -7.51 | -2.51 | -2.18 | | 0.05 | -1.37 | -3.35 |
| AC | CL6305.Contig2_All | -2.49 | -1.87 | -3.31 | | -2.93 | -0.40 | -3.66 |
| GLS | CL656.Contig2_All | -1.12 | -0.30 | -1.21 | | 0.09 | -1.53 | -2.20 |
| AMPA | CL66.Contig4_All | -2.48 | -0.28 | -1.27 | | -0.48 | -0.64 | -2.43 |
| AMPA | CL66.Contig6_All | -1.59 | -0.59 | -1.78 | | -0.37 | -1.86 | -3.00 |
| mGluR2/3 | CL7570.Contig1_All | -1.21 | -0.63 | -1.51 | | -1.38 | -1.33 | 0.01 |
| Gi/o | CL7798.Contig2_All | -1.09 | -1.63 | -0.62 | | -1.26 | -0.92 | -0.96 |
| NMDAR | CL8380.Contig1_All | -3.27 | -2.08 | -0.95 | | -1.70 | -5.99 | -2.61 |
| Gi/o | Unigene1049_All | -1.10 | -0.39 | -0.98 | | -0.48 | -1.39 | -1.56 |
| Gs | Unigene10560_All | -1.54 | -2.27 | -2.10 | | -0.44 | -0.82 | -1.16 |
| ERK1/2 | Unigene12163_All | -1.48 | -0.86 | -1.60 | | -0.47 | -1.43 | -2.05 |
| PLA2 | Unigene12178_All | -1.07 | 0.11 | -0.08 | | -0.32 | -0.89 | -0.21 |
| Gi/o | Unigene13908_All | -2.07 | -1.47 | -2.32 | | -0.54 | -2.02 | -2.61 |
| PKA | Unigene14542_All | -1.47 | -0.76 | -1.76 | | -0.13 | -1.40 | -2.30 |
| GKAP | Unigene1774_All | -1.99 | -1.57 | -2.64 | | -0.71 | -1.37 | -1.42 |
| mGluR2/3 | Unigene2679_All | -1.24 | -1.74 | -1.53 | | -1.72 | -1.37 | -1.13 |
| SHANK | Unigene3396_All | -1.11 | -1.36 | -1.89 | | -1.39 | -1.10 | -1.48 |
| PKC | Unigene40306_All | -2.98 | -1.19 | -2.48 | | -0.60 | -1.64 | -2.37 |
| NMDAR | Unigene63984_All | -1.70 | -1.49 | -2.66 | | -0.29 | -1.86 | -3.04 |
| AC | Unigene64589_All | -2.59 | -1.03 | -2.09 | | -0.41 | -1.41 | -2.73 |
| AC | Unigene65078_All | -1.19 | -1.70 | -2.06 | | -1.05 | -1.05 | -3.16 |
| AC | Unigene67597_All | -1.81 | -1.27 | -1.53 | | -1.08 | -1.74 | -2.18 |
| mGluR2/3 | Unigene78451_All | 9.71 | -0.56 | 9.33 | | -9.15 | 8.57 | 8.48 |
| mGluR2/3 | Unigene7991_All | -1.29 | -1.44 | -1.39 | | -1.31 | -0.92 | -0.55 |
| mGluR2/3 | Unigene8004_All | -2.55 | 0.30 | -1.95 | | -1.99 | -3.05 | -0.03 |
| mGlu2/3 | CL11760.Contig1_All | -1.73 | -3.25 | -2.21 | | -1.84 | -0.76 | -1.40 |
| mGluR2/3 | CL4438.Contig2_All | -0.93 | -2.50 | -1.12 | | -1.38 | -0.80 | -1.22 |
| AC | CL6134.Contig2_All | -2.50 | -3.02 | -2.16 | | -1.12 | -4.54 | -3.28 |
| Gi/o | CL7798.Contig1_All | -0.73 | -1.73 | -1.04 | | -0.82 | -1.10 | -1.07 |
| NMDAR | CL8380.Contig2_All | -1.59 | -2.45 | -1.95 | | -2.58 | -2.51 | -4.06 |
| Vglut | CL9844.Contig2_All | 0.21 | -1.19 | 0.10 | | -0.08 | 1.21 | 0.92 |
| VGCC | Unigene12299_All | -0.87 | -1.54 | 0.79 | | -0.91 | 0.72 | 0.50 |
| mGluR1/5 | Unigene12739_All | -0.80 | -1.07 | -0.69 | | -0.59 | -0.69 | -1.09 |
| mGluR2/3 | Unigene15335_All | -1.20 | -2.61 | -3.16 | | -0.60 | -0.51 | -2.57 |
| GLS | Unigene5837_All | -0.05 | 4.68 | -1.04 | | -3.57 | 0.80 | 0.74 |
| SHANK | Unigene69218_All | -2.16 | -2.12 | -2.04 | | 0.10 | -0.05 | -3.11 |
| PKC | Unigene72446_All | -0.97 | -1.17 | -1.91 | | -0.39 | -1.44 | -1.91 |
| PLA2 | Unigene80923_All | -2.21 | -2.14 | -1.56 | | 1.76 | -2.86 | -2.49 |
| GLS | CL11512.Contig4_All | 0.18 | 0.46 | 1.11 | | -0.18 | -0.89 | -0.98 |
| SHANK | CL13515.Contig2_All | -0.53 | -0.85 | -1.04 | | -0.72 | -0.71 | -1.45 |
| PLC | CL2281.Contig2_All | -0.92 | -1.00 | -1.49 | | -0.40 | -1.28 | -2.48 |
| VGCC | CL4555.Contig2_All | -8.68 | -1.34 | -9.66 | | -6.70 | -7.73 | -9.61 |
| KA | CL5546.Contig2_All | -0.31 | -0.85 | -1.31 | | -0.65 | -1.84 | -1.43 |
| GLS | CL656.Contig3_All | -0.39 | 0.39 | -1.54 | | -0.54 | -2.07 | -3.84 |
| AMPA | CL66.Contig5_All | -1.47 | -0.70 | -2.09 | | -0.18 | -0.96 | -2.43 |
| mGluR2/3 | CL6636.Contig2_All | -1.04 | -2.98 | -2.57 | | -1.70 | -1.37 | -0.73 |
| PKC | Unigene11828_All | -0.94 | -0.91 | -1.36 | | -0.75 | -1.71 | -1.64 |
| AMPA | Unigene24013_All | -0.89 | -0.35 | -1.01 | | -0.30 | -1.12 | -1.67 |
| AMPA | Unigene71686_All | -0.72 | -0.63 | -1.06 | | 0.11 | -0.79 | -2.71 |
| AMPA | Unigene72065_All | -0.36 | -0.67 | -1.50 | | -0.16 | -1.11 | -3.63 |
| mGluR2/3 | CL5213.Contig1_All | 0.32 | -0.53 | -0.56 | | -1.40 | 0.59 | -2.62 |
| Homer | Unigene4320_All | -0.55 | 0.01 | -0.88 | | -1.50 | -0.83 | -1.06 |
| AC | Unigene9645_All | -0.51 | -0.79 | -0.81 | | -1.07 | -0.96 | -0.73 |
| CLNS | CL10431.Contig2_All | -0.42 | -0.26 | -0.42 | | -0.15 | -1.01 | -1.01 |
| PKC | CL11461.Contig1_All | -0.70 | -1.98 | -1.94 | | -1.20 | -2.86 | -2.01 |
| Gi/o | CL5187.Contig2_All | -0.88 | 0.42 | 0.41 | | -0.14 | -1.00 | -0.51 |
| PKC | CL11461.Contig3_All | -2.27 | -2.17 | -2.46 | | -0.12 | -1.86 | -3.43 |
| PLC | CL11624.Contig1_All | -0.61 | -0.92 | -0.27 | | -0.82 | -0.53 | -1.99 |
| SHANK | CL13515.Contig1_All | -0.73 | -0.58 | -0.95 | | -0.43 | -0.78 | -1.19 |
| GLS | CL656.Contig1_All | 0.22 | 0.04 | -0.18 | | -0.49 | -0.67 | -1.17 |
| AMPA | CL66.Contig2_All | -0.39 | -0.30 | -0.98 | | -0.32 | -0.73 | -1.48 |
| AMPA | CL66.Contig3_All | -0.33 | -0.48 | -0.62 | | -0.26 | -0.63 | -1.12 |
| AMPA | Unigene31894_All | -1.78 | -0.42 | -1.72 | | -1.88 | -1.75 | -2.69 |
| AC | Unigene32128_All | -1.25 | -1.20 | -0.83 | | -1.31 | -1.64 | -2.38 |
| KA | Unigene3234_All | -0.42 | -0.52 | -0.36 | | -0.31 | -0.56 | -1.02 |
| AC | Unigene58654_All | -2.46 | -1.33 | -2.63 | | -2.58 | -2.38 | -3.69 |
| Homer | Unigene68721_All | -0.74 | -0.23 | -1.17 | | -1.32 | -1.88 | -3.25 |
| AMPA | Unigene71685_All | -0.80 | -0.08 | -1.10 | | 0.24 | -1.22 | -2.69 |
| Gi/o | Unigene99_All | -0.43 | -0.21 | -0.84 | | -0.24 | -0.46 | -1.23 |
| **Long-term depression pathway** | |  |  |  | |  |  |  |
| GUCY | CL10550.Contig1_All | -1.96 | -1.06 | -1.56 | | -0.58 | -2.32 | -3.01 |
| PLC | CL2281.Contig1_All | -1.14 | -0.82 | -1.53 | | -0.49 | -1.13 | -1.74 |
| VGCC | CL2283.Contig1_All | -2.48 | -2.77 | -3.26 | | -2.93 | -2.57 | -2.51 |
| Raf | CL3009.Contig1_All | -1.06 | -0.45 | -0.79 | | -1.14 | -1.27 | -1.68 |
| AMRPA | CL66.Contig4_All | -2.48 | -0.28 | -1.27 | | -0.48 | -0.64 | -2.43 |
| AMRPA | CL66.Contig6_All | -1.59 | -0.59 | -1.78 | | -0.37 | -1.86 | -3.00 |
| Raf | CL6905.Contig2_All | -1.87 | -2.52 | -2.53 | | -1.64 | -1.71 | -2.73 |
| G | Unigene1049_All | -1.10 | -0.39 | -0.98 | | -0.48 | -1.39 | -1.56 |
| G | Unigene10560_All | -1.54 | -2.27 | -2.10 | | -0.44 | -0.82 | -1.16 |
| ERK1/2 | Unigene12163_All | -1.48 | -0.86 | -1.60 | | -0.47 | -1.43 | -2.05 |
| PLA2 | Unigene12178_All | -1.07 | 0.11 | -0.08 | | -0.32 | -0.89 | -0.21 |
| PKG | Unigene12649_All | -1.33 | -0.63 | -1.47 | | -0.66 | -1.85 | -2.76 |
| G | Unigene13012_All | 3.60 | 9.96 | 1.42 | | 0.54 | -3.43 | -1.11 |
| PKC | Unigene40306_All | -2.98 | -1.19 | -2.48 | | -0.60 | -1.64 | -2.37 |
| G | Unigene41131_All | 10.38 | 2.45 | -10.46 | | 0.08 | -4.51 | 0.00 |
| IGF1R | Unigene6500_All | -1.14 | -1.13 | -1.20 | | -0.37 | -0.77 | -0.68 |
| GUCY | Unigene70403_All | -2.07 | -1.49 | -2.26 | | -1.07 | -2.07 | -3.24 |
| Raf | Unigene78202_All | 1.09 | 0.60 | 0.27 | | 0.26 | 0.67 | -0.36 |
| Raf | Unigene7991_All | -1.29 | -1.44 | -1.39 | | -1.31 | -0.92 | -0.55 |
| PKG | Unigene9223_All | -1.06 | -0.67 | -1.21 | | -0.58 | -1.68 | -1.93 |
| G | Unigene9442_All | -1.29 | -1.19 | -1.29 | | -0.62 | -1.23 | -1.17 |
| Raf | CL2444.Contig1_All | -0.32 | -1.23 | -0.97 | | -0.66 | -0.23 | -0.47 |
| Raf | Unigene11367_All | -0.56 | -3.38 | 0.39 | | -1.35 | -1.75 | -4.69 |
| VGCC | Unigene12299_All | -0.87 | -1.54 | 0.79 | | -0.91 | 0.72 | 0.50 |
| mGluR1 | Unigene12739_All | -0.80 | -1.07 | -0.69 | | -0.59 | -0.69 | -1.09 |
| Raf | Unigene29393_All | 0.08 | -2.12 | -0.34 | | -0.69 | -0.62 | -0.32 |
| IGF1R | Unigene63550_All | -0.94 | -2.10 | -3.04 | | -1.28 | -0.29 | -1.24 |
| PKC | Unigene72446_All | -0.97 | -1.17 | -1.91 | | -0.39 | -1.44 | -1.91 |
| PLA2 | Unigene80923_All | -2.21 | -2.14 | -1.56 | | 1.76 | -2.86 | -2.49 |
| CRFR1 | CL13697.Contig3_All | -1.73 | -0.60 | -2.92 | | -1.62 | -1.86 | -3.69 |
| CRFR1 | CL2194.Contig2_All | -0.39 | -0.17 | -1.04 | | -0.93 | 0.10 | -0.64 |
| PLC | CL2281.Contig2_All | -0.92 | -1.00 | -1.49 | | -0.40 | -1.28 | -2.48 |
| Raf | CL4034.Contig4_All | -1.27 | -0.42 | -2.71 | | -1.98 | -3.14 | -2.59 |
| VGCC | CL4555.Contig2_All | -8.68 | -1.34 | -9.66 | | -6.70 | -7.73 | -9.61 |
| AMRPA | CL66.Contig5_All | -1.47 | -0.70 | -2.09 | | -0.18 | -0.96 | -2.43 |
| Raf | CL6948.Contig1_All | -2.18 | -0.21 | -2.36 | | -1.31 | -1.86 | -0.88 |
| Raf | CL8054.Contig1_All | -0.53 | 0.51 | -3.44 | | -1.12 | -0.27 | 0.35 |
| Ras | Unigene11311_All | -0.86 | -0.46 | -1.08 | | -0.54 | -0.88 | -0.98 |
| PKC | Unigene11828_All | -0.94 | -0.91 | -1.36 | | -0.75 | -1.71 | -1.64 |
| AMRPA | Unigene24013_All | -0.89 | -0.35 | -1.01 | | -0.30 | -1.12 | -1.67 |
| CRFR1 | Unigene26868_All | -1.78 | -1.44 | -2.92 | | 0.30 | -1.05 | -1.03 |
| AMRPA | Unigene71686_All | -0.72 | -0.63 | -1.06 | | 0.11 | -0.79 | -2.71 |
| AMRPA | Unigene72065_All | -0.36 | -0.67 | -1.50 | | -0.16 | -1.11 | -3.63 |
| GUCY | Unigene8418_All | -0.94 | -0.75 | -1.08 | | -0.63 | -1.05 | -1.67 |
| PKC | CL11461.Contig1_All | -0.70 | -1.98 | -1.94 | | -1.20 | -2.86 | -2.01 |
| GUCY | CL12822.Contig2_All | -0.68 | -0.94 | -0.95 | | -0.83 | -1.09 | -1.29 |
| PKG | CL3262.Contig1_All | -0.51 | -0.25 | -0.47 | | -0.44 | -1.31 | -1.66 |
| PKG | CL3262.Contig2_All | -0.58 | -0.29 | -0.84 | | -0.72 | -1.16 | -1.26 |
| Raf | CL4304.Contig1_All | -0.21 | 0.07 | -0.67 | | -1.12 | -1.25 | -0.40 |
| PKC | CL11461.Contig3_All | -2.27 | -2.17 | -2.46 | | -0.12 | -1.86 | -3.43 |
| PLC | CL11624.Contig1_All | -0.61 | -0.92 | -0.27 | | -0.82 | -0.53 | -1.99 |
| CRFR1 | CL13697.Contig1_All | -0.91 | -0.38 | -1.24 | | 0.05 | -1.73 | -3.28 |
| PP2A | CL1395.Contig1_All | -0.62 | -0.09 | -0.67 | | 0.11 | -0.84 | -1.04 |
| CRFR1 | CL14054.Contig1_All | 0.09 | 0.00 | 0.00 | | -0.70 | -0.09 | -1.58 |
| AMRPA | CL66.Contig2_All | -0.39 | -0.30 | -0.98 | | -0.32 | -0.73 | -1.48 |
| AMRPA | CL66.Contig3_All | -0.33 | -0.48 | -0.62 | | -0.26 | -0.63 | -1.12 |
| AMRPA | Unigene31894_All | -1.78 | -0.42 | -1.72 | | -1.88 | -1.75 | -2.69 |
| AMRPA | Unigene71685_All | -0.80 | -0.08 | -1.10 | | 0.24 | -1.22 | -2.69 |
| G | Unigene99_All | -0.43 | -0.21 | -0.84 | | -0.24 | -0.46 | -1.23 |
| **Long-term potentiation pathway** | |  |  |  | |  |  |  |
| AC1/8 | CL12536.Contig1_All | -1.52 | -1.25 | -2.49 | | -1.78 | -1.27 | -1.11 |
| AC1/8 | CL12536.Contig2_All | -1.58 | -1.58 | -1.48 | | -1.54 | -1.12 | -1.05 |
| CAMKII | CL12768.Contig1_All | -1.40 | -1.27 | -0.77 | | -0.24 | -0.74 | -1.11 |
| CAMKII | CL12768.Contig2_All | -1.73 | -1.47 | -1.69 | | -0.18 | -1.89 | -2.90 |
| CAMKII | CL12768.Contig3_All | -2.01 | -2.16 | -2.20 | | -0.40 | -1.39 | -2.38 |
| PLC | CL2281.Contig1_All | -1.14 | -0.82 | -1.53 | | -0.49 | -1.13 | -1.74 |
| Raf | CL3009.Contig1_All | -1.06 | -0.45 | -0.79 | | -1.14 | -1.27 | -1.68 |
| PKA | CL32.Contig8_All | -1.16 | -0.65 | -1.41 | | -0.29 | -1.31 | -2.30 |
| CAMKII | CL5029.Contig1_All | -1.33 | -1.30 | -1.70 | | 0.11 | -1.30 | -1.78 |
| CBP | CL5613.Contig1_All | -2.26 | -2.48 | -2.66 | | -1.23 | -1.99 | -2.98 |
| CBP | CL5613.Contig2_All | -1.81 | -1.81 | -2.45 | | -1.51 | -1.84 | -2.98 |
| AC1/8 | CL6305.Contig1_All | -7.51 | -2.51 | -2.18 | | 0.05 | -1.37 | -3.35 |
| AC1/8 | CL6305.Contig2_All | -2.49 | -1.87 | -3.31 | | -2.93 | -0.40 | -3.66 |
| Raf | CL6905.Contig2_All | -1.87 | -2.52 | -2.53 | | -1.64 | -1.71 | -2.73 |
| CaM | CL7465.Contig1_All | -1.51 | 1.13 | 3.44 | | 0.94 | 0.73 | 1.32 |
| NMDAR | CL8380.Contig1_All | -3.27 | -2.08 | -0.95 | | -1.70 | -5.99 | -2.61 |
| CBP | Unigene11287_All | -1.66 | -1.72 | -1.44 | | -0.91 | -1.06 | -1.42 |
| EAMK1/2 | Unigene12163_All | -1.48 | -0.86 | -1.60 | | -0.47 | -1.43 | -2.05 |
| CBP | Unigene14163_All | -2.09 | -2.08 | -4.08 | | -0.91 | -2.45 | -4.97 |
| CBP | Unigene14323_All | -2.51 | -2.07 | -3.34 | | -0.90 | -1.57 | -2.34 |
| PKA | Unigene14542_All | -1.47 | -0.76 | -1.76 | | -0.13 | -1.40 | -2.30 |
| CBP | Unigene3340_All | -1.10 | -0.59 | -1.38 | | -0.45 | -1.52 | -1.47 |
| PKC | Unigene40306_All | -2.98 | -1.19 | -2.48 | | -0.60 | -1.64 | -2.37 |
| RSK | Unigene5967_All | -1.69 | -1.16 | -1.92 | | -0.49 | -1.81 | -2.26 |
| CAMKIV | Unigene5996_All | -2.11 | -1.44 | -2.25 | | -0.34 | -2.08 | -3.13 |
| RSK | Unigene6175_All | -1.88 | -1.22 | -2.07 | | -0.91 | -1.86 | -2.29 |
| AC1/8 | Unigene67597_All | -1.81 | -1.27 | -1.53 | | -1.08 | -1.74 | -2.18 |
| PP1 | Unigene70495_All | 1.52 | -0.30 | 0.89 | | 0.06 | 0.94 | 0.39 |
| Raf | Unigene78202_All | 1.09 | 0.60 | 0.27 | | 0.26 | 0.67 | -0.36 |
| Raf | Unigene7991_All | -1.29 | -1.44 | -1.39 | | -1.31 | -0.92 | -0.55 |
| CBP | CL2219.Contig1_All | -0.97 | -1.76 | -1.75 | | -1.67 | -1.25 | -1.97 |
| Raf | CL2444.Contig1_All | -0.32 | -1.23 | -0.97 | | -0.66 | -0.23 | -0.47 |
| CaM | CL7465.Contig2_All | 0.92 | 1.79 | -1.26 | | -2.44 | -2.74 | -1.11 |
| NMDAR | CL8380.Contig2_All | -1.59 | -2.45 | -1.95 | | -2.58 | -2.51 | -4.06 |
| Raf | Unigene11367_All | -0.56 | -3.38 | 0.39 | | -1.35 | -1.75 | -4.69 |
| mGluR | Unigene12739_All | -0.80 | -1.07 | -0.69 | | -0.59 | -0.69 | -1.09 |
| CaM | Unigene1278_All | -0.17 | 1.90 | 3.22 | | -0.22 | 1.91 | 3.55 |
| RSK | Unigene14685_All | -1.27 | -5.08 | -2.13 | | -1.81 | -1.64 | -1.78 |
| Raf | Unigene29393_All | 0.08 | -2.12 | -0.34 | | -0.69 | -0.62 | -0.32 |
| PP1 | Unigene62160_All | -1.41 | -1.78 | -1.81 | | -1.01 | -2.27 | -3.50 |
| PKC | Unigene72446_All | -0.97 | -1.17 | -1.91 | | -0.39 | -1.44 | -1.91 |
| CaM | Unigene9663_All | -0.70 | 1.03 | 2.38 | | 0.09 | 2.96 | 2.18 |
| CaM | Unigene96_All | -0.55 | -1.11 | -0.63 | | -0.50 | -0.42 | -0.89 |
| PLC | CL2281.Contig2_All | -0.92 | -1.00 | -1.49 | | -0.40 | -1.28 | -2.48 |
| Rap1 | CL3865.Contig2_All | -0.99 | -0.33 | -1.27 | | 0.03 | -0.78 | -1.75 |
| Raf | CL4034.Contig4_All | -1.27 | -0.42 | -2.71 | | -1.98 | -3.14 | -2.59 |
| Raf | CL6948.Contig1_All | -2.18 | -0.21 | -2.36 | | -1.31 | -1.86 | -0.88 |
| Raf | CL8054.Contig1_All | -0.53 | 0.51 | -3.44 | | -1.12 | -0.27 | 0.35 |
| Ras | Unigene11311_All | -0.86 | -0.46 | -1.08 | | -0.54 | -0.88 | -0.98 |
| PKC | Unigene11828_All | -0.94 | -0.91 | -1.36 | | -0.75 | -1.71 | -1.64 |
| CBP | Unigene70761_All | -0.70 | -0.90 | -3.98 | | 0.30 | -1.05 | -3.01 |
| PP1 | Unigene79417_All | -0.79 | -0.24 | -1.48 | | -0.68 | -1.63 | -2.14 |
| PP1 | CL6805.Contig3_All | -0.94 | -0.84 | -0.69 | | -1.51 | -0.60 | 0.11 |
| CBP | Unigene32605_All | -0.04 | -0.22 | -0.54 | | -1.11 | 0.04 | -0.20 |
| AC1/8 | Unigene9645_All | -0.51 | -0.79 | -0.81 | | -1.07 | -0.96 | -0.73 |
| PKC | CL11461.Contig1_All | -0.70 | -1.98 | -1.94 | | -1.20 | -2.86 | -2.01 |
| Raf | CL4304.Contig1_All | -0.21 | 0.07 | -0.67 | | -1.12 | -1.25 | -0.40 |
| CaM | CL6746.Contig1_All | -0.47 | 0.09 | -0.51 | | -0.02 | -1.70 | -0.76 |
| PKC | CL11461.Contig3_All | -2.27 | -2.17 | -2.46 | | -0.12 | -1.86 | -3.43 |
| PLC | CL11624.Contig1_All | -0.61 | -0.92 | -0.27 | | -0.82 | -0.53 | -1.99 |
| Rap1 | CL3865.Contig1_All | -0.40 | -0.19 | -0.46 | | 0.24 | -0.32 | -1.02 |
| CaM | CL7675.Contig2_All | 0.09 | -0.40 | -0.39 | | -0.18 | -1.25 | -2.32 |
| PP1 | Unigene4739_All | -0.60 | -0.71 | -0.71 | | -0.31 | -0.74 | -1.25 |
| AC1/8 | Unigene58654_All | -2.46 | -1.33 | -2.63 | | -2.58 | -2.38 | -3.69 |
| **Neurotrophin signaling pathway** | |  |  |  | |  |  |  |
| CaMK | CL12768.Contig1_All | -1.40 | -1.27 | -0.77 | | -0.24 | -0.74 | -1.11 |
| CaMK | CL12768.Contig2_All | -1.73 | -1.47 | -1.69 | | -0.18 | -1.89 | -2.90 |
| CaMK | CL12768.Contig3_All | -2.01 | -2.16 | -2.20 | | -0.40 | -1.39 | -2.38 |
| B-Raf | CL3009.Contig1_All | -1.06 | -0.45 | -0.79 | | -1.14 | -1.27 | -1.68 |
| JNK | CL3431.Contig1_All | -1.25 | -0.77 | -1.17 | | -0.61 | -0.80 | -1.71 |
| JNK | CL3431.Contig3_All | -1.53 | -0.92 | -1.37 | | -0.17 | -1.39 | -2.11 |
| ARMS | CL4053.Contig1_All | -2.04 | -1.03 | -0.80 | | -1.86 | -1.35 | -1.43 |
| ARMS | CL4053.Contig3_All | -1.22 | -1.49 | -1.09 | | -0.96 | -0.90 | -1.21 |
| CaMK | CL5029.Contig1_All | -1.33 | -1.30 | -1.70 | | 0.11 | -1.30 | -1.78 |
| B-Raf | CL6905.Contig2_All | -1.87 | -2.52 | -2.53 | | -1.64 | -1.71 | -2.73 |
| CaM | CL7465.Contig1_All | -1.51 | 1.13 | 3.44 | | 0.94 | 0.73 | 1.32 |
| MEKK1 | CL7517.Contig3_All | -1.20 | -0.85 | -1.86 | | -0.99 | -1.10 | -3.70 |
| Cdc42 | Unigene10468_All | -1.27 | -0.82 | -1.58 | | -0.91 | -1.72 | -1.78 |
| Shc | Unigene12016_All | -1.25 | -0.72 | -1.19 | | -0.46 | -1.41 | -1.51 |
| Rac1 | Unigene12065_All | -2.04 | -1.53 | -2.10 | | 0.09 | -1.69 | -2.79 |
| Erk1/2 | Unigene12163_All | -1.48 | -0.86 | -1.60 | | -0.47 | -1.43 | -2.05 |
| PLCy | Unigene14050_All | -2.02 | -1.80 | -2.30 | | -0.70 | -1.65 | -1.69 |
| TRAF6 | Unigene1475_All | -1.72 | -0.62 | -2.04 | | -0.47 | -1.59 | -1.34 |
| Akt | Unigene26509_All | -1.41 | -1.20 | -1.80 | | -0.48 | -1.09 | -1.34 |
| Akt | Unigene4933_All | -2.41 | -1.58 | -2.02 | | -0.53 | -1.42 | -2.94 |
| P90rsk | Unigene5967_All | -1.69 | -1.16 | -1.92 | | -0.49 | -1.81 | -2.26 |
| CaMK | Unigene5996_All | -2.11 | -1.44 | -2.25 | | -0.34 | -2.08 | -3.13 |
| P90rsk | Unigene6175_All | -1.88 | -1.22 | -2.07 | | -0.91 | -1.86 | -2.29 |
| PI3K | Unigene6308_All | -1.38 | -0.75 | -0.96 | | 0.01 | -0.56 | -0.83 |
| SOS | Unigene6353_All | -1.60 | -1.47 | -2.39 | | -0.75 | -1.44 | -2.19 |
| Crk | Unigene6462_All | -1.49 | -1.46 | -1.52 | | -0.67 | -1.12 | -1.51 |
| Akt | Unigene69837_All | -2.54 | -1.45 | -2.80 | | -0.97 | -1.44 | -1.61 |
| B-Raf | Unigene78202_All | 1.09 | 0.60 | 0.27 | | 0.26 | 0.67 | -0.36 |
| B-Raf | Unigene7991_All | -1.29 | -1.44 | -1.39 | | -1.31 | -0.92 | -0.55 |
| GRB2 | Unigene8881_All | -1.14 | -0.52 | -1.43 | | -0.26 | -1.20 | -1.55 |
| PI3K | Unigene9455_All | -1.78 | -1.67 | -2.48 | | -0.57 | -1.60 | -2.04 |
| MSK1 | Unigene9508_All | -1.68 | -1.44 | -1.85 | | -0.53 | -1.38 | -2.19 |
| B-Raf | CL2444.Contig1_All | -0.32 | -1.23 | -0.97 | | -0.66 | -0.23 | -0.47 |
| ARMS | CL2855.Contig2_All | -6.53 | -8.34 | -6.59 | | 5.43 | 0.00 | -2.69 |
| IRS | CL3514.Contig2_All | -1.35 | -1.51 | -1.13 | | -0.93 | -0.62 | -0.54 |
| IRAK | CL3983.Contig2_All | -0.91 | -1.25 | -1.66 | | -0.79 | -0.85 | -1.23 |
| CaM | CL7465.Contig2_All | 0.92 | 1.79 | -1.26 | | -2.44 | -2.74 | -1.11 |
| SOS | CL8920.Contig1_All | -2.86 | -3.72 | -2.13 | | 0.47 | -2.37 | -2.11 |
| B-Raf | Unigene11367_All | -0.56 | -3.38 | 0.39 | | -1.35 | -1.75 | -4.69 |
| CaM | Unigene1278_All | -0.17 | 1.90 | 3.22 | | -0.22 | 1.91 | 3.55 |
| p90rsk | Unigene14685_All | -1.27 | -5.08 | -2.13 | | -1.81 | -1.64 | -1.78 |
| GRB2 | Unigene2081_All | -1.22 | -1.92 | -2.19 | | -1.20 | -1.41 | -2.13 |
| B-Raf | Unigene29393_All | 0.08 | -2.12 | -0.34 | | -0.69 | -0.62 | -0.32 |
| cAb1 | Unigene5214_All | -0.85 | -1.25 | -0.97 | | -0.92 | -0.60 | -0.97 |
| CaM | Unigene9663_All | -0.70 | 1.03 | 2.38 | | 0.09 | 2.96 | 2.18 |
| CaM | Unigene96_All | -0.55 | -1.11 | -0.63 | | -0.50 | -0.42 | -0.89 |
| JNK | CL3431.Contig2_All | -0.94 | -0.77 | -1.06 | | -0.27 | -1.10 | -1.62 |
| Rap1 | CL3865.Contig2_All | -0.99 | -0.33 | -1.27 | | 0.03 | -0.78 | -1.75 |
| B-Raf | CL4034.Contig4_All | -1.27 | -0.42 | -2.71 | | -1.98 | -3.14 | -2.59 |
| GSK3β | CL4139.Contig2_All | -0.56 | -0.93 | -1.10 | | -0.95 | -0.51 | -0.96 |
| SH-PTP2 | CL4485.Contig3_All | -0.70 | -0.28 | -1.24 | | -0.08 | 0.24 | -1.94 |
| PI3K | CL4556.Contig2_All | -0.99 | -0.70 | -1.73 | | -0.19 | -1.20 | -2.30 |
| B-Raf | CL6948.Contig1_All | -2.18 | -0.21 | -2.36 | | -1.31 | -1.86 | -0.88 |
| B-Raf | CL8054.Contig1_All | -0.53 | 0.51 | -3.44 | | -1.12 | -0.27 | 0.35 |
| PI3K | Unigene11052_All | -2.37 | -0.66 | -7.60 | | -0.57 | -2.05 | -2.11 |
| Ras | Unigene11311_All | -0.86 | -0.46 | -1.08 | | -0.54 | -0.88 | -0.98 |
| Akt | Unigene68579_All | -1.14 | -0.66 | -1.74 | | -0.77 | -1.51 | -2.43 |
| ARMS | CL4053.Contig2_All | -0.48 | -0.75 | -0.87 | | -1.04 | -0.20 | -0.30 |
| 14-3-3ε | CL1070.Contig2_All | -0.94 | -0.46 | -0.61 | | -0.53 | -1.11 | -1.71 |
| B-Raf | CL4304.Contig1_All | -0.21 | 0.07 | -0.67 | | -1.12 | -1.25 | -0.40 |
| PI3K | CL4556.Contig1_All | -0.69 | -0.41 | -0.83 | | -0.58 | -1.35 | -1.98 |
| CaM | CL6746.Contig1_All | -0.47 | 0.09 | -0.51 | | -0.02 | -1.70 | -0.76 |
| MAHKAH2 | CL9635.Contig1_All | -0.96 | -0.22 | -0.74 | | -0.54 | -1.23 | -1.20 |
| RhoA | CL12400.Contig2_All | -0.61 | -0.06 | -0.68 | | 0.02 | -0.80 | -1.05 |
| Rap1 | CL3865.Contig1_All | -0.40 | -0.19 | -0.46 | | 0.24 | -0.32 | -1.02 |
| PSEN | CL3965.Contig1_All | -0.55 | -0.33 | -0.60 | | -0.26 | -0.92 | -1.06 |
| 14-3-3ε | CL4762.Contig1_All | -0.52 | -0.02 | -0.47 | | 0.07 | -0.84 | -1.27 |
| Rac1 | CL5575.Contig6_All | 0.13 | 0.26 | -0.04 | | 0.57 | -1.20 | -1.20 |
| CaM | CL7675.Contig2_All | 0.09 | -0.40 | -0.39 | | -0.18 | -1.25 | -2.32 |
| IKKβ | Unigene1231_All | -0.99 | -0.49 | -0.79 | | -0.15 | -0.99 | -1.50 |
| RhoA | Unigene13585_All | -0.78 | -0.33 | -0.66 | | -0.06 | -0.83 | -1.20 |
| C3G | Unigene1395_All | -2.44 | -1.30 | -0.94 | | -0.86 | -3.86 | -2.49 |
| GSK3β | Unigene14019_All | -1.11 | -0.46 | -0.71 | | -0.99 | -1.14 | -1.53 |
| FKHRL1 | Unigene79098_All | -1.21 | -0.40 | -0.44 | | -1.01 | -1.75 | -1.93 |
| **Retrograde endocannabinoid signaling pathway** | |  |  |  | |  |  |  |
| AC | CL12536.Contig1_All | -1.52 | -1.25 | -2.49 | | -1.78 | -1.27 | -1.11 |
| AC | CL12536.Contig2_All | -1.58 | -1.58 | -1.48 | | -1.54 | -1.12 | -1.05 |
| GABAA | CL1881.Contig2_All | -1.02 | -0.22 | -0.44 | | -0.86 | -0.91 | -1.13 |
| PLCβ | CL2281.Contig1_All | -1.14 | -0.82 | -1.53 | | -0.49 | -1.13 | -1.74 |
| VGCC | CL2283.Contig1_All | -2.48 | -2.77 | -3.26 | | -2.93 | -2.57 | -2.51 |
| PKA | CL32.Contig8_All | -2.48 | -0.65 | -1.41 | | -0.29 | -1.31 | -2.30 |
| MAPK | CL3431.Contig1_All | -1.25 | -0.77 | -1.17 | | -0.61 | -0.80 | -1.71 |
| MAPK | CL3431.Contig3_All | -1.53 | -0.92 | -1.37 | | -0.17 | -1.39 | -2.11 |
| AC | CL5341.Contig1_All | -1.38 | -1.33 | -2.23 | | -1.28 | -1.54 | -2.65 |
| GABAA | CL548.Contig1_All | -5.09 | -3.00 | -5.81 | | -2.12 | -7.69 | -11.29 |
| AC | CL6305.Contig1_All | -7.51 | -2.51 | -2.18 | | 0.05 | -1.37 | -3.35 |
| AC | CL6305.Contig2_All | -2.49 | -1.87 | -3.31 | | -2.93 | -0.40 | -3.66 |
| AMPAR | CL66.Contig4_All | -2.48 | -0.28 | -1.27 | | -0.48 | -0.64 | -2.43 |
| AMPAR | CL66.Contig6_All | -1.59 | -0.59 | -1.78 | | -0.37 | -1.86 | -3.00 |
| GABAA | CL6923.Contig2_All | -1.66 | -1.74 | -1.57 | | -0.25 | -0.52 | -1.36 |
| Gi/o | CL7798.Contig2_All | -1.09 | -1.63 | -0.62 | | -1.26 | -0.92 | -0.96 |
| Gi/o | Unigene1049_All | -1.10 | -0.39 | -0.98 | | -0.48 | -1.39 | -1.56 |
| MAPK | Unigene12163_All | -1.48 | -0.86 | -1.60 | | -0.47 | -1.43 | -2.05 |
| Gi/o | Unigene13908_All | -2.07 | -1.47 | -2.32 | | -0.54 | -2.02 | -2.61 |
| PKA | Unigene14542_All | -1.47 | -0.76 | -1.76 | | -0.13 | -1.40 | -2.30 |
| PKC | Unigene40306_All | -2.98 | -1.19 | -2.48 | | -0.60 | -1.64 | -2.37 |
| VGAT | Unigene4955_All | -2.36 | -3.65 | -3.73 | | -1.94 | -2.31 | -2.69 |
| RIM1α | Unigene58140_All | -3.37 | -2.84 | -3.34 | | -1.88 | -3.22 | -4.75 |
| AC | Unigene64589_All | -2.59 | -1.03 | -2.09 | | -0.41 | -1.41 | -2.73 |
| AC | Unigene65078_All | -1.19 | -1.70 | -2.06 | | -1.05 | -1.05 | -3.16 |
| AC | Unigene67597_All | -1.81 | -1.27 | -1.53 | | -1.08 | -1.74 | -2.18 |
| VGAT | Unigene76468_All | -4.50 | -2.90 | -3.39 | | -0.02 | -1.54 | -4.43 |
| AC | CL6134.Contig2_All | -2.50 | -3.02 | -2.16 | | -1.12 | -4.54 | -3.28 |
| Gi/o | CL7798.Contig1_All | -0.73 | -1.73 | -1.04 | | -0.82 | -1.10 | -1.07 |
| Vglut | CL9844.Contig2_All | 0.21 | -1.19 | 0.10 | | -0.08 | 1.21 | 0.92 |
| VGCC | Unigene12299_All | -0.87 | -1.54 | 0.79 | | -0.91 | 0.72 | 0.50 |
| FAAH | Unigene12526_All | -0.90 | -1.27 | -0.21 | | 0.19 | -0.50 | -1.27 |
| mGluRs | Unigene12739_All | -0.80 | -1.07 | -0.69 | | -0.59 | -0.69 | -1.09 |
| PKC | Unigene72446_All | -0.97 | -1.17 | -1.91 | | -0.39 | -1.44 | -1.91 |
| PLCβ | CL2281.Contig2_All | -0.92 | -1.00 | -1.49 | | -0.40 | -1.28 | -2.48 |
| MAPK | CL3431.Contig2_All | -0.94 | -0.77 | -1.06 | | -0.27 | -1.10 | -1.62 |
| VGCC | CL4555.Contig2_All | -8.68 | -1.34 | -9.66 | | -6.70 | -7.73 | -9.61 |
| VGAT | CL6277.Contig1_All | -3.21 | -0.69 | -2.69 | | -1.23 | -3.05 | -2.77 |
| AMPAR | CL66.Contig5_All | -1.47 | -0.70 | -2.09 | | -0.18 | -0.96 | -2.43 |
| PKC | Unigene11828_All | -0.94 | -0.91 | -1.36 | | -0.75 | -1.71 | -1.64 |
| AMPAR | Unigene24013_All | -0.89 | -0.35 | -1.01 | | -0.30 | -1.12 | -1.67 |
| GABAA | Unigene57709_All | -1.55 | -1.71 | -2.90 | | -1.38 | -1.17 | -10.37 |
| AMPAR | Unigene71686_All | -0.72 | -0.63 | -1.06 | | 0.11 | -0.79 | -2.71 |
| AMPAR | Unigene72065_All | -0.36 | -0.67 | -1.50 | | -0.16 | -1.11 | -3.63 |
| GAGL | Unigene74835_All | -1.27 | -1.20 | -3.88 | | -1.12 | -2.38 | -4.66 |
| GABAA | Unigene83870_All | -1.04 | -2.40 | -10.59 | | -0.93 | -2.05 | -3.43 |
| VGAT | Unigene27251_All | -0.19 | -0.41 | -0.92 | | -1.63 | -0.19 | 0.01 |
| VGAT | Unigene3045_All | -0.80 | -0.18 | -0.62 | | -1.19 | 0.26 | 0.25 |
| GABAA | Unigene3712_All | 0.53 | -0.78 | 0.63 | | -1.27 | -2.07 | -2.01 |
| AC | Unigene9645_All | -0.51 | -0.79 | -0.81 | | -1.07 | -0.96 | -0.73 |
| PKC | CL11461.Contig1_All | -0.70 | -1.98 | -1.94 | | -1.20 | -2.86 | -2.01 |
| Gi/o | CL5187.Contig2_All | -0.88 | 0.42 | 0.41 | | -0.14 | -1.00 | -0.51 |
| VGCC | Unigene12233_All | 0.36 | 0.63 | 0.62 | | 0.39 | 1.39 | 1.33 |
| VGAT | Unigene26255_All | -1.53 | -0.99 | -1.40 | | -0.97 | -2.49 | -1.72 |
| PKC | CL11461.Contig3_All | -2.27 | -2.17 | -2.46 | | -0.12 | -1.86 | -3.43 |
| PLCβ | CL11624.Contig1_All | -0.61 | -0.92 | -0.27 | | -0.82 | -0.53 | -1.99 |
| AMPAR | CL66.Contig2_All | -0.39 | -0.30 | -0.98 | | -0.32 | -0.73 | -1.48 |
| AMPAR | CL66.Contig3_All | -0.33 | -0.48 | -0.62 | | -0.26 | -0.63 | -1.12 |
| VGAT | CL9007.Contig1_All | -0.92 | -0.15 | -0.99 | | -0.10 | -0.54 | -1.06 |
| FAAH | Unigene25918_All | 6.01 | 0.46 | 0.54 | | 0.16 | -0.05 | -4.01 |
| AMPAR | Unigene31894_All | -1.78 | -0.42 | -1.72 | | -1.88 | -1.75 | -2.69 |
| AC | Unigene32128_All | -1.25 | -1.20 | -0.83 | | -1.31 | -1.64 | -2.38 |
| GABAA | Unigene40418_All | -3.95 | -1.61 | -3.21 | | -1.93 | -2.37 | -4.43 |
| AC | Unigene58654_All | -2.46 | -1.33 | -2.63 | | -2.58 | -2.38 | -3.69 |
| RIM1α | Unigene62797_All | -0.70 | -1.66 | -1.55 | | 0.30 | -8.92 | -11.22 |
| AMPAR | Unigene71685_All | -0.80 | -0.08 | -1.10 | | 0.24 | -1.22 | -2.69 |
| Gi/o | Unigene99_All | -0.43 | -0.21 | -0.84 | | -0.24 | -0.46 | -1.23 |

Table S7. qRT-PCR primers

| **Pathway**  **/member** | **Unigene** | **Sense Primer** | **Anti-sense Primer** |
| --- | --- | --- | --- |
| **Serotonergic synapse pathway** | | | |
| MAO | Unigene11212_All | TCCTGCTGCGTTACATAT | GCTCTTCTCACAATATCTTCAT |
| SERT | Unigene63181_All | CATCCGCTACTACCTCAC | GTTGTTGTTGAACTTGTTGTAG |
| 5-HT1AF | Unigene76812_All | ATCCACCTGATGCTTCAC | ACGGCTACAAATGTTCCA |
| VGCC | CL4555.Contig2_All | GTGGTGCGTAACTTGATG | GGTGGTCATAGTCCTTCC |
| **Synaptic vesicle cycle** | | | |
| V-ATPase | CL3817.Contig4_All | CTGAGTGTGATTGATGAGTTG | TGATATTGAAGATGTGCTTATGG |
| RIM | Unigene62797_All | CCCAATGACTCCGAAACT | GCGACAAGGTACTGGAAT |
| **Cholinergic synapse** | | | |
| AChE | Unigene2440_All | CAGCGTCTATAATACCTCCTA | GCCATATTCAGTAGCATCG |
| AChE | Unigene9281_All | ATCTGAAGAAGACCGTGTA | GGATTCGTTGTTGTAGGATT |
| PI3K | Unigene11052_All | ATGGTTGGACGACATTCA | ACACTTCTTGCCTCTTCTT |
| **Dopaminergic synapse** | | | |
| MAO | Unigene11212_All | GGACACTAATTGGTTCATCAT | AGATACACATTGGCTATTGC |
| Cav2.1/2.2 | CL4555.Contig2_All | GTGGTGCGTAACTTGATG | GGTGGTCATAGTCCTTCC |
| NMDAr | CL8380.Contig1_All | AATCACATTACTTCAACAGAGT | CACACCAGTTAGCACAAG |
| **GABAergic synapse** | | | |
| GLS | Unigene5837_All | ATTGCTCCACTCACACTT | CAGAACAGAGGATACTTGATTC |
| GAD | Unigene1151_All | TGCTTGTCTCCTGTATCATAT | TGCTCAACATTCCTGCTA |
| AC | CL6305.Contig1_All | GTGGTGGAGAATGTGAAC | TTGAAGATGTGCCTGACT |
| GABAA | CL548.Contig1_All | TGTCAGCCATAGAATCCAT | GCAACCAGCAGTCAATAG |
| **Glutamatergic synapse** | | | |
| mGluR2/3 | Unigene78451_All | GAGGCTGAGTGTATTCCA | GGTGTCGTATATCAATGTGTAT |
| **Long-term depression** | | | |
| G | Unigene13012_All | GAGTCGCTGATGATGGAG | AGTCAACTGGAGTGATGTC |
| G | Unigene41131_All | TGGCATTGTCGTCTCATA | TGTGCTGATGGAATAATGTTAA |
| **Long-term potentiation** | | | |
| CaM-1 | CL7465.Contig1_All | CCTTCTTCAGCAGTTGTATC | CATTTCACGGCGTCTTTG |
| CaM-2 | Unigene9663_All | GATTCAGAGCAGGTCCAA | GTTCTCGTCCTTGTCCTC |
| CaM-3 | CL7465.Contig2_All | TGCCACCAGAACAGATAC | ATCACGAACTCCTCAAACT |
| **Neurotrophin signaling pathway** | | | |
| ARMS | CL2855.Contig2_All | ATGGCTCGTCTTAATCTCA | GGCACTGGCTACTTCATA |
| **Retrograde endocannabinoid signaling** | | | |
| FAAH | Unigene25918_All | TACCATTGACTACTCTTATTCCA | TATTGCTTCCTCCTATGTATATCT |
| GABAA | Unigene83870_All | TGCCGTTGTAGAAGTAGG | CCATATCCGAGGTAGACAT |
| **Others** | | | |
| mGluRs | Unigene12739_All | CCGAGCAATCCTATTCCT | GTATCCACCTCCACCATT |
| Gi/o | Unigene99_All | ACCTTCGGCTGTATTGTC | GTGTCAGTGGCAGTAGTAA |

**Table S8. Immune process-related DEGs (log2ratio)**

| Family/members | 4 h pi | 12 h pi | 24 h pi | 36 h pi | 48 h pi | 72 h pi |
| --- | --- | --- | --- | --- | --- | --- |
| **Recognition** |  |  |  |  |  |  |
| **c-type lectin** |  |  |  |  |  |  |
| Unigene4485_All | 1.08 |  |  |  |  |  |
| Unigene580_All | -6.53 | 1.70 |  | 2.05 | 2.53 | 4.59 |
| CL5193.Contig1_All |  | 1.59 |  |  |  |  |
| Unigene79501_All | -1.74 | -1.46 | -2.50 |  | -1.51 | -3.69 |
| Unigene1489_All |  |  | 1.48 |  | 1.54 | 1.56 |
| Unigene57398_All | 1.37 | -1.49 |  | 3.34 |  |  |
| CL5301.Contig3_All |  |  |  |  | -1.37 | -3.63 |
| Unigene12426_All |  |  |  |  |  | -1.86 |
| Unigene726_All |  |  |  |  | -1.20 | -1.26 |
| CL8739.Contig1_All |  |  |  |  |  | -1.02 |
| up/down | 2/2 | 2/2 | 1/1 | 2/0 | 2/3 | 2/5 |
| total | 4 | 4 | 2 | 2 | 5 | 7 |
|  |  |  |  |  |  |  |
| **galectin** |  |  |  |  |  |  |
| CL9233.Contig1_All | -1.51 | -1.58 | -1.50 | -1.40 | -1.10 | -1.56 |
| Unigene5995_All | -1.29 |  | -1.47 |  | -1.43 | -1.68 |
| Unigene12505_All |  | -1.26 |  |  |  |  |
| up/down | 0/2 | 0/2 | 0/2 | 0/1 | 0/2 | 0/2 |
| total | 2 | 2 | 2 | 1 | 2 | 2 |
|  |  |  |  |  |  |  |
| **gnbp** |  |  |  |  |  |  |
| CL13615.Contig2_All |  |  |  |  |  | 1.00 |
| CL13615.Contig1_All |  |  |  |  |  | -1.60 |
| up/down |  |  |  |  |  | 1/1 |
| total |  |  |  |  |  | 2 |
|  |  |  |  |  |  |  |
| **pgrp** |  |  |  |  |  |  |
| Unigene6863_All | -4.04 |  |  | -1.05 | 1.90 | 1.91 |
| Unigene13556_All | -2.63 |  |  |  | 2.59 | 2.64 |
| Unigene3199_All | -2.51 | -3.30 | -3.05 | -1.61 | -2.74 | -3.08 |
| CL2777.Contig1_All | -2.21 |  |  |  |  |  |
| CL2777.Contig3_All | -2.00 |  |  |  |  |  |
| Unigene27669_All | -1.77 | -1.29 | -1.00 |  | -2.28 | -2.34 |
| CL10428.Contig3_All | -1.09 |  |  |  |  |  |
| CL8908.Contig2_All | -2.37 | -4.08 | -7.47 | -7.42 | -2.05 | -8.68 |
| CL1005.Contig6_All |  |  |  |  |  |  |
| CL1005.Contig5_All |  |  |  |  |  |  |
| Unigene3005_All | -1.37 | -1.54 | -2.19 |  | -1.96 | -2.38 |
| Unigene9349_All |  |  | 1.05 |  |  |  |
| CL5522.Contig1_All |  |  |  | -2.58 | 3.17 | 4.36 |
| CL5522.Contig2_All |  |  |  | -1.71 | 1.90 | 3.72 |
| CL6169.Contig1_All |  |  |  |  | 1.11 |  |
| Unigene3200_All | -1.97 |  | -1.30 |  | -1.78 | -1.86 |
| Unigene12840_All |  |  |  |  |  | -1.45 |
| CL7813.Contig4_All |  |  |  |  |  | -1.26 |
| CL7813.Contig2_All |  |  |  |  |  | -1.19 |
| up/down | 0/10 | 0/4 | 1/5 | 0/5 | 5/5 | 4/8 |
| total | 10 | 4 | 6 | 5 | 10 | 12 |
|  |  |  |  |  |  |  |
| **scavenger receptor class a** | | | | | | |
| Unigene41131_All | 10.38 | 2.45 | -10.46 |  | -4.51 |  |
| Unigene1014_All | 4.35 | 1.38 | 1.54 |  |  | 2.21 |
| Unigene13012_All | 3.60 | 9.96 | 1.42 |  | -3.43 | -1.11 |
| CL7437.Contig2_All | -3.29 | 4.69 |  | -2.29 | -4.81 | -2.69 |
| Unigene10890_All | -2.58 | -2.94 | -3.07 | -1.86 | -1.05 | -3.28 |
| Unigene1722_All | -2.37 | -2.03 | -1.98 |  | -1.40 | -3.39 |
| CL4043.Contig11_All | -1.82 |  | -2.20 | -1.66 | -2.78 | -2.15 |
| CL4043.Contig6_All | -1.55 |  | -1.85 | -1.23 | -2.40 | -2.89 |
| CL4043.Contig3_All | -1.51 |  | -1.79 | -1.63 | -2.75 | -2.18 |
| CL4043.Contig8_All | -1.36 |  | -2.04 | -1.10 | -2.08 | -1.58 |
| CL1500.Contig1_All | -1.15 |  | -1.05 | -1.09 |  | -1.81 |
| CL4043.Contig5_All | -1.13 |  | -1.00 | -1.28 | -1.23 | -1.47 |
| Unigene8293_All | -1.09 | -1.51 |  |  |  | -1.19 |
| Unigene8442_All | 1.81 | 5.01 | 4.04 |  | -1.65 | -1.30 |
| Unigene6240_All | 1.25 | 3.32 |  |  | -3.86 |  |
| Unigene57837_All |  | -3.98 | -2.16 |  | -1.37 | -2.57 |
| Unigene12449_All |  |  | 2.04 |  | 1.37 |  |
| CL1325.Contig1_All |  | -1.71 | -8.40 | -1.70 | -1.05 | -5.19 |
| CL11334.Contig3_All | -2.04 |  | -4.10 | -1.21 | -2.75 | -5.89 |
| CL4043.Contig10_All | -1.63 |  | -2.20 | -1.80 | -2.55 | -2.19 |
| CL4043.Contig9_All | -1.63 |  | -2.11 | -1.58 | -2.64 | -2.07 |
| CL4043.Contig12_All | -1.55 |  | -2.08 | -1.80 | -2.64 | -2.07 |
| CL4043.Contig2_All | -1.45 |  | -2.04 | -1.70 | -2.98 | -2.18 |
| CL4043.Contig7_All | -1.27 |  | -1.77 | -1.42 | -2.01 | -2.07 |
| Unigene1380_All | -1.07 |  | -1.61 |  |  | -1.48 |
| Unigene5276_All |  |  |  | 1.22 | 2.86 | 1.49 |
| CL8075.Contig1_All |  |  | -1.27 | -9.82 | 1.59 |  |
| CL2879.Contig1_All | -1.63 |  |  |  | 3.06 | 2.95 |
| CL2879.Contig2_All | -1.12 |  |  |  | 1.84 | 1.35 |
| CL2879.Contig4_All |  |  |  |  | 1.12 | 1.89 |
| Unigene6240_All | 1.25 | 3.32 |  |  | -3.86 |  |
| Unigene2065_All |  | 9.11 |  |  | -3.34 |  |
| CL3161.Contig2_All | 1.25 | 2.92 |  |  | -3.02 | -1.11 |
| Unigene510_All |  |  |  |  | -1.40 | -2.92 |
| Unigene7668_All |  |  |  |  |  | 1.68 |
| Unigene7999_All |  |  |  |  |  | 1.51 |
| Unigene80001_All | -2.55 |  | -1.50 | -1.29 | -3.75 | -11.23 |
| Unigene5060_All |  | -2.40 | -2.85 | -1.70 | -1.64 | -3.17 |
| CL4004.Contig3_All |  |  |  |  |  | -1.52 |
| Unigene12148_All |  |  |  |  | -1.11 | -1.26 |
| CL4116.Contig1_All |  |  |  |  |  | -1.15 |
| Unigene1519_All |  |  |  |  |  | -1.06 |
| up/down | 7/20 | 9/6 | 4/21 | 1/18 | 6/27 | 5/31 |
| total | 27 | 15 | 25 | 19 | 33 | 36 |
|  |  |  |  |  |  |  |
| **scavenger receptor class b** | | | | | | |
| CL2610.Contig1_All | -1.01 |  | -1.04 |  |  |  |
| CL7213.Contig2_All |  | 1.12 | -1.02 | -1.90 | -2.66 | -1.64 |
| CL7931.Contig1_All |  |  | 1.28 |  | 1.29 |  |
| CL14526.Contig2_All |  |  | -1.08 |  | -1.18 |  |
| CL7213.Contig1_All |  |  |  | -1.33 | -1.89 | -2.19 |
| Unigene1964_All | -1.78 |  | 1.00 | -1.41 | -2.18 | -1.33 |
| Unigene80409_All |  |  |  |  |  | 1.36 |
| up/down | 0/2 | 1/0 | 2/3 | 0/3 | 1/4 | 1/3 |
| total | 2 | 1 | 5 | 3 | 5 | 4 |
|  |  |  |  |  |  |  |
| **scavenger receptor class c** | | | | | | |
| Unigene75975_All | -1.38 | -1.70 | -1.81 | -1.00 | -1.58 | -2.64 |
| up/down | 0/1 | 0/1 | 0/1 | 0/1 | 0/1 | 0/1 |
| total | 1 | 1 | 1 | 1 | 1 | 1 |
|  |  |  |  |  |  |  |
| **Transduction** |  |  |  |  |  |  |
| **cactus** |  |  |  |  |  |  |
| Unigene2719_All |  |  | -1.05 |  | -1.15 | -1.23 |
| up/down |  |  | 0/1 |  | 0/1 | 0/1 |
| total |  |  | 1 |  | 1 | 1 |
|  |  |  |  |  |  |  |
| **caspar** |  |  |  |  |  |  |
| Unigene75794_All | -1.57 | -1.25 | -1.69 | -1.85 |  |  |
| CL2436.Contig2_All |  | -1.30 |  |  |  |  |
| up/down | 0/1 | 0/2 | 0/1 | 0/1 |  |  |
| total | 1 | 2 | 1 | 1 |  |  |
|  |  |  |  |  |  |  |
| **hopscotch** |  |  |  |  |  |  |
| Unigene14114_All | -2.09 | -1.20 | -1.66 |  |  | -1.35 |
| Unigene14084_All | -1.13 |  | -1.55 |  | -1.48 | -2.26 |
| Unigene3569_All |  |  |  |  |  | -1.03 |
| up/down | 0/2 | 0/1 | 0/2 |  | 0/1 | 0/3 |
| total | 2 | 1 | 2 |  | 1 | 3 |
|  |  |  |  |  |  |  |
| **myd88** |  |  |  |  |  |  |
| Unigene50585_All | -4.74 | -1.78 | -3.41 |  | -7.78 | -10.53 |
| CL9550.Contig2_All | -3.74 | -1.57 | -4.21 | -1.26 | -2.47 | -2.69 |
| Unigene76027_All | -2.52 | -1.67 | -2.37 |  | -2.90 | -3.29 |
| Unigene74097_All | -2.21 | -1.90 | -4.10 | -1.55 | -1.05 | -5.86 |
| Unigene27436_All | -2.04 | -2.42 | -2.72 | -1.34 | -2.05 | -2.67 |
| Unigene3437_All | -1.70 | -1.40 | -2.04 |  | -1.49 | -2.71 |
| Unigene79827_All | -1.67 | -1.18 | -1.95 |  | -1.25 | -2.09 |
| Unigene53339_All | -1.54 |  | -2.50 |  | -1.34 | -2.86 |
| Unigene58450_All | -1.49 | -1.59 | -1.76 | -1.24 | -1.26 | -2.67 |
| CL8146.Contig1_All | -1.23 |  |  |  | -1.64 | -2.12 |
| CL5041.Contig2_All | -1.17 |  | -1.16 |  | -1.42 | -1.95 |
| CL9550.Contig1_All |  | -8.78 | -3.85 | -1.70 | -1.05 | -2.28 |
| CL3419.Contig1_All |  | -1.63 | -2.81 | -1.12 | -2.64 | -4.28 |
| Unigene15419_All |  | -1.54 |  | -1.51 | -2.64 | -6.37 |
| Unigene12623_All |  | -1.38 | -1.55 |  | -1.12 | -1.87 |
| CL14571.Contig2_All |  | -1.34 |  |  |  |  |
| Unigene8276_All |  | -1.21 | -1.11 |  |  | 1.03 |
| Unigene80071_All |  | -1.08 | -1.44 |  | -1.11 | -2.45 |
| CL13369.Contig2_All |  | -1.06 |  |  | -1.30 | -1.67 |
| Unigene81198_All | -1.92 |  | -4.04 | -1.86 | -1.64 | -4.28 |
| CL3354.Contig1_All |  |  | -2.43 | -2.01 | -3.28 | -3.25 |
| CL1168.Contig3_All | -1.43 |  | -1.40 | -1.49 | -3.43 | -4.93 |
| CL4988.Contig2_All |  |  | -1.33 |  | -1.08 | -1.54 |
| Unigene3130_All |  |  |  | 1.74 | -2.11 | -2.93 |
| CL2739.Contig6_All |  |  |  | -1.64 |  |  |
| Unigene64035_All |  |  |  | -1.39 |  | -1.15 |
| CL11053.Contig1_All |  |  |  |  | -1.31 | -2.67 |
| Unigene7994_All |  |  |  |  | -1.12 | -2.06 |
| Unigene28090_All |  |  | -2.18 | -1.82 | -1.05 | -10.51 |
| Unigene80069_All | -2.78 | -1.16 | -2.44 | -1.12 | -2.86 | -4.11 |
| Unigene57212_All |  | -1.48 | -2.21 | -1.40 | -1.79 | -3.36 |
| CL8146.Contig2_All |  |  | -3.85 |  | -1.55 | -2.98 |
| CL4326.Contig4_All | -1.04 |  |  |  |  | -2.92 |
| Unigene12465_All |  |  |  | -1.31 | -1.51 | -1.87 |
| CL938.Contig1_All |  |  |  |  |  | -1.77 |
| CL5041.Contig1_All |  |  |  |  |  | -1.69 |
| CL2739.Contig1_All |  |  |  |  |  | -1.01 |
| up/down | 0/15 | 0/18 | 0/23 | 1/16 | 0/29 | 1/34 |
| total | 15 | 18 | 23 | 17 | 29 | 35 |
|  |  |  |  |  |  |  |
| **pelle** |  |  |  |  |  |  |
| CL1517.Contig2_All | -1.65 | -1.17 | -1.93 |  | -1.64 | -1.99 |
| Unigene4834_All | -1.10 |  | -1.15 |  | -1.03 | -1.45 |
| CL11972.Contig1_All | -1.06 |  |  |  |  |  |
| CL2460.Contig4_All | -1.04 | -1.18 | -1.54 |  |  |  |
| CL1517.Contig1_All |  | -1.09 |  |  |  |  |
| CL13707.Contig2_All | -1.43 |  | -1.60 | -1.36 |  |  |
| CL3071.Contig3_All |  |  |  |  |  |  |
| Unigene15927_All |  |  |  |  |  |  |
| CL11211.Contig1_All | -1.32 | -1.40 | -1.49 | -2.12 | -1.17 | -1.43 |
| Unigene2714_All |  |  |  | -1.05 |  |  |
| CL13707.Contig1_All |  |  |  |  | -1.01 |  |
| up/down | 0/6 | 0/4 | 0/5 | 0/3 | 0/4 | 0/3 |
| total | 6 | 4 | 5 | 3 | 4 | 3 |
|  |  |  |  |  |  |  |
| **relish** |  |  |  |  |  |  |
| CL4028.Contig2_All | -5.51 | 5.84 | 5.41 | 6.29 | 4.80 |  |
| Unigene526_All | -3.41 |  | -2.11 | -1.10 |  | -1.35 |
| CL4187.Contig4_All | -1.20 |  | -1.45 | -1.21 |  |  |
| Unigene4387_All |  |  |  |  |  |  |
| CL7271.Contig1_All | -1.14 |  | -1.57 |  | -1.78 | -1.66 |
| Unigene12949_All | -1.09 |  | -1.38 | -1.56 | -1.59 |  |
| CL6761.Contig6_All | -1.11 | -1.70 | -2.12 | -1.19 | -1.31 | -2.28 |
| CL6761.Contig4_All |  | -1.49 |  | -1.08 |  |  |
| CL13515.Contig2_All |  |  | -1.04 |  |  | -1.45 |
| Unigene3461_All |  |  |  | -1.27 |  |  |
| Unigene2794_All |  |  |  | -1.20 |  |  |
| CL7271.Contig3_All | -1.27 | -1.13 |  | -1.18 | -3.37 |  |
| Unigene80599_All |  |  |  |  | -1.77 | -3.92 |
| CL6761.Contig2_All |  | -1.58 | -2.95 |  |  | -2.92 |
| CL13515.Contig1_All |  |  |  |  |  | -1.19 |
| CL3528.Contig2_All |  |  |  |  |  | -1.13 |
| up/down | 0/7 | 1/4 | 1/7 | 1/8 | 1/5 | 0/8 |
| total | 7 | 5 | 8 | 9 | 6 | 8 |
|  |  |  |  |  |  |  |
| **socs** |  |  |  |  |  |  |
| Unigene8832_All | -1.53 |  |  |  |  |  |
| up/down | 0/1 |  |  |  |  |  |
| total | 1 |  |  |  |  |  |
|  |  |  |  |  |  |  |
| **tak1** |  |  |  |  |  |  |
| Unigene14269_All | -2.42 | -1.19 | -1.75 |  | -1.67 | -1.99 |
| Unigene2769_All | -1.74 | -1.58 | -2.41 |  | -1.81 | -2.68 |
| Unigene7336_All | -1.09 |  |  |  | -1.18 | -1.55 |
| Unigene10497_All |  |  |  |  |  | -1.07 |
| CL4981.Contig1_All |  |  |  |  |  | -1.06 |
| up/down | 0/3 | 0/2 | 0/2 |  | 0/3 | 0/5 |
| total | 3 | 2 | 2 |  | 3 | 5 |
|  |  |  |  |  |  |  |
| **Effectors** |  |  |  |  |  |  |
| **c-type lysozyme** |  |  |  |  |  |  |
| CL6145.Contig2_All |  | 1.40 |  | -1.30 |  |  |
| up/down |  | 1/0 |  | 0/1 |  |  |
| total |  | 1 |  | 1 |  |  |
|  |  |  |  |  |  |  |
| **defensin** |  |  |  |  |  |  |
| Unigene62574_All | -2.04 |  | 2.84 | -2.22 | 1.03 | 5.96 |
| Unigene4090_All |  |  | 1.87 | -1.68 | 1.78 | 5.35 |
| up/down | 0/1 |  | 2/0 | 0/2 | 2/0 | 2/0 |
| total | 1 |  | 2 | 2 | 2 | 2 |
|  |  |  |  |  |  |  |
| **diptericin** |  |  |  |  |  |  |
| CL13977.Contig1_All |  | -1.66 | 2.68 | -1.70 | 4.79 | 4.30 |
| CL13022.Contig2_All | -7.00 | 7.63 | 1.76 | -2.43 | 4.20 | 2.66 |
| up/down | 0/1 | 1/1 | 2/0 | 0/2 | 2/0 | 2/0 |
| total | 1 | 2 | 2 | 2 | 2 | 2 |
|  |  |  |  |  |  |  |
| **nadph oxidase (nox)** |  |  |  |  |  |  |
| Unigene96_All |  | -1.11 |  |  |  |  |
| CL7675.Contig2_All |  |  |  |  | -1.25 | -2.32 |
| up/down |  | 0/1 |  |  | 0/1 | 0/1 |
| total |  | 1 |  |  | 1 | 1 |
|  |  |  |  |  |  |  |
| **prophenoloxidase** |  |  |  |  |  |  |
| Unigene10049_All | 1.68 |  |  |  |  |  |
| Unigene74937_All | -5.88 | -1.16 | 12.04 | 3.44 | -5.61 | -6.30 |
| CL4303.Contig2_All | -1.49 | -1.06 | 1.25 |  | 1.07 |  |
| CL1841.Contig2_All | -1.44 |  |  |  | -1.44 | -2.46 |
| Unigene11938_All | -1.14 |  |  |  | 1.30 |  |
| CL5098.Contig1_All |  | -2.58 | -9.77 |  | 1.67 | 2.46 |
| CL6306.Contig1_All | -6.89 | -1.40 | 8.32 | 7.79 | -8.34 | -8.88 |
| CL7091.Contig4_All |  |  |  | -3.80 | -4.29 | -6.11 |
| CL7091.Contig2_All |  |  |  | -3.46 | -4.14 | -5.37 |
| CL7091.Contig3_All |  |  |  | -2.75 | -4.25 | -4.44 |
| CL11846.Contig1_All |  |  |  | -1.27 | -1.72 | -2.11 |
| Unigene566_All |  |  | 1.69 |  | 2.65 | 2.88 |
| CL7091.Contig1_All |  | 1.05 | -1.16 | -1.81 | -3.70 | -7.02 |
| Unigene9771_All |  |  | -1.21 |  | -3.33 | -2.11 |
| Unigene11359_All |  |  | -1.15 |  | -1.29 | -4.63 |
| up/down | 1/5 | 1/4 | 4/4 | 2/5 | 4/10 | 2/10 |
| total | 6 | 5 | 8 | 7 | 14 | 12 |
|  |  |  |  |  |  |  |
| **Others** |  |  |  |  |  |  |
| **inhibitor of apoptosis** | |  |  |  |  |  |
| CL9445.Contig2_All | -4.36 | -2.08 | -2.21 |  | -3.38 |  |
| CL9816.Contig3_All | -4.04 | -1.66 | -2.16 | -9.12 | -1.75 | -2.92 |
| Unigene8973_All | -3.52 | -3.01 | -4.57 |  | -3.05 | -4.26 |
| CL9816.Contig2_All | -3.24 |  | -1.22 | -1.87 | -2.99 | -1.14 |
| CL871.Contig9_All | -3.22 |  | -1.37 | -2.19 | -1.05 | -2.43 |
| CL4506.Contig2_All | -2.95 | -1.05 | -1.74 |  | -1.21 | -1.49 |
| Unigene26966_All | -2.87 | -2.20 | -4.18 |  | -3.01 | -4.28 |
| Unigene3459_All | -2.72 | -1.74 | -1.99 | -2.29 | -1.64 |  |
| CL871.Contig6_All | -2.68 |  | -1.67 | -1.83 |  | -2.37 |
| Unigene83_All | -2.49 | -2.18 | -3.18 |  | -2.10 | -3.48 |
| CL11327.Contig1_All | -2.39 | -1.13 |  |  | -2.59 | -1.11 |
| CL6345.Contig1_All | -2.25 | -1.84 | -2.83 |  | -2.02 | -3.03 |
| CL13942.Contig1_All | -2.15 | -2.27 | -3.15 | -2.74 | -2.55 | -3.48 |
| Unigene4401_All | -2.11 | -1.57 | -1.69 |  | -1.14 | -1.38 |
| CL867.Contig1_All | -2.05 | -1.44 | -2.47 | -1.49 | -1.53 | -1.90 |
| CL3113.Contig1_All | -2.04 | -1.56 | -1.98 | -1.19 | -1.88 | -1.75 |
| Unigene11000_All |  |  |  |  |  |  |
| Unigene6175_All | -1.88 | -1.22 | -2.07 |  | -1.86 | -2.29 |
| CL1868.Contig1_All | -1.83 | -2.21 | -3.61 | -1.37 | -1.12 | -2.63 |
| Unigene1922_All | -1.83 | -1.88 | -1.45 | -1.52 |  | -1.11 |
| CL4506.Contig1_All | -1.83 | -1.14 | -1.14 |  | -1.99 | -1.95 |
| CL12008.Contig6_All | -1.68 | -1.99 | -2.00 | -3.91 | -1.37 | -1.14 |
| CL825.Contig2_All | -1.60 | -2.08 | -1.42 | -1.78 | -1.29 | -2.24 |
| Unigene12163_All | -1.48 |  | -1.60 |  | -1.43 | -2.05 |
| Unigene4986_All | -1.46 |  | -1.90 |  | -1.68 | -2.13 |
| CL2656.Contig1_All | -1.37 | -1.93 | -2.46 | -2.42 | -1.94 | -1.53 |
| Unigene13634_All | -1.35 |  | -1.31 |  | -1.56 | -1.65 |
| CL6751.Contig1_All | -1.18 |  | -1.48 |  | -1.43 | -1.41 |
| Unigene1783_All | -1.09 | -1.07 | -1.82 | -1.66 |  | -1.12 |
| CL6751.Contig5_All | -1.08 |  | -1.32 |  | -1.40 | -1.33 |
| CL6751.Contig3_All | -1.04 |  | -1.34 |  | -1.35 | -1.37 |
| Unigene2725_All | -1.03 |  |  |  | -1.08 |  |
| Unigene110_All | -1.00 | -1.69 | -1.33 |  | -1.27 | -1.37 |
| CL1603.Contig3_All |  | 1.26 | -1.02 |  |  |  |
| Unigene61124_All | -2.04 | -9.94 | -1.50 | -3.12 | -1.64 | -2.22 |
| Unigene27302_All | -1.02 | -2.70 | -1.29 | -1.77 |  | -1.31 |
| CL5405.Contig2_All |  |  |  |  |  |  |
| CL9702.Contig2_All |  | -2.34 | -1.41 | -1.06 | -1.30 | -1.17 |
| CL155.Contig2_All | -1.13 | -2.34 | -1.34 | -2.93 | -1.26 | -2.09 |
| CL14594.Contig1_All | -1.58 | -2.30 | -3.46 | -2.78 |  | -1.94 |
| CL12008.Contig5_All |  | -2.20 | -1.76 | -2.32 |  |  |
| CL412.Contig3_All | -1.48 | -2.17 | -2.66 | -2.55 |  | -1.94 |
| CL2067.Contig1_All |  | -2.11 | -1.44 | -1.61 | -1.61 | -1.40 |
| CL400.Contig2_All |  | -1.97 | -1.49 | -2.16 |  |  |
| CL11227.Contig1_All |  | -1.94 | -1.80 | -1.88 |  |  |
| CL39.Contig4_All |  | -1.93 | -1.41 | -1.67 |  |  |
| Unigene5117_All | -1.12 | -1.79 | -2.65 | -1.44 | -2.03 |  |
| CL7741.Contig3_All | -1.69 | -1.78 | -1.04 |  | -1.20 | -2.16 |
| CL1930.Contig1_All |  | -1.44 |  | -1.78 |  |  |
| CL9625.Contig2_All |  | -1.34 | -1.78 | -1.09 |  | -1.22 |
| CL2013.Contig1_All |  | -1.80 | 1.43 |  |  |  |
| CL31.Contig15_All | -2.85 | -1.40 | -10.01 | -8.45 | -1.05 | -4.20 |
| CL1868.Contig2_All | -1.85 | -9.26 | -9.56 | -2.12 | -1.37 | -1.69 |
| CL11758.Contig1_All |  |  | -3.41 | -3.34 | -2.75 |  |
| CL31.Contig1_All |  |  | -2.78 | -4.51 | -1.86 | -2.22 |
| CL6751.Contig2_All |  |  | -1.63 |  | -1.15 | -1.78 |
| Unigene14365_All |  | -1.38 | -1.43 | -2.21 |  |  |
| CL6751.Contig4_All | -1.04 |  | -1.34 |  | -1.45 | -1.33 |
| Unigene12048_All |  |  | -1.15 |  | -1.04 | -1.40 |
| Unigene569_All |  |  | -1.09 |  |  | -1.80 |
| CL7.Contig6_All |  |  | -1.05 | -1.73 |  |  |
| Unigene13648_All |  |  | -1.01 |  | -1.04 | -1.24 |
| Unigene72831_All | 2.76 |  |  | -11.76 |  | -7.33 |
| Unigene26528_All |  | -1.78 |  | -3.21 |  |  |
| CL929.Contig2_All |  |  |  | -2.70 |  |  |
| CL3784.Contig1_All | -1.09 | -1.81 |  | -2.64 | -1.99 |  |
| CL497.Contig1_All |  | -1.88 | -1.19 | -2.34 |  |  |
| Unigene24787_All | -1.63 | -2.88 |  | -2.29 |  | 1.56 |
| CL1852.Contig2_All |  | -1.20 |  | -2.27 |  |  |
| Unigene11026_All |  |  |  | -1.64 |  |  |
| CL13668.Contig2_All |  |  |  | -1.63 |  |  |
| CL1396.Contig1_All |  |  |  | -1.41 |  |  |
| CL12758.Contig1_All |  |  |  | -1.35 |  |  |
| CL3098.Contig1_All | -1.00 |  |  | -1.18 |  |  |
| CL12360.Contig3_All |  |  |  | -1.14 |  |  |
| CL1903.Contig1_All |  |  |  | -1.05 |  |  |
| CL2013.Contig3_All |  |  |  |  |  | 1.68 |
| CL10882.Contig2_All |  |  |  |  |  | 1.18 |
| CL7741.Contig2_All |  | -1.15 | -1.27 |  | -2.05 | -4.35 |
| Unigene1711_All | -2.37 | -1.45 | -1.74 | -2.12 | -2.76 | -4.01 |
| CL7741.Contig1_All |  |  |  |  |  | -2.43 |
| CL1771.Contig2_All | -1.18 |  | -1.26 |  |  | -1.88 |
| CL5040.Contig1_All |  | -1.40 | -1.39 | -1.06 |  | -1.81 |
| CL13501.Contig1_All |  |  |  |  |  | -1.02 |
| CL14110.Contig2_All |  |  |  |  |  | -1.00 |
| up/down | 1/47 | 1/49 | 1/61 | 0/50 | 0/46 | 3/54 |
| total | 48 | 50 | 62 | 50 | 46 | 57 |
